# Supplementary material for: Genomic diversity and structure of prehistoric alpine individuals from the Tyrolean Iceman’s territory
Source: Nat Commun. 2025 Jul 11;16:6431. doi: 10.1038/s41467-025-61601-8 (PMC12254411; doi:10.1038/s41467-025-61601-8)
Supplement: Supplementary file 1 — Supplementary Information [file 41467_2025_61601_MOESM1_ESM.pdf]

# Supplementary Information

**Text S1. Archaeological context**

**Text S2. Radiocarbon dating**

**Text S3. Ancient DNA authentication and alpine dataset**

**Text S4. Kinship analyses and unilinear transmitted markers**

**Text S5. Run of homozygosity (ROH)**

**Text S6. f3 and f4-statisitics**

**Text S7. Timing of the genetic admixture**

**Text S8. Approach used in the analyses to avoid possible bias using the Daicel Arbor Biosciences capture kit**

**Text S9. Analysis of phenotypic SNPs**

**References**

**Figures from S1 to S28**

**Table SM1 and Table SM2**

## Text S1. Archaeological context

The osteological samples analysed in this study were selected from 17 archaeological sites, mostly (15/17) located in the province of Trento (Trentino), with the remaining two further north in the province of Bolzano (Alto Adige/Südtirol, hereafter South Tyrol). These Provinces form the Trentino-South Tyrol region in north-eastern Italy (Fig. 1 in the main text). Overall, these sites are close to each other, and are distributed across various areas in both Trentino (Adige, Vallagarina, Giudicarie and Cavedine Valley) and South Tyrol (Adige and Isarco Valleys; Supplementary Data 1). The maximum distance is between Siusi/Seis (Bolzano) and Grotte di Castelcorno (Trento) being approximately 110 km (Fig. 1) apart.

Most of these archaeological contexts have been discovered in the 1960s. In Trentino, the archaeological excavations have primarily been carried out by the Tridentine Museum of Natural Sciences (Trento), which today is the Science Museum of Trento (hereafter MUSE), as well as by the Archaeological Heritage Office of the Autonomous Province of Trento. Instead, in South Tyrol the excavations have been carried out by the Archaeological Office of the Autonomous Province of Bolzano. Currently, the recovered human remains are stored in various Institutions spread within the territory, such as MUSE (Trento), Buonconsiglio Castle (Trento), Rovereto Civic Museum Foundation, the Office for Archaeological Heritage of the Autonomous Province of Trento and the storehouse of the Archaeological Office of the Autonomous Province of Bolzano. The Archaeological Offices from the two Provinces also authorized the sampling and the analyses of the human remains (Autonomous Province of Trento, Prot. n. 156 15.03.2021; Autonomous Province of Bolzano, Prot. Nr. 636137-25.09.2019 (see also page 17 in the manuscript). A variety of funerary contexts are represented in this work, including those found within caves (e.g., Grotte di Castelcorno), rock shelters (e.g., Madonna Bianca, Paludei di Volano, Romagnano Loc. III), as well as pit burials or within stone cists in open land (e.g., La Vela). The individuals unearthed at these sites were found either in primary (e.g., Ora, Stenico) or secondary depositions (e.g., Siusi, Lasino).

### 1. Funerary contexts in the Trentino area

Data on Mesolithic (ME) burial practices can be challenging to ascertain due to the limited preservation of skeletal remains and burial sites from that period. However, Trentino, along with the eastern part of the Veneto region, has provided the richest documentation of evidence of ME hunter-gatherer groups from the Holocene period in the eastern Italian Alps. In the province of Trento, two of the three Early ME Sauveterrian female burials found in northern Italy have been documented: Vatte di Zambana[1] and Mezzocorona Borgonuovo[2], the latter ascribed

to the Early ME on a stratigraphic basis, but having radiocarbon dates referable to the Early Neolithic (EN)[34]. So far, only one burial of an adult male attributed to the Recent ME, specifically to the Castelnovian complex, has been found at Mondeval de Sora (2000 m.a.s.l., Belluno, eastern Veneto)[3]. The present study has further expanded these findings through the radiocarbon dating ($^{14}C$) of a molar tooth found at Madonna Bianca (section 1.7 in this text). These human remains document the existence of a disturbed burial site referable to the Recent ME, as lithic industry referable to the Castelnovian complex has been found[5].

There are evident gaps in our understanding of local funerary rituals during the EN, especially when contrasted with the wealth of available data from burial complexes dated to the Middle Neolithic (MN, mid 5th mill. BC) associated with the *Vasi a Bocca Quadrata* (VBQ) culture. In the Adige Valley (Trentino area), e.g., groups of graves and/or isolated burials in lithic slab cysts with different type of grave goods, gender-differentiated (e.g., flint arrowheads, ornaments, vessels), have been discovered[6,7]. The most important site of that period is represented by the necropolis of La Vela (section 1.4 in this text) attributed to the earlier phases of VBQ culture (VBQ 1 and 2). Moreover, a single burial found at Isera la Torretta (section 1.3 in this text) was ascribed to a later phase (VBQ3) of this culture that testifies to numerous contacts with the north of the Alps and the Balkan area.

Between the end of the 5th mill. and the first half of the 4th mill. cal. BC, there is a gap in the archaeological record for funerary practices in Trentino, and the earliest documentation comes from the territory of South Tyrol (section 2 in this text).

Concerning, instead, the examination of funerary contexts spanning from the Copper Age (CA) to the Early Bronze Age (EBA), the Trentino region appears as an exceptional geographic area, characterized by a noteworthy concentration of burial artefacts and ritualistic practices[8]. Indeed, the archaeological data mark differences in the environmental choices and burial structures compared to what is known from the MN. From CA, burial contexts such as rock shelters, ravines, debris cones, and substrates at the bases of rock formations or natural cavities prevails over open land necropolises. The continuity in burial practices and rituals between the CA and the EBA, characterized by individual burials in primary depositions, occasionally extends to the persistent use of the same burial area across time (e.g., Mezzocorona Borgonovo, Nogarole). Furthermore, it is recorded the spread of the ritual of secondary and collective burials, predominantly at the base of rock shelters. The secondary depositions, particularly, involve periodic reopening of burial sites including specific skeletal part element removal, perhaps symbolizing the soul's journey to the afterlife during community mourning stages[9]. The deceased could also be accompanied by grave goods, especially elements of ornaments, a practice

that also persists in the BA, having metal objects more frequently present[10]. Moreover, an identified funerary practice exclusive to infants and foetuses, interred within *pithos* (vessels), appears in the CA/EBA, suggesting a cultural influence by the central-eastern European geographical areas[11]. The use of rock shelters and small caves for funerary purposes continues in the BA, and the most significant site in this regard is Romagnano Loc. III and IV (section 1.13). More generally, the deceased were often placed in shallow pits or on the ground surface, later covered with stones. In some cases, the human remains were partially cremated due to their proximity to intentional hearths[10,12].

Here follows some significant archaeological information on the sites where the human samples returned good-quality genetic data after molecular screening and enrichment (Supplementary Data 4). The sites are listed in alphabetic order and distinguished between the Provinces of Trentino and South Tyrol.

**1.1 Bersaglio di Mori**

The site of Bersaglio di Mori (318 m.a.s.l.), also known as Frana del Bersaglio or Frana di Corno, is located in a nodal zone between the major routes of the Garda Lake and Adige River[13]. The documentation collected during the late 1800s by Paolo Orsi on behalf of Rovereto Civic Museum Foundation, and then at the end of the 1960s—a period in which the site underwent several unauthorized excavations—has provided prehistoric evidence spanning from the NE to BA, encompassing both settlement and burial facets.

In particular, a small necropolis was discovered in the fields located below the cone of a landslide in the 1980s, during excavation work for laying a methane pipeline[13]. This finding returned grave goods such as foliate blades, small stone axes, fictile elements, a small vase and two bone pendants, as along with an atrophic deer canine, some copper beads made from rolled tubes were found there[14]. The scattered human remains of at least seven individuals (two adult males, one adult female and four subadults) were also found within what originally appeared to be a mound[13]. One subadult had been instead deposited inside a truncated cone vase.

For this work, only a tooth from the CA individual "cranium A" (BER01, Supplementary Data 1) could be sampled due to the absence of other suitable samples.

### 1.2 Grotte di Castelcorno

South of Castelcorno, near the municipality of Isera (Trento), the Grotte (caves) di Castelcorno site (800 m.a.s.l., Trento) underwent a total of approximately fifteen distinct interventions, encompassing archaeological excavations, accidental discoveries and field surveys[12]. Thus, over the span of 1960 to 1999, a complex cave system of four chambers has been excavated inside the cave, mostly by Rovereto Civic Museum Foundation. Since its initial discovery, human remains have been found, and a total of at least six individuals (three subadults, two adults, and one perinatal) were calculated from tomb 1 and tomb 2 in Chamber 3[15]. In those graves, intrusive artefacts from more recent layers, potentially contaminating the original assemblage, were found due to the partial disturbance during the 1969 excavations. Despite this, some artifacts directly in contact with the human bones, such as an arrowhead, smelting slag, necklace beads, end scraper, and clay spindle whorls, were probably intended as grave goods or offerings[12].

Tomb 1, in particular, showed the presence of stones forming a small mound around the graves, a practice documented in Trentino during the CA and EBA, and the cultural materials align typologically with those from funerary contexts dated to the same chronological period. Dating was also confirmed by radiocarbon dating analyses[12]. The skeletal remains, unfortunately, were severely disturbed by illicit excavations from the 1960s. The least disturbed individual was the subadult ind. 3 (COR1, Supplementary Data 1), presenting limited anatomical connections, a complete cranium and other bones, suggesting that this was likely a primary burial, possibly disturbed in antiquity to make space for a new burial[12]. According to the 1998 archaeological documentation, this individual was probably buried in a crouched position on their right side facing south. The cranium presented a clear sub-circular burned area on the left parietal bone suggesting that the subadult was partly or entirely buried when the fire was ignited, with only a part of it relatively close to the flames[15]. The evidence of burnt animal bones, charcoals, and altered flints in Chamber 3 further supports the documentation of fires being lit inside the cave, possibly linked to post-depositional cultic practices[12]. Despite the difficulty of interpreting rituals from disrupted primary assemblages, according to Battisti and Tecchiati[12] , it seems that these graves, affected by looting and potential other disturbances, reveal a random distribution of bones among different layers, suggesting successive events rather than simultaneous collective burials. Stratigraphic evidence indicates, moreover, ancient alterations to some layers, possibly related to funerary practices involving the deposition or removal of human remains, thus indicating secondary burials and evidence of a skull cult, as in other local burial contexts (e.g., Romagnano Loc. III, Siusi/Seis).

In the present study, two individuals were sampled: ind. 3 (COR01) dated to CA and ind. 2 (COR02) dated to CA/EBA, both from Tomb 1, Chamber 3 (Supplementary Data 1).

### 1.3 Isera La Torretta

The archaeological site of Isera la Torretta (247 m.a.s.l.) is located on the right bank of the Adige River, near the municipality of Rovereto (Trentino). The site was first identified, at the end of the 19th century by Paolo Orsi, who collected archaeological materials and delivered them to the Civic Museum of Rovereto. In 1967, Lawrence H. Barfield conducted several surveys along the foot of La Torretta and at an abandoned quarry on the northwestern part of the hill. The recovered materials, found in secondary deposits, allowed for the first time in Trentino to recognize the presence of VBQ3 culture, also known as "incised and impressed style"[16,17]. Currently, this finding represents the only burial in northern Italy attributable to VQB3, as confirmed by $^{14}C$ dating (Supplementary Data 1). This cultural group was widespread in northern Italy (western Lombardy, Trentino-South Tyrol, Veneto, and Friuli Venezia Giulia regions) and was characterized, in an initial Berico-Euganean phase, by relations with central Italian cultures. In the later phase, contacts with the North-Alpine area (e.g., *Münchshofen* culture) and the Balkan area are predominant, as evidenced by the presence of a copper sheet ornament whose provenance is determined by isotopic analyses[18]. Available dates confirm the antiquity of VBQ3 culture consistent with findings in northeastern Italy[19]. The partial chronological overlap with the latest aspects of the meander-spiral style might suggest a partial coexistence of settlements belonging to groups adopting different styles within the same culture[20].

In 1989 and 1990, the Archaeological Heritage Office of the Autonomous Province of Trento conducted two excavation campaigns in collaboration with the company Co.R.A. (director: Annaluisa Pedrotti). These interventions revealed an impressive stratigraphic sequence over 3 m. deep, documenting five different phases of occupation: the first two (Isera 1 and 2) attributed to the Late NE (second half of the 5th mill. cal. BC); the third and fourth (Isera 3-4) to the Late NE (first half of the 4th mill. cal. BC), and the fifth (Isera 5) to the Early CA (second half of the 4th mill. cal. BC; [21,17]). The earliest horizon, attributable to VBQ3, includes the remains of three - unfortunately damaged - huts (Hut 1-3), by the quarry front. The three huts were not contemporary, and stratigraphic data allowed the identification of various restructuring phases that occurred in the second half of the 5th mill. cal. BC[17]. West of Hut 3, which was the most recent one (4461-4355 BC) and oriented north-south, the skeletal remains of a 36-weeks-old *intra utero* individual (ISE01, Supplementary Data 1) were found. The skeleton was in a crouched position on the left side, with the head facing north and gazing east, in a small space

created among basalt stones that constituted a level of rockfall debris following the inclined bedrock layer (Fig. S1). Unfortunately, the burial had no grave goods and the stratigraphic data do not clarify whether the burial belongs to Isera 1 or 2. The present study made it possible to establish the molecular sex of ISE01, who turned out to be a male (XY, Supplementary Data 2-3). This is the only male individual among the other MN with a different paternal haplogroup (J2a2*) (Supplementary Data 2-4, Fig. 3). The site is located in the southernmost area of the EIAlps compared to the other sites from which the NE samples analysed in this study were recovered (*La Vela* and *Solteri*) (Fig. 1 and Text S1). This individual is also the most recent of the NE analysed and belongs to an advanced phase of the VBQ culture (VBQ3) characterized by cultural links with North Alpine cultures[22,23] . Currently, ISE01 represents the only burial found in northern Italy attributable to this cultural group. Its Y-Chromosome lineage suggests a genetic connection with groups from the easternmost Mediterranean area and Asia.
Other anthropological and archaeological data are summarised in a bachelor thesis titled *La sepoltura del sito di Isera la Torretta (TN). Lo studio antropologico dei resti scheletrici dell'individuo prenatale* (student C. Costanzo, University of Trento).

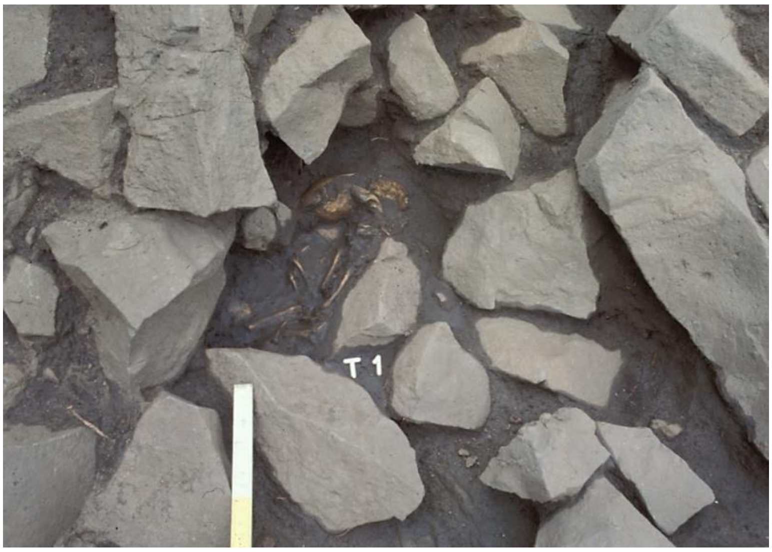


**Figure S1**. **Human remains at Isera La Torretta.** Individual ISE01 found in Tomb 1. Provided by the Archaeological Heritage Office of the Autonomous Province of Trento.

## 1.4 La Vela

The NE site of La Vela (201 m.a.s.l.) is located north-west of Trento. The palaethnological importance of this locality has been known since the 1960s when at least three cist burials were recovered[24]. From 1960 to 2007, the archaeological deposit was identified in nine distinct sectors (from Vela I to IX), following foundation excavations for private construction[17,25,26]. Excavations were conducted from 1975 (Vela II) to 1976 (Vela I-VI) by the Tridentine Museum of Natural Sciences under the leadership of Bernardino Bagolini. Instead, in 1987-1988 (Vela VII), the excavations were performed by the Office for Archaeological Heritage of the Autonomous Province of Trento in cooperation with the University of Trento (director: Enrico Cavada and Bernardino Bagolini). Since 2003, excavations have been (Vela VIII and Vela IX) conducted under the direction of Elisabetta Mottes.

At the site of La Vela, the presence of ME bivouacs (Vela VII and VIII), settlement layers of the EN (Vela I, II, III, VII) assigned to the VBQ culture (in all the sectors) have been reported [27,25]. In 2006, in Vela IX, a cult area from the CA used for funerary practices and possibly also frequented during the EBA was also brought to light[25].

Burial records are attested in different sectors such as Vela I, II, IV, VII, and, currently, a total of 14 burials (eight stone cists, six with stones placed around the bodies) were discovered. Remains of at least one other individual are reported by[28] in tomb 4 (Vela VII). Other bone fragments, considered erratic because represented by a single bone, are also present in tomb 4, tomb 6 (Vela VII) and tomb 1 (Vela IV), suggesting the presence of at least three other individuals.

The identified graves were all oriented south-southeast to north-northwest or south-southeast to north-northwest, as are almost all the structures pertaining to the settlement phase. The bodies were always buried in a pit, located then inside a lithic cist made of stone slabs or with stone placed around the deceased that, in a few cases, could also be interpreted as the wedging of coffins made of perishable material (e.g., wood). Thus, the tombs seem to be grouped in nuclei of two, following a paired pattern (e.g. Vela VII T.3 and T.4) or aligned on the same axis (Vela VII T.1 and T.2; T.5 and T.6). Only the subadult in T.7, housed in a small circular pit, is isolated within an area that was a possible dwelling structure. Additionally, the distribution of tomb types shows a different concentration: to the south-west are the lithic cist tombs, and to the northeast are the graves with stones placed around the bodies (Fig. S2). Both types included individuals of different ages and sexes. The presence of male and female burials from various age groups suggests that this funerary status was not limited to specific individuals, such as

those chosen based on age, but rather extended to particular social groups. However, the limited number of burials (n=14) would suggest that only selected individuals were afforded the privilege of such an interment. Additionally, certain grave goods found with the burials may indicate high status positions. Peculiar to the burials of adult females and children are the ornamental objects represented almost exclusively by spondylus cylinder beads with which necklaces, bracelets and belts were made. Instead, axes and chisels made of polished stone seem mostly typical of male burials, both of young and adult individuals. In most cases the axes are made of jadeite and suggest exchanges with the western Italian areas, i.e. Piedmont and Liguria. Instead, a single chisel of the *Schuhleistenkeil* type is an import from the northern areas[29]. Thus, most of the burials show very rich grave goods, characterised mainly by non-local material (e.g., cinnabar, green stone, spondylus, dentalium, *Serra d'Alto/S.Martino* (region of Apulia, southern Italy) pottery type; Fig. S4). Moreover, the ceramics are all miniaturised, which suggests the production of objects for funerary use only. An exception is the *Serra d'Alto/S. Martino* pottery, which is typical of the *Serra d'Alto* culture but also present in northern Italy especially as a funerary item in female burials[30]. The analysis of the grave goods from the necropolis of La Vela also provided a picture of an autarchic society, which was most probably part of a circuit aimed at the distribution of particularly sought-after instruments and prestigious goods. In summary, nine individuals dated to the MN could be sampled for the current study (Supplementary Data 1): two from Vela I (VEL101 from T.1960/4, VEL102 from T.1960/3); one from Vela II (VEL201 from T.1975); one from Vela IV (VEL401 from T.1976) and five from Vela VII (VEL701 from T.5; VEL702 from T.2; VEL703 from T.4; VEL 704 from T.7; VEL705 from T.6).

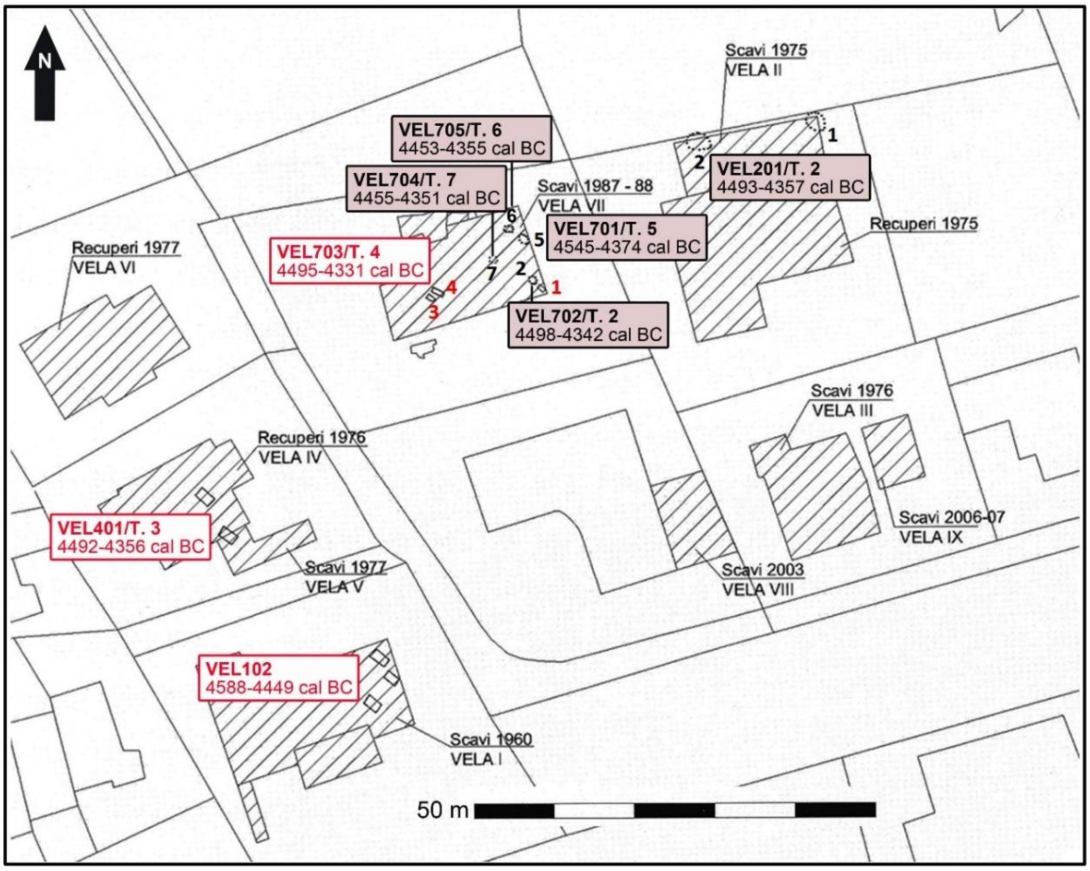


**Figure S2**. **Distribution of the different sectors (I-IX) of the site of La Vela.** In the boxes are the individuals sampled for this project, if $^{14}$C dates were obtained. Dates in red indicate individuals buried in cist graves, while those in black were buried surrounded by stones. Provided by Annaluisa Pedrotti.

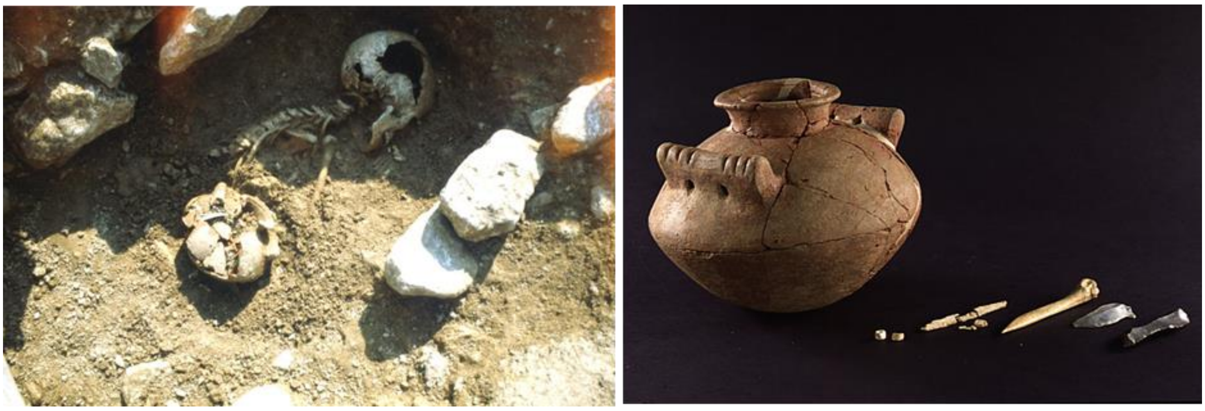

**Figure S3**. **Findings at La Vela VII, Tomb 6.** The individual found in Tomb 6 (on the left), with the grave goods found in the tomb (on the right), including the *Serra d'Alto/S.Martino* pottery type. Provided by the Archaeological Heritage Office of the Autonomous Province of Trento.

### 1.5. La Vela Valbusa

The site of La Vela Valbusa (200 m.a.s.l.) is located north of the NE necropolis of La Vela di Trento (section 1.4 in this text). In 1990, a single burial was found by Leone Fasani on behalf of the Tridentine Museum of Natural Sciences[31]. The discovered alignment of stones was indeed interpreted as a burial mound of ovoid shape, with a north-western/south-eastern orientation, having the greatest width on the northern side. The human remains found in there were possibly in secondary position or "*more probably, an inhumation preceded by a rite of disembowelment*" as documented by Fasani (1990, p.168). To this day, however, this interpretation remains uncertain, due to the unreadable and untraceable archaeological documentation from the 1970s, as also reported by[32]. Rich grave goods were found in the grave (Fig. S4), probably representing the distinguishing features of the individual within their social group [10]. These consisted of 251 items that were mostly related to personal ornaments (e.g., segments of Dentalium shells, ring beads made of other types of shells, bone beads, pendants made of perforated canine teeth of a bear or a deer, two small spirals of copper wire). In addition, fragments of ceramic that belonged to three vessels (i.e. two globular-bodied jugs of the Poladian type) were also recovered. The type of grave and items suggested that the burial was dated to the EBA[31]. Interestingly, after the removal of the mound and the recovery phase of the skeletal remains, Fasani and his collaborators intercepted a flat-bowl smelting furnace, consisting of several hundred smelting slags, mixed with coals and ash, also characterised by the presence of three fragmented nozzles. Chemical analysis, conducted in the 1970s, on two samples of smelting slag enabled the characterisation of slag produced by the semipyritic smelting of a sulphureous copper ore and the remnants of the conversion of an iron-rich metalline[31]. These results suggested, therefore, that the smelting furnace at La Vela Valbusa represented one of the oldest testimonies of metallurgical activity in the Adige Valley in Trentino[18]. Furthermore, the presence of distinct ore remains, probably from different mines, suggested that there were independent smelting centres in the surrounding area[31]. Corrain[33] performed the first anthropological examination of the osteological remains of La Vela Valbusa, assessing that they belonged to a young adult female, 150 cm tall. After that study, the remains were dispersed for a long time. On the occasion of the present study, and especially thanks to their rediscovery by Annaluisa Pedrotti at the Buonconsiglio Castle in Trento, the remains were re-analysed at the Anthropology laboratory of Eurac Research, Bolzano (bachelor thesis *I rituali funerari dell'età del Bronzo Antico in Trentino e territori limitrofi: studio antropologico e paleopatologico dell'individuo di La Vela Valbusa*. Student S. Pinali, University of Trento). Concerning the biological profile, we have confirmed that the human remains belonged to a young adult

(19-21 years old) female individual (Fig. S5), 147-150 cm tall. Genomic analyses further confirmed the sex of this individual (VAL01, XX, female) (Supplementary Data 2-3). Radiocarbon dating conducted for this study places VAL01 within the CA-EBA period (Supplementary Data 1).

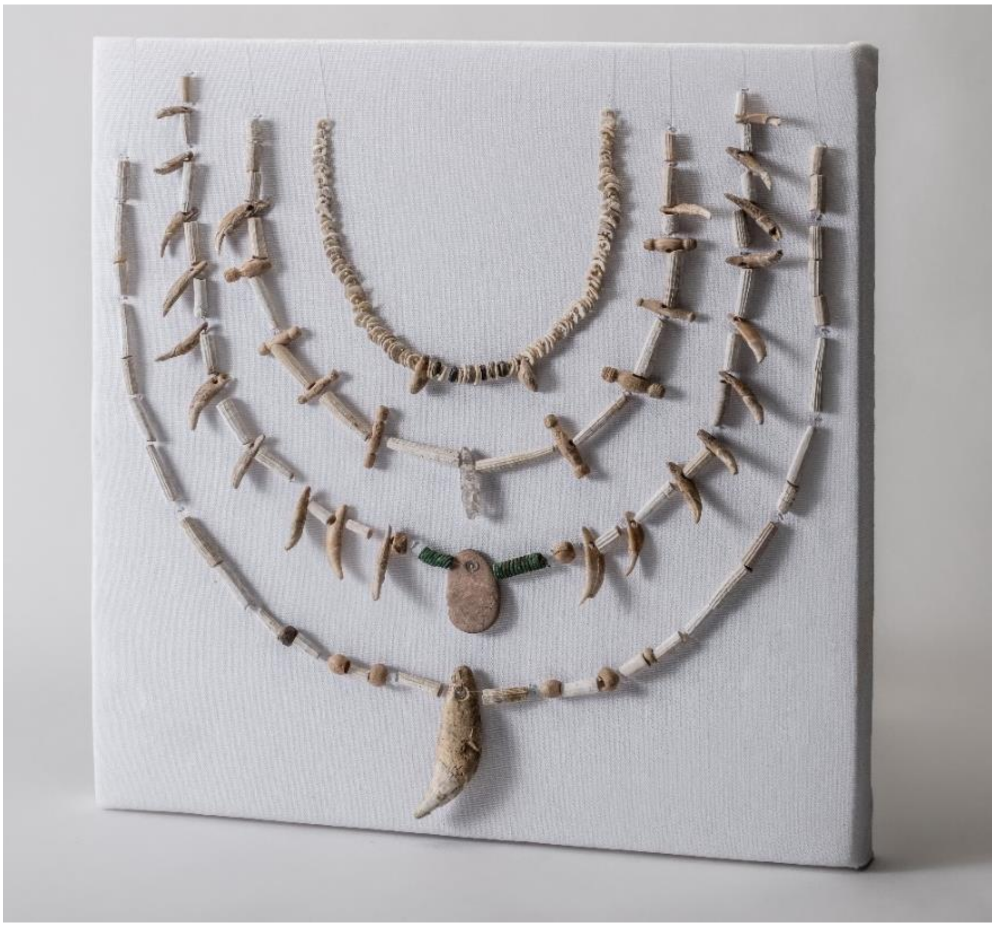

**Figure S4**. **Grave good found at La Vela Valbusa.** Reconstruction of the pectoral belonging to VEL01. Provided by MUSE - Science Museum of Trento/Matteo de Stefano

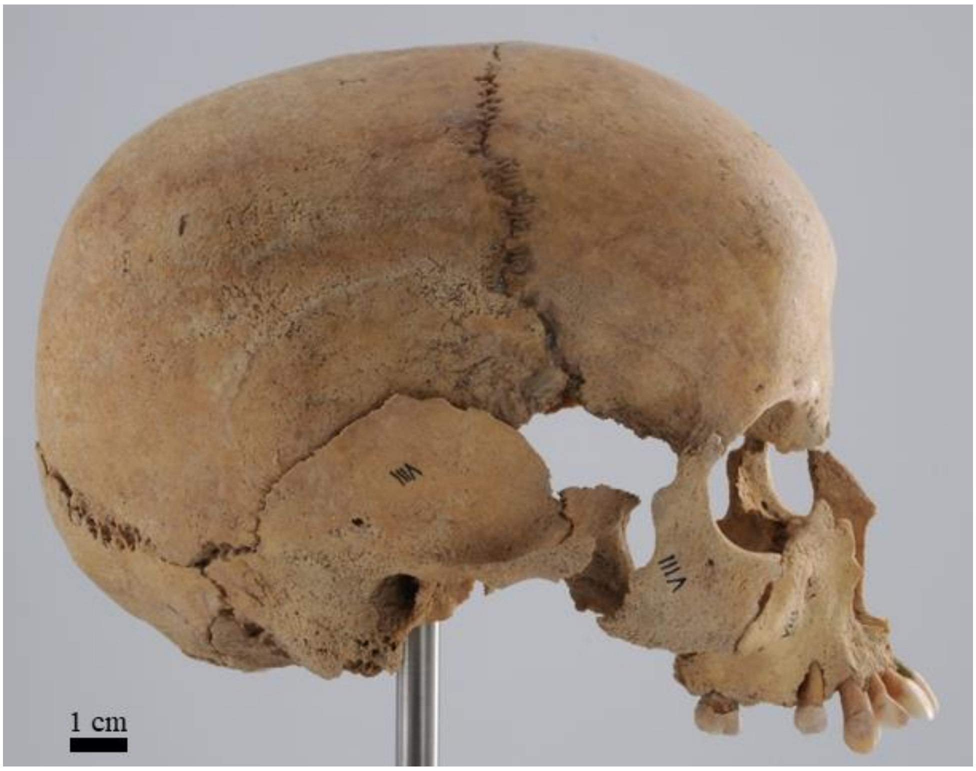


**Figure S5**. **Human remains found at La Vela Valbusa.** Cranium, in right lateral view, of the individual VAL01.

### 1.6. Lasino, Riparo del Santuario

The 'Sanctuary' rock shelter in Lasino (460 m.a.s.l., Cavedine Valley, Trento) has undergone extensive examination in the last century (e.g.,[34–36]) revealing over a thousand years of site frequentation.

The discovery dates back to 1911 when the site was first excavated by Don F. Vogt, who dug an initial trench. Subsequently, the site was reopened by local enthusiasts. First archaeological excavations took place in the 1960s at the base of the sub-vertical wall forming the rock shelter[37,38]. The first phase of the site dates back to the Recent CA and EBA. Instead, the later phase is associated with the discovery, in secondary deposition, of a cranium placed inside a conical cordoned vessel[39]. This finding appears to indicate the skull cult in this area, and the presence of unrelated animal bones could suggest other depositions[10] and potential funerary banquets and ritual offerings[40]. Additional human remains, from 1994 excavations belong to a more settlement-oriented level indicative of a secondary burial. In the subsequent chronological phases, namely the EBA and MBA, the site had a 'settlement' function.

For this study, a sample was obtained from the individual LAS01 (Supplementary Data 1-4) found in the conical cordoned vessel (Fig. S6).

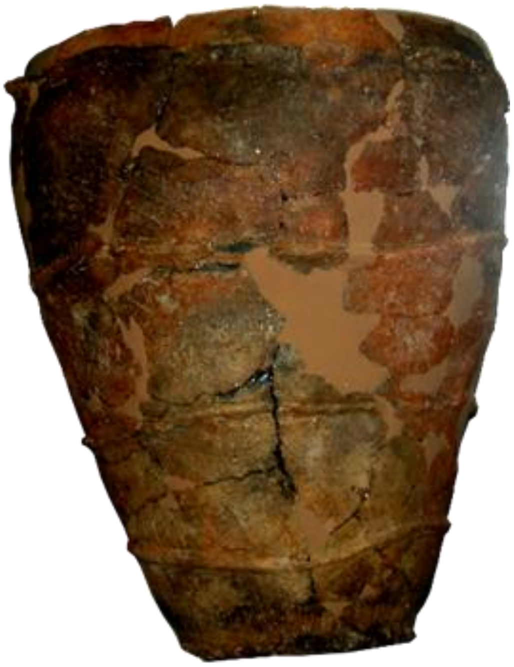

**Figure S6**. **The conical cordoned vessel of Lasino.** This finding contained the skeletal remains of individual LAS01. Provided by Rovereto Civic Museum Foundation/ Maurizio Battisti.

### 1.7 Madonna Bianca

In the rock shelter located near Madonna Bianca (232 m.a.s.l., Trento), the Tridentine Museum of Natural Sciences, following the report of the presence of some lithic materials in the vicinity of the site, began excavation work on contextualizing the discovery[41]. The excavation uncovered a sequence of human activity at the rock shelter, revealing shattered burials and evidence of earlier occupation. This sequence dates back to the Late ME (Castelnovian period) and continues into the EN. Moreover, a stratigraphic gap corresponding to the Middle and Recent NE was reported. A new phase of frequentation was then attributed to the Middle and Recent CA due to the discovery of a flint dagger along the escarpment not far from a niche in the rock shelter (Fig. S7), as well as of a bone ornament of the “Montgomery type” and some bone fragments[41]. A radiocarbon date attributed the discovery to the full CA[17]. Other materials document the continuation of frequentation during the EBA, MBA, and Iron Age[42]. During the investigations conducted in 1985-86, other skeletal remains have been recovered in a disturbed position.

All the preserved human remains recovered from this site (level A E2mq) are today preserved at the Science Museum (MUSE) of Trento. They have been classified into Ind. A (subadult), and Ind. B (adult), and into scattered teeth. The anthropological analyses conducted in this study resulted in a minimum number of four individuals (MNI), including a 5-6 year-old infant (Ind. A, today #25); a mature individual (Ind. B, #26, with ID MAD01 in this study, Supplementary Data 1); and scattered teeth (#27A and #27B) that belonged to at least two adults (MNI).
Radiocarbon dating on the same tooth sampled for the molecular analysis, places individual MAD01 within the Late ME (6,380-6,107 cal. BC; Supplementary Data 1).

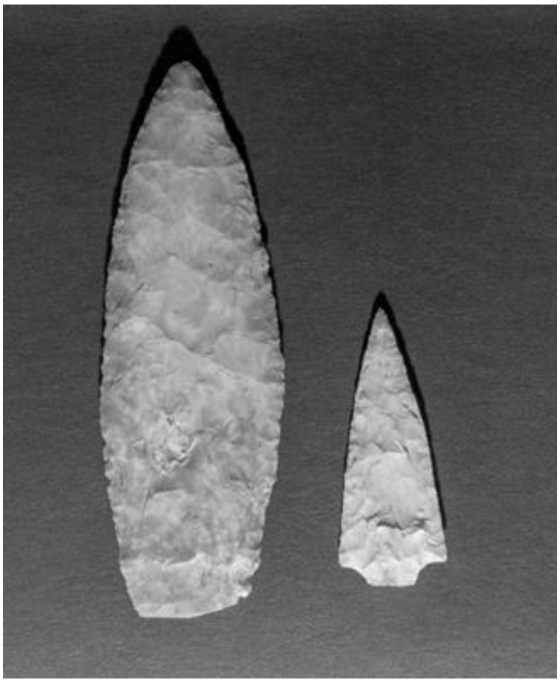

**Figure S7. Madonna Bianca findings.** The dagger blade and the arrowhead in flint found at the site of Madonna Bianca. Provided by MUSE - Science Museum of Trento.

### 1.8 Mezzocorona Borgonuovo

This site is located at the beginning of the Non Valley (240 m.a.s.l.) on the right side of the Adige River in Trentino. At Mezzocorona Borgonuovo an archaeological deposit including a single grave dating to the ME (section 1 in this text) and NE layers, has been excavated[43]. Moreover, the site hosted a burial area dated to the CA, EBA, and possibly to the MBA, located close to a cliff face and under a debris cone. The latter site comprised two areas: a western section, where two vessels (T.1 and T.2) were discovered, and an eastern area, where a truncated cone vase was situated in pit T.4. This vase contained the human remains of two subadults and the

skull vault of an adult, showing some evidence of combustion[10]. The second area, partially affected by excavation activities, was situated within a rocky ridge and revealed three deposition phases. Tombs 5 and 6 were situated on a higher stratigraphic unit, and on the lower level of T.6, large stones were uncovered, forming the covering of T.8[10]. The latter section contained disturbed human remains (Fig. S8), deposited secondarily, comprising at least five individuals, including two subadults. Three individuals from T.8 (MEZ01, MEZ02, MEZ03), dated to CA, were examined in our study. The individual from T.14 (MEZ04) dated to EBA was also investigated (Supplementary Data 1). In addition to these burials, the single inhumation of T.10 was discovered near another area of a small rock shelter. Unfortunately, it was not possible to sample this individual due to the poor state of preservation. Both individuals of T.10 and T.14 had been laid in a crouched position on their left side and with their lower limbs flexed. The graves were surrounded by stones and covered with piles of stones on top of which two vessels had been placed. One vessel was placed in direct contact with the skull and the second vessel was placed a short distance away on the body of the deceased. The vases placed at skull level contained a greater quantity of osteological remains, including those of subadults who died in gestational age, as well as adults and a bronze ring (vase on T.10). The vase on T.14 contained instead the remains of an adult individual and of at least one subadult. The dynamics of the intentional deposition of the vessels on the burials described above testify to a highly articulated funerary ritual, unique to this site[10].

In summary, a total of four individuals, dated to CA and EBA, were sampled for this work, such as MEZ01, MEZ02, MEZ03 from T.8, and MEZ04 from T.14 (Supplementary Data 1).

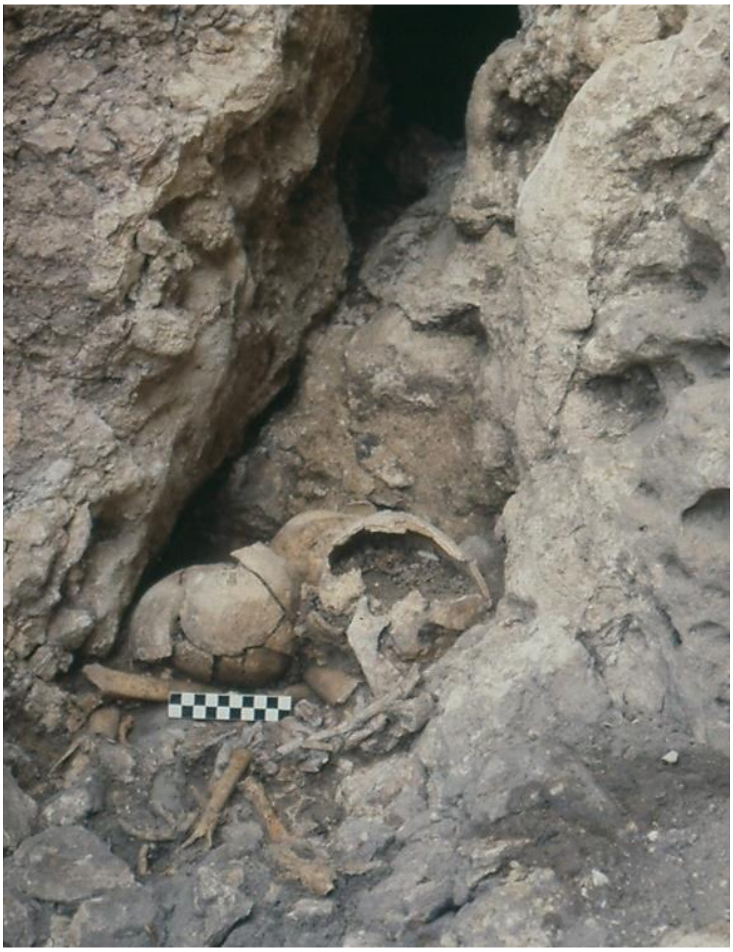

**Figure S8**. **Findings at the site of Mezzocorona Borgonuovo.** Detail of the human remains of the individuals found in Tomb 8. Provided by the Archaeological Heritage Office, Autonomous Province of Trento.

### 1.9 Moletta Patone

At Moletta Patone (95 m.a.s.l.) near a shelter approx. 1 km north of the Arco village, three archaeological excavations were conducted between 1979 and 1981 by the Tridentine Museum of Natural Sciences (director: Bernardino Bagolini). These investigations identified initial occupation of the area during the ME, EN, and in the early phases of the VBQ culture, followed by a period of abandonment. Reoccupied in the Middle CA, the shelter became the site of a burial area.

Anthropological analyses indicate that at least five adult individuals (two males and three females), along with subadults, were buried there[44]. The deceased, interred at different times, were placed on the ground surface near the rock wall in an area where the shelter narrows into a low crevice sealed with stones. At the time of the discovery, the skeletal and cultural remains

were encased in stalagmitic concretions. The preserved bones were mainly vertebrae and elements of the hands and feet, along with numerous teeth[44,45].

The presence of human remains at Moletta Patone suggested the practice of collective funerary rituals, which involved the exhumation, manipulation, selection, and repositioning of bones in secondary burials when new individuals were interred. This practice finds parallels with the ritual present in the group of *Civate* (Lombardy region, northern Italy[46]). The grave goods, whose position relative to the interred is unknown, likely included a simple flat-bottomed vessel with truncated conical walls, five barbed and tanged arrowheads, two Remedellian-type flint daggers (one with a simple base, the other with a tang and barbs), and a *Campignanoide*-type tool. Ornamental items include a plaque made from boar tusk and a shell disc from an oyster. Copper artefacts of high purity (99.7%) consisted of three ribbon-like rods with a triangular cross-section, one with a small ring, and another twisted into a spiral (possibly a bracelet), four round-section wires (one bent into a "U" shape and the others slightly curved), and five sheet-metal beads. Outside the shelter, a small pit containing six flint crescents was found, possibly indicating the shelter's use in the final phase of the CA[17].

In this study, genomic analyses and $^{14}C$ dating were performed on a molar found to belong to an adult female (XX) individual (MOL01) dated to the CA (Supplementary Data 1-2).

### 1.10 Mori loc. Corno

In 2000, rescue excavations were carried out by the Archaeological Heritage Office of the Autonomous Province of Trento in the area of an ancient massive landslide at Mori Loc. Corno (204 m.a.s.l., Trento). Among several boulders, a human skull near the sherds of an almost complete Middle NE decorated vessel (VBQ culture) was found. However, the cultural connection between these two finds, which have not been published yet, remains ambiguous.

As part of the present work, the cranium of the individual MOR01 underwent anthropological analysis at the Anthropology laboratory of Eurac Research, Bolzano. Taphonomic examination revealed whitish calcified concretions on the bones, as well as impressions of small roots within the endocranium. Furthermore, a postmortem fracture was observed on the left parietal near the lambdoid suture, close to the sagittal suture. The cranium (Fig. S9 a, b) is incomplete and fragmented. The best-preserved portions include the calvaria, which encompasses the frontal bone (missing a part on the left side), left (with a missing part of the coronal suture) and right parietal bones, occipital bone, and the complete right and incomplete left temporal bones, including the Petrous Parts (PP). Genetic investigation focused on sampling the well-preserved right PP, which yielded good quality data (Supplementary Data 2-4). The splanchnocranium was almost

absent, except for a small fragment of the right maxilla (molars alveoli 1-2), the nasal bones, and the right zygomatic bone. The mandible and all the teeth were entirely missing. From the macroscopic morphological examination, it was possible to observe that the frontal bone exhibits a slightly pronounced glabella, the parietal bones have prominent eminences, and the mastoid processes of the temporal bones are small, and pyramid-shaped. These features, according to classical anthropological methods (e.g.[47,48]) are associated with a female individual and this result was confirmed by molecular sex determination (XX, female) (Supplementary Data 2-3). The assessment of cranial suture fusion stages[48] showed that the woman died at approx. 55-60 years old. Thus, the presence of the Pacchioni's *Foveae*, in the endocranial surface along the sagittal suture, was not surprising, as the *Foveae* generally become more noticeable with advancing age. No pathological evidence was identified.

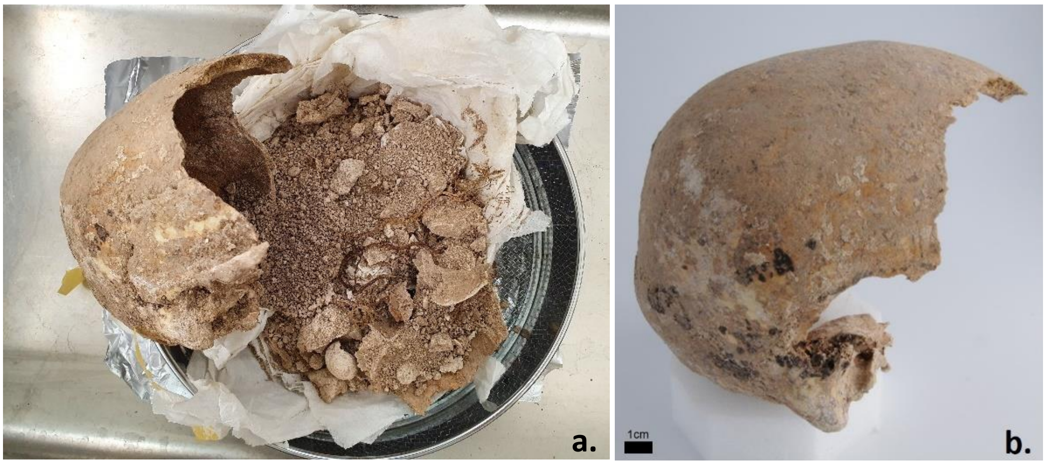


**Figure S9. Human remains found at Mori Loc. Corno. a**. The human cranium of MOR01, before the cleaning phase. **b**. The parietal, temporal (partial), and occipital bones following the anthropological study.

### 1.11 Nogarole

The Nogarole site (300 m.a.s.l., Mezzolombardo, Trento) holds significance for the investigation of burial practices in the EBA. Situated at the base of the Fai della Paganella plateau, the site was discovered in 1985 through the depletion of a detrital cone, revealing anthropogenic sediments subdivided into three rock shelters (Nogarole I, II and III[49]).

Nogarole II unveiled a tumulus burial leaning against the rock wall, hosting a single skeleton located on the left side with flexed lower limbs[49,50]). The recovered grave goods (e.g., 24 necklace elements, a possible truncated-conical vase) dated the tomb to the CA, confirmed by $^{14}C$ dating (NOG201, 2891-2701 cal. BC, Supplementary Data 1). In Nogarole III, instead, sector 1

(west) and sector 2 (east) were excavated. In level 4 of sector 1, a burial ground was discovered, where subadults have been buried inside truncated-cone vessels, then covered with a small circular mound of stones. In sector 2 of Nogarole III, there was also a recess in the rock, creating a kind of small cave where various human remains were found not in anatomical connection. Additional vessels with horizontal cord decorations were placed adjacent to rocky walls and covered with stones, while other fragments were left on the floor. The area was also marked by the presence of small fireplaces, pyres, and some isolated traces of torches[49].

In 1994, further excavations were conducted by the Archaeological Heritage Office of the Autonomous Province of Trento when elements pertaining to the funerary rituals of the CA-EBA were discovered[50]. This dating was confirmed by the $^{14}C$ results of the individual NOG303 (Supplementary Data 1-2). This grave was located outside the crevice in a small depression, and the individual was found in a crouched position, and without any grave goods.

In 2016, just south of the already-known area, a rescue excavation was carried out on a new burial structure located inside a small niche situated along the rock wall and called Riparo 4 (~255 m.a.s.l.). The context of deposition was partly disturbed by modern quarry works, so much so that some of the human remains remained exposed for a long time along the section of the archaeological deposit, at about 10 m. above the current floor. This is the secondary burial of an adult female individual (NOG401, Fig. S10) buried in a north-south orientation against the rock wall and covered originally by a small mound. According to the archaeological evidence the burial can be dated to the CA, specifically to the first half of the 3rd mill. BC, confirmed by the $^{14}C$ (2,865-2,580 BC) (Supplementary Data 1-2). A copper ribbon with a folded end was found near the skull; moreover, a series of ornament elements were brought to light in association with the skeletal remains, including a disc stone bead and five perforated shells of *Columbella rustica*. It is possible that other accompanying objects have been lost[8]. The anthropological analysis of the skeletal remains provided important information relating to the biological profile and pathologies of the individual, such as traumas which could refer to an episode of interpersonal violence[51].

In summary, six samples were selected from the Nogarole for this study (Supplementary Data 1), such as NOG201 from Nogarole II; NOG202, which is potentially from Nogarole II or Nogarole III due to ambiguities in the archaeological records; NOG301, NOG302 (T.2), and NOG303 (T.4) from Nogarole III; and NOG401 from Nogarole IV (T.1, shelter 4).

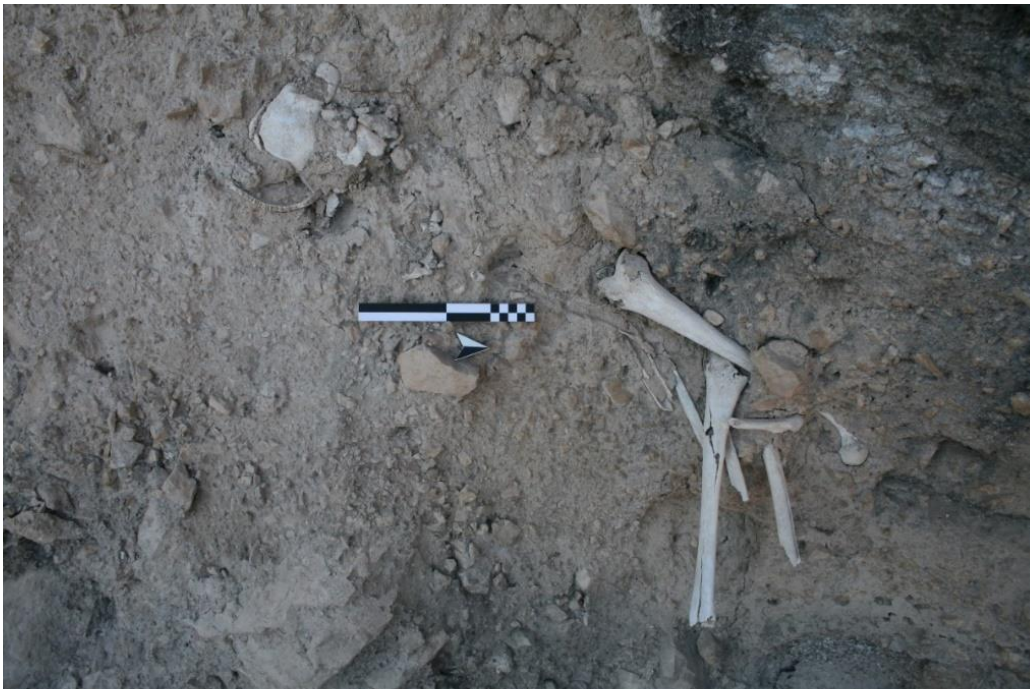

**Figure S10. Archaeological findings at Nogarole IV.** The skeletal human remains of the individual NOG401. Provided by the Archaeological Heritage Office, Autonomous Province of Trento.

### 1.12 Paludei di Volano

Paludei (180 m.a.s.l., Trento) is a rock shelter located on the left bank of the Adige River, near the village of Volano. Excavations at this site were carried out by the Tridentine Museum of Natural Sciences in 1978 (Fig. S11), revealing evidence of occupation from the Late ME period, along with individual inhumation burials[52]. The discovered tombs, adjacent to a rock wall, were two *tumuli* (T.1, T.2) and one stone cist (T.3) that contained three individuals[53]. In particular, the subadult of T.2 was buried with grave goods, such as a long frontal scraper, a pendant made from a wild boar canine, and two miniature bobbin-like objects (one with a truncated cone shape and the other with a sinuous profile). These goods suggested a relative chronology corresponding to the CA[44]. However, $^{14}C$ dating extended the burial dates to the CA-EBA, and the results indicates that the subadults of T.2 (PAL02) and T.3 (PAL01) - sampled for this study- lived and were buried likely during the same period (Fig. S12) (Supplementary Data 1-2), and they were not biologically related (Supplementary Data 5).

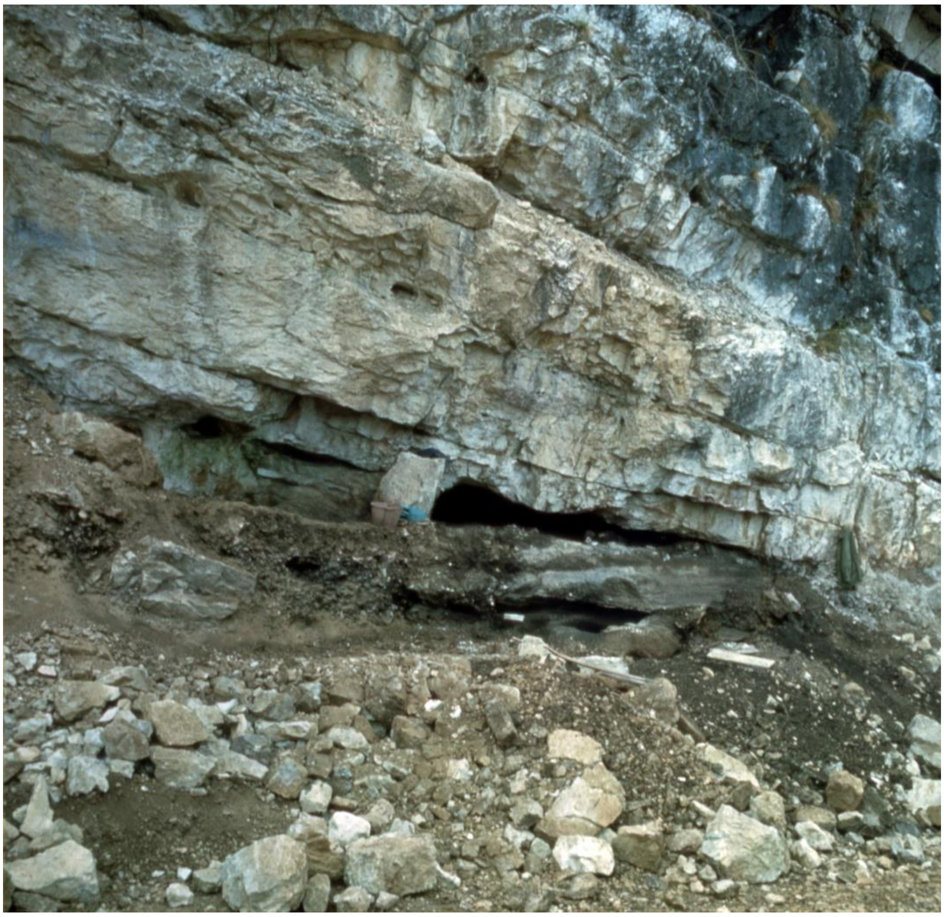

**Figure S11. Rock shelter of Paludei.** Excavation performed in the late 1970s[44]. Provided by the Archaeological Heritage Office of the Autonomous Province of Trento.

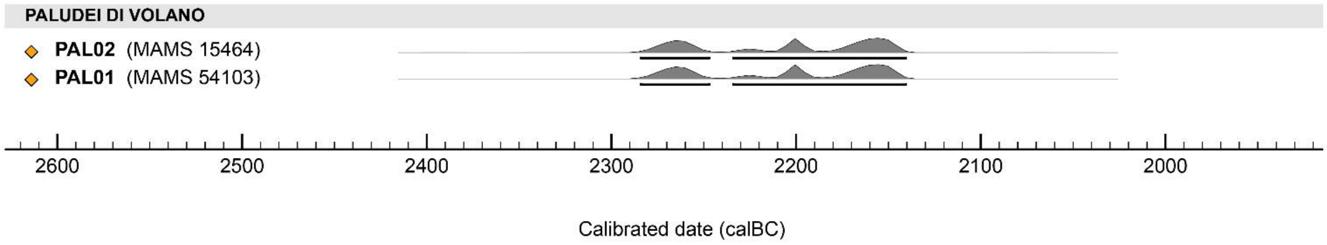


**Figure S12**. **Plot integrating radiocarbon dating**. $^{14}$C dating of the individuals PAL02 (T.2) PAL01 (T.3). OxCal v4.4.4, r:5 Atmospheric data[54].

### 1.13 Romagnano Loc. III and IV

The Romagnano Loc. (225 m.a.s.l.) located near the Rio Bondone (Trento), was discovered in 1969 and excavated by the Tridentine Museum of Trento and the University of Ferrara, Italy[55–57] (Fig. S13). The excavations revealed various stratigraphic units spanning from the Early ME to the Early Iron Age (EIA)[56,57]. Romagnano Loc. stands as a reference site for understanding the Early ME (Sauveterrian, second half of the 10$^{th}$ to 8$^{th}$ mill. cal. BC) and the Late ME (Castelnovian, 7$^{th}$ mill. cal. BC)[58], and also Early NE groups (Gaban) that suggest the continuity of ME traditions and interactions with groups from the Po Plain, northern Italy[59]. The stratigraphy also encompasses the Early and Middle NE periods, revealing pottery of VBQ (1-2) culture. Additionally, it documents a new phase of occupation in the Late NE (layer R) and the Early CA (layer Q), where the presence of a crucible attests to metallurgical activities[17]. An extensive EBA necropolis is also present in layers P (Romagnano Loc. III) and IV (Romagnano Loc. IV) dating to approximately 2300-2100 BC (Supplementary Data 1-2). In total, 17 burials were found in layer P at various elevations (Fig. S14). Even if part of the necropolis was destroyed by bulldozers, it was possible to recover the remains of two tombs, including the burial T.A that contained a vessel which typologically differed from the cordoned truncated-cone vessels used for infant burials[60]. In Romagnano Loc. IV, two additional BA levels were unearthed including the remains of T.1 and T.2. A minimum number of 39 individuals (MNI) was calculated, including 80% (31/39) of subadults (< 12 years old), reflecting high infant mortality typical of the society[61]. However, the potential presence of designated burial areas for distinct age cohorts should be considered[10].

Burials in both sectors were positioned against the rock, often surrounded by stones, often with unclear boundaries due to reuse over the centuries. Some graves, such as T.2, T.4 and T.9, featured the bodies arranged in a curled-up position on the right side, while in one case (T.12), the individual was laid in a supine position. Newborns were placed in a foetal position within

ceramic vessels, both horizontally and vertically, protected by stone tumuli against the shelters' rocky wall (e.g., T.2 in Loc. IV). The burials were primarily inhumations, and there are few examples of burned skeletal human remains (e.g., T.3-T.11). It remains unclear whether this was part of a ritual or incidental, possibly resulting from the action of a fire that had spread in those areas[60]. Some of the described burial practices echo those found at la Vela Valbusa, Borgonuovo di Mezzocorona, and Nogarole di Mezzolombardo[8,62]. In the grave there were individuals in primary, but also in secondary depositions as well as crania only, potentially suggesting a skull cult, like the burial "1969"[56]. That burial was discovered beneath the foundation of a lime wall, where the remains of a cranium were found, compressed by stones that surrounded and covered the pit. There, 60 necklace elements were collected, including a perforated human phalanx[56]. Few other grave goods have been found in some of the graves in Romagnano Loc. III, such as mother-of-pearl beads and perforated teeth. In general, the cultural materials, the funerary contexts (graves in rock shelters and the presence of small stone mounds) and the radiocarbon dating indicate an affiliation with the *Polada A* culture[60].

In summary, eleven individuals dated from CA to EBA were sampled for this study (Supplementary Data 1). Nine were from Romagnano Loc. III (ROM301 from T.11; ROM302 from T.5; ROM303 from T.8; ROM304 from T.1969; ROM305 from T.1; ROM306 from T.4; ROM307 from T.A; ROM308 from T.6 and ROM309 from T.12), and two from T.2 of Romagnano Loc. IV (ROM401 and ROM 402). Nine individuals out of eleven were younger than 13 years old, including five individuals who died below the age at death of 1 years old. Since these individuals were subadults, it was not possible to determine their sex based on classical anthropological analyses. This study, thus, allowed the determination of their biological sex, the majority of which were found to be female (Supplementary Data 2-3).

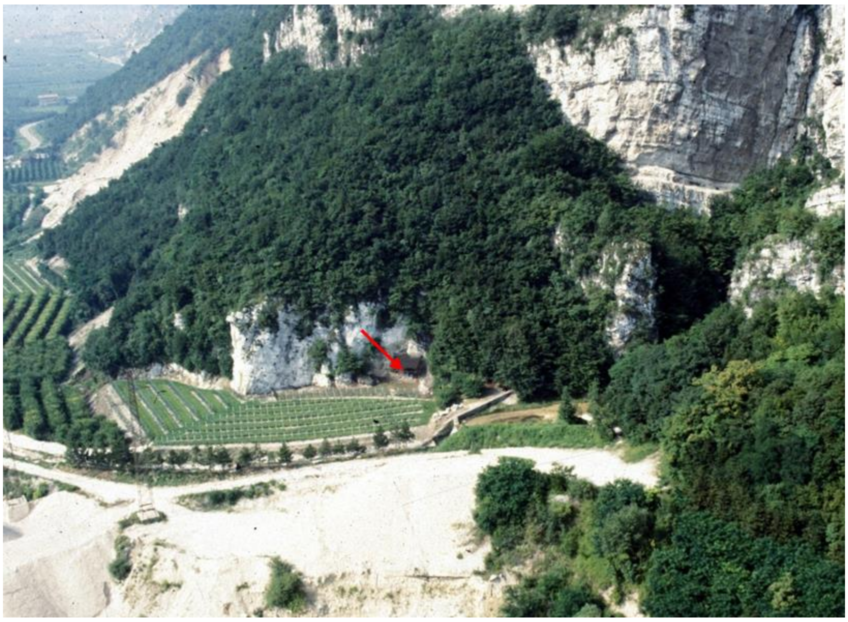

**Figure S13**. **The site of Romagnano Loc. III.** Rock shelter, pointed by the red arrow, of Romagnano Loc. III, Trento. Provided by MUSE - Science Museum of Trento.

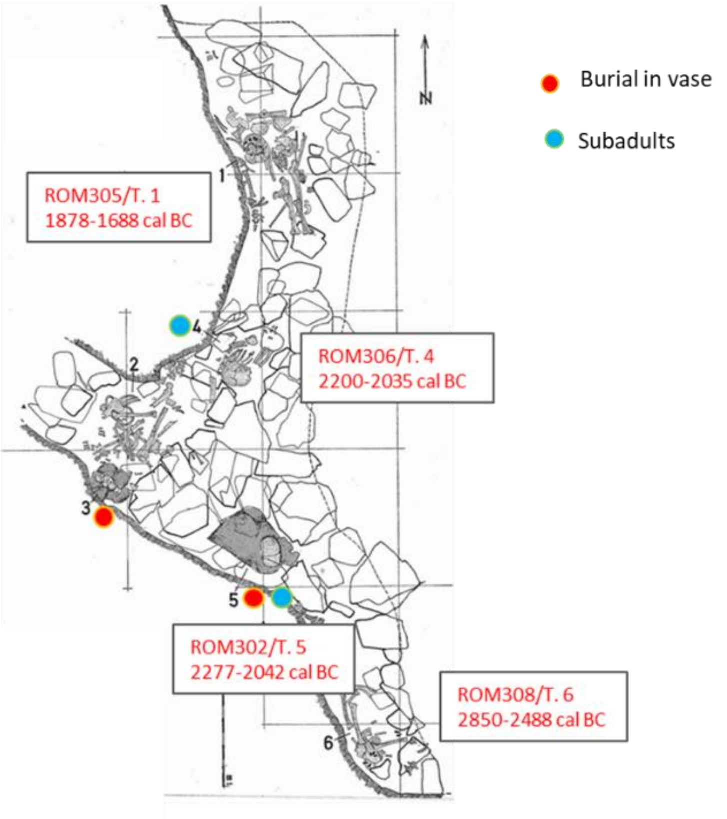


**Figure S14**. **Burial distribution at Romagnano Loc. III.** Drawing of the graves and radiocarbon dating from T.1 to T.6.

### 1.14 Solteri

The site of Solteri (220 m.a.s.l., north of Trento) is located on the left bank of the Adige River, south of Riparo Gaban, and also near numerous quarries exploited in past centuries. In 1962, Renato Perini conducted the first archaeological excavations in the formerly known Mattivi quarry, and he reported the discovery of three tombs partially disturbed. Moreover, he noted that one of the tombs (named "the first") was protected by a stone mound subsequently covered by the remains of a hearth and artefacts attributable to the EBA, *Polada A* cultural phase. Similar examples have been found in La Vela Valbusa and Romagnano Loc. III[60,63]. A review of excavation data and materials was conducted in the mid-1990s by Elisabetta Mottes[64] revealing a habitation of the area mainly during the entire BA, but also with evidence from the EN (VBQ culture), the CA and EIA.

The human remains recovered at this site have undergone various anthropological studies over the years, and according to Corrain and Capitanio[65], individual A (SOL03 in the present work) was a medium-complete skeleton of a young adult male. The commingled remains, instead, were attributed to a minimum number of nine adults (four females and five males) and an undefined number of infants.

In summary, four individuals (SOL01-SOL04), dated from MN to EBA, were sampled for this study (Supplementary Data 1).

### 1.15 Stenico Calferi

In the locality of Calferi, situated in the southern periphery of the municipality of Stenico (666 m.a.s.l.) in Trentino, archaeological remains were discovered in the early 1900s, previously undocumented due to the construction of new dwellings and later the opening of a road[66]. From 1978 to 1981, four excavation campaigns were conducted, funded by the Archaeological Heritage Office of the Autonomous Province of Trento (director: Renato Perini)[67]. These excavations documented two millennia of occupation, including four phases ranging from the BA (Phase 1) to the 10$^{th}$-2$^{nd}$ century BC (Phase 2), the Roman period (Phase 3), and the Early Middle Ages (Phase 4). Regarding Phase 1, an artificial terrace in alluvial moraine gravel revealed the presence of a funerary area, specifically a necropolis with *tumuli* dating to the Late and Middle BA (Fiavè 5b, 6) on the basis of archaeological specimens (Perini 1983, 2001). The *tumulus* extended approximately 13 m. in an east-west direction and housed six tombs with a quadrangular cell orientation in a transverse direction (south-north), delimited by large stones. The tombs T.1, T.2, and T.6 were single graves, while the others were collective, containing a minimum of 13 adult individuals and 14 subadults[67]. Specifically, graves

T.1 and T.6 included burned and calcined human remains. The skeleton in T.2 lacks the cranium, which is generally also missing or incomplete in the other tombs, leading to the hypothesis that it was intentionally removed for a purpose related to the skull cult practices. The bodies, in primary deposition, were placed in a supine position and covered with stones supported by wooden elements. It was initially suggested, during the discovery phase, that the *tumulus* belonged to a family, and this has been partially confirmed in this work (refer to Text S4 for more details). The offering of ceramics (i.e., cups, pitchers, bowls) and animal remains, including the skulls of large mammals (e.g., bears), was associated with the burial ritual, accompanied by the intentional breaking of ceramics *in situ*, classified as *Fiavé* 5b, 6 (advanced phase of the MBA, ca. 1400-1300 cal. BCE).

The individuals sampled for this study, dated from EBA-MBA, were found in tombs T.3 (STE01) and T.4 (STE03, STE04, STE05) (Supplementary Data 1), which were partially disturbed in antiquity.

**1.16 Volano San Rocco**

In 1997-1998, the Archaeological Heritage Office of the Autonomous Province of Trento conducted an archaeological excavation at the base of a rocky wall located near the ancient Gothic church of San Rocco in the municipality of Volano (191 m.a.s.l.), approximately 20 km south of Trento (Nicolis 2004). A study on the cult and funerary site of Volano S. Rocco is yet to be fully published. However, the earliest phase of frequentation has been preliminarily attributed to the formative moment of the EBA (local *Polada A* culture) based on a $^{14}$C dating carried out on a sample of coal (KIA-12455: 3798±26; 2300-2140 cal. BC, 95.4% probability)[69]. However, in the absence of further radiometric dating, this attribution must be considered with some caution.

The site is characterized by the presence of a structured area on the north side initially delimited by a double row of post holes and subsequently by a curvilinear dry-stone wall documented for a length of 33 m. Inside, there were three access gates between 1.5 and 2 m. wide, two of which were flanked by fire areas. Complete bovine skulls (*Bos taurus*) were intentionally placed close to this structure, as well as anatomically connected front and rear limbs of the same animal. The structured area was initially used for the deposition of human bones, in particular skull fragments, flint and ceramic artefacts, selected faunal remains, and abundant archaeobotanical remains including numerous cereal-based food preparations.

In a subsequent phase, a new symbolic use of the area is documented. Indeed, two enclosures made of stones and built along the alignment of the previous structures contain two jar burials

of subadult individuals. The first, covered by a stone mound, yielded the very fragmented remains of at least one vase, as well as the jaw and some long bones of an infant individual close to which owl bones (*Athene noctua*) were collected. Not far away was the second structure which contained the fragments of two truncated cone vessels, one of which held the minute bone remains of an immature (newborn or foetus) individual[8].

At the end of the second phase, significant sandy river deposits confirmed the dating to the BA. In the third phase, a small terrace was prepared in the western area, inside which an adult individual was buried (T.5). The skeleton, in anatomical connection, was placed in a curled position on the right side with the arms flexed on the chest. The deceased, who had no grave goods, was placed directly on top of the structural blocks that formed the small terrace and covered by a small mound of small stones and rubble. A $^{14}C$ dating of a bone sample (KIA-12444) gave the following result: 2791±38 BP; 1014-832 cal. BC, 95.4% probability[69]. In the eastern area, the almost complete skeleton of a small bovine was also found lying on its right side, with its limbs contracted and contained within a fence of stones and pebbles.

The individual from Volano S. Rocco available for the genomic analyses (VOL01, Supplementary Data 1) of this study has been found in the RR129, pinpointing exactly a location on the circular platform (UAS 93). This platform was situated several meters to the north-northwest of T.5 and is notable for the presence of commingled human skulls on its walking surface. This context is dated (relative dating) to the formative phase of the *Polada* culture.

**2. Funerary contexts in the South Tyrolean area**

Unlike Trentino, where funerary evidence is attested since ME (section 1), in South Tyrol, this becomes more evident, albeit limitedly, only dating back to the CA[62,70]. Distinct is the rarity of proper burial structures hosting CA inhumed individuals in the site of Ora/Auer[71,72] being exceptional together with some other findings in the South Tyrolean area: Castel Firmiano – Vorhölle; Egna[73]; Silandro-Talele[74]; Sigmundskron[75] and St. Genesio-Greifensteinerhang, dated to the EBA[76].

The South Tyrolean funerary finds suggest the presence of a sort of a "funerary cultural horizon", having inhumed individuals in rock shelters from Trentino until the Bolzano basin area, while areas designated for incinerated human and animal remains were more in South Tyrol, in Isarco Valley[77]. As suggested by Tecchiati[70], this kind of boundary might also reflect the different geomorphology of the territories or the diverse chemical composition of the soils, leading to varying degrees of preservation of the osteological remains, which are mostly absent in South Tyrol except after cremation.

In this geographical area, unique contexts existed which had both funerary and ceremonial functions such as the megalithic area of Velturno[78] and the necropolis of Barbian Gostner in the Isarco Valley[79], dated around mid 4$^{th}$ mill. BC. In the necropolis of Barbian, in particular, there is evidence of the presence of an incineration tomb inside a vase, the bottom of which has been preserved, probably belonging to a female individual aged 19-40 years old[70].

More generally, in such monumental and ceremonial contexts, broken ceramic vessels have been found, sometimes with traces of combustion as well as mixed with osteological remains. This suggests a complex funerary ritual involving various phases of body manipulation, such as exhumation, burning, and fragmentation. This ritual concluded with the deposition of the burned human remains, often accompanied by cultural objects and animal bones, and it possibly reproduced a sense of closeness to the deceased through the intentional manipulations of the remains[70].

**2.1 Siusi/Seis**

At Siusi/Seis, in an area near the municipality of Castelrotto (1004 m.a.s.l., Bolzano), a human cranium (SIU01) without mandible dated to the MBA was recovered (Supplementary Data 1). It was in a secondary deposition located within a small pit (US15, T.1), which was hidden by a mound of stones and an additional surface covering. The pit was located next to and in phase with a fortification protohistoric wall. Additional osteological remains, attributed to the cranium of T.1 were recovered in an area west of this grave. One hypothesis suggests the grave functioned as a votive deposition[39]. Its placement near the wall strengthens the possibility of a ritualistic act connected to the wall's construction.

The human remains were anthropologically analysed by the archaeological research company of Gianni Rizzi and Co. (Brixen, Italy), and the study resulted in a juvenile individual possibly female[39]. However, the molecular result of the current study revealed that this individual was biologically male (Supplementary Data 2-3). Finally, the cranium displayed palaeopathological evidence, such as *cribra orbitalia* and *cranii*[39] (Fig. S15).

From a cultural perspective, the individual SIU01 likely belongs to a horizon corresponding to that of the *Inneralpine Bronzezeitkultur* (IBK), which developed both north and south of the Alps, spanning the Early to Middle Bronze Age, but is still little investigated [80,81]

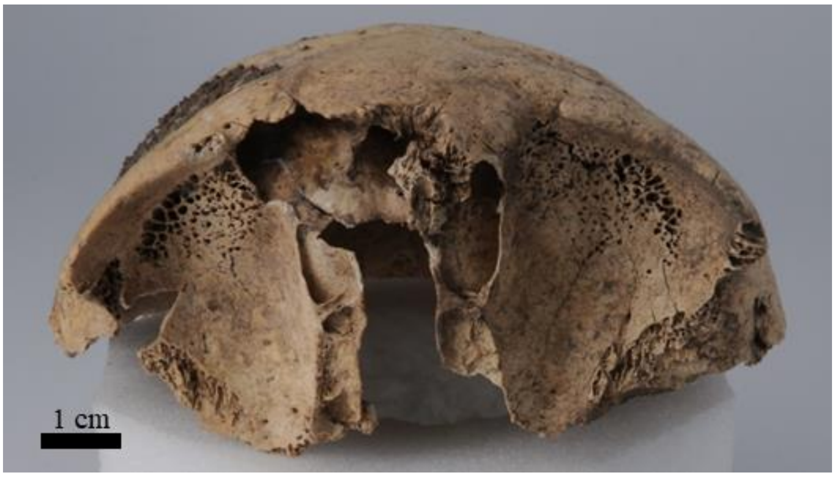


**Figure S15**. **Human remains found at Siusi/Seis.** Detail of the frontal bone of SIU01. The orbit vaults display *cribra orbitalia* as reported in [82].

## Text S2. Radiocarbon dating

Radiocarbon dating ($^{14}C$) was carried out on a minimum of 500 mg of 37 alpine samples at the Curt-Engelhorn-Center Archaeometry gGmbH (CEZA, Mannheim, Germany, https://ceza.de/en). These samples, carefully chosen from those previously selected for genetic analysis, included bone remains (e.g., petrous part of the temporal bone, occipital bone) or dentine from teeth (Supplementary Data 1). A pretreatment step involving various organic solvents preceded the application of the HCl/NaOH/HCl method[83] to eliminate all contaminating elements. The resulting insoluble fraction was then used for measurements. The samples material underwent combustion to $CO_2$ in an Elemental Analyzer (EA), and the $CO_2$ was subsequently converted to graphite. Accelerated mass spectrometry (AMS) of the MICADAS type was then employed to measure the samples, calibration standards (Oxalic Acid-II), blanks, and control standards. Calibration (cal.) of the radiocarbon ages (before present, BP) into calendar years (before current era, BCE or current era, CE) was completed using the software OxCal 4.4 and using the dataset of the IntCal20 calibration curve[84]. Two individuals only (MEZ01 and MEZ02) did not have sufficient preserved collagen for dating. The remaining samples were calibrated (sigma 2) with a 95% probability, except for one individual (ROM308), whose calibration accuracy did not exceed 68%. Moreover, for five individuals (ROM309, ROM401, VOL01, VEL101 and SOL01) for whom $^{14}C$ dating was not obtainable, the relative date based on archaeological information was considered. Finally,

dating information for twelve samples included in our dataset (see Text S3) was already available in the literature, with corresponding references provided in Supplementary Data 1. These data were recalibrated using the OxCal program (https://c14.arch.ox.ac.uk/oxcal/OxCal.html).

In the end, dating was available for a total of 48 alpine individuals considered in this study chronologically assigned from the Mesolithic to the Middle Bronze Age (Supplementary Data 1 and Fig. S16).

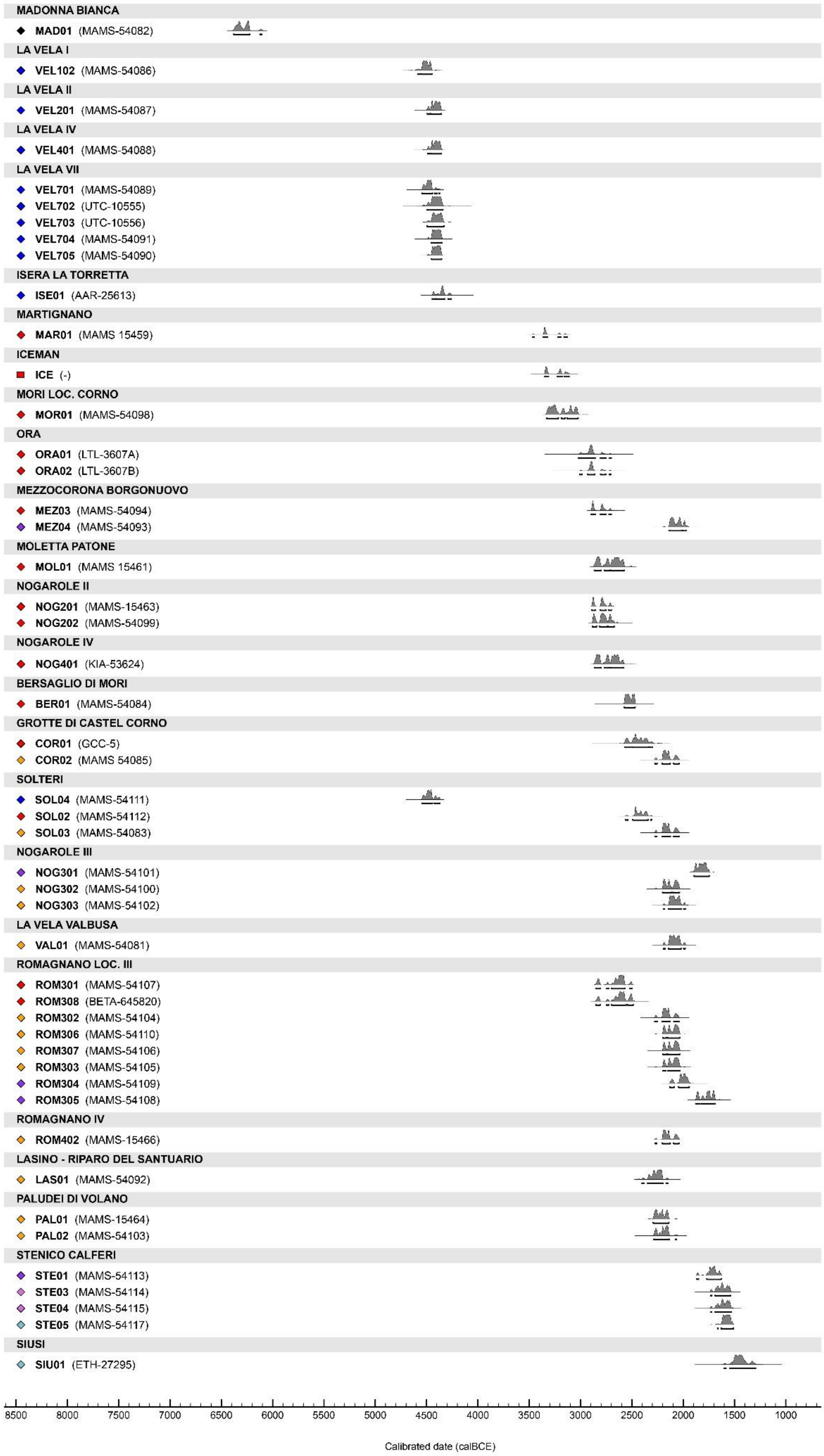


**Figure S16. OxCal plot**. The plot displays the calibrated radiocarbon dates on 48 alpine samples (see Supplementary Data 1), spanning from ME (MAD01) to MBA (SIU01) organized by archaeological site and a single individual (ICEMAN).

## Text S3. Ancient DNA authentication and alpine dataset

Paired-end genomic libraries constructed using DNA extracts from the 52 alpine samples (Pars petrosa or teeth) were tested for their content of endogenous human DNA (HR) using shotgun sequencing (Supplementary Data 2). Most samples show typical ancient DNA (aDNA) damage patterns and short read length (from 41 to 123 bp) with good preservation of HR (from 1.1% to 75.5%; average 28.5%), except for four (MAR01, VEL401, VEL704, VEL101) which were excluded from the study (Supplementary Data 2). All the remaining samples (except STE01, not available for capture) with an HR>1% were selected for the enrichment of ~ 1.24 million SNPs across the human genome[85] as well as 49K additional sites on the Y-Chromosome and the complete mitogenome (Methods and Text S8). Bioinformatic analysis of the sequenced capture data confirmed aDNA authentication for all samples (apart from STE03 which shows a very low deamination value), and increased percentage of HR after capture (from 89% to 96.1 %) (Supplementary Data 3). Additionally, three individuals (VEL703, VEL201 and MEZ01) were found to be contaminated (from 9% to 19% using mtDNA data). However, after filtering the contaminated reads with PMDTools (see Methods), only one sample (VEL201) was discarded due to the small number of remaining SNPs (< 20,000 SNPs sites on the 1240K panel SNPs). Finally, we obtained 47 new alpine genomes (45 capture and 2 shotgun) with a mean coverage comprised from 0.012X to 0.587X, while the number of SNPs on the 1240K panel ranged from 33,981 to 1,046,042 SNPs sites. Instead, the coverage of the mitogenomes varies from 3.18X to 538X. Available data from other three alpine CA individuals[72,86] were included for subsequent analyses (Supplementary Data 4). The complete dataset (50 individuals) comprises a similar number of females and males (26 XX and 24 XY, respectively), including the subadults for which the anthropological analysis did not allow the sex to be established (Supplementary Data 1, Supplementary Data 4).

## Text S4: Kinship analyses and uniparental markers

Kinships between pairs of individuals in the total alpine dataset (N=50) were revealed by three different methods (READ, TKGWV2 and KIN) (Supplementary Data 5-8 and Fig. S17). READ[87] calculates and normalises the mismatch rate (P0) between pairs of individuals across the whole genome while TKGWV2[88] uses a likelihood-based approach on genome-wide variants and both methods can infer pairwise relationships up to the 2$^{nd}$-degree. Instead, KIN[89] use a Hidden-Markov-Model-based approach to identify identity-by-descent fragments to

estimate up to the 3rd-degree of relatedness from ancient DNA data (down to 0,05x coverage). Additionally, it allows differentiating between sibling and parent-child relationships for 1st-degree relatedness.

In this study, only kinships based on full consistency between at least two of the applied methods were considered. Moreover, information on the uniparental markers (Y- Chromosome and mtDNA data) was taken into account to establish the type of relatedness, at the paternal or maternal level (Supplementary Data 5).

Indeed, four couples were clearly related at a 1st-degree level. For two of them from CA (ORA01/ORA01 and MEZ01/MEZ02), one method (KIN) specifically detected a parent-child relationship while for the other two pairs from CA and MN (NOG201/NOG202 and VEL701/VEL705, respectively) it detected a relation among siblings. The related adult males from the site of Ora in South Tyrol (ORA01/ORA02), carried the same Y-Chromosome haplogroup G2a2b*, which is present in almost all males analysed in this study, but different mtDNA lineage (J1c+16261+189 and K1a) indicating unambiguously that they were related along the paternal line and were most probably father and son[72]. On the other hand, the other couple of parent-child from CA identified by autosomal data consisting of the mature female individual (MEZ01) and the young adult male (MEZ02) buried in tomb 8 at Mezzocorona Borgonuovo in Trentino (Text S1), were possibly mother and son. In fact, they share the same mtDNA haplogroup (H2) even if the haplogroup assignment for these individuals is doubtful due to the high number of missing positions (MEZ01) or low-quality assignment (MEZ02) (Supplementary Data 2-4).

The related pair of siblings from CA, including NOG201 (male, 13-14 yo, 2,891-2,701 BC) and NOG202 (female, foetus-newborn of 38-40 gestational weeks, 2886-2673 BC) from level 5 at Nogarole II site in Trentino, were possibly brother and sister as also confirmed by their identical mtDNA haplotype of the same haplogroup (H5a4a1). The same kind of relatedness can be likely inferred for the couple VEL701 (male, 13-14 yo, 4,545-4,374 BC) and VEL705 (female, 7-8 yo, 4,453-4,355 BC) that carried the same maternal lineage (H). These individuals were from the MN site of la Vela VII (Trento), and they were buried in two stone-lined single tombs (T5 and T6, respectively), located in the same area (eastern) of the necropolis.

Regarding the alpine samples which were found to be related at a 2nd-degree level, these include the MN pairs SOL01 (adult female) and SOL04 (female, 20-25 yo, 4541-4369 BC) from the site of Solteri in the Trentino area which were possible related at the paternal level since they had different maternal lineages (mtDNA haplogroups J1c8a and K1a+195, respectively). Moreover, the other pair comprises samples ROM302 (female, 4-5 yo, 2277-2042 BC) and

ROM303 (female, newborn, 2198-2034 BC), which are dated to the transition phase between CA and BA and were recovered from two different graves (T5 and T8, respectively) at the archaeological site of Romagnano III (Trentino) (Text S1). They were related at the 2nd-degree level along the paternal line since maternal relatedness could be excluded due to their different mtDNA lineages (haplogroups H3k and X2b+226) (Supplementary Data 2-4). Interestingly, the immature skeletal remains of ROM303 were found among the fragments of a truncated cone vase positioned vertically in the grave. During the recovery phase it was observed that the individual had been placed head down in the vase.

Three other pairs of individuals (PAL01/PAL02, NOG302/NOG303 and SOL03/COR01) were found to be possibly related to a different degree or were unrelated according to different methods (Supplementary Data 5-8). In fact, TKGWV2 and KIN found most of these samples related (at 2nd or 3rd-degree levels with differences among the two methods), while READ indicates no relatedness in all cases. Taking these differences into consideration, the three mentioned couples of possibly related individuals were considered as not related and were used for comparative analyses (Supplementary Data 5).

A special case could be represented by the EBA/MBA individuals recovered from the site of Stenico Calferi in Trentino. In fact, all four males, two adults from single and multiple graves T3 and T4 (STE01 and STE03) and two subadults from grave T4 (STE04 and STE05) resulted related to each other at different levels (1st, 2nd or 3rd-degree, depending on the applied method). Two of them form a possible parent-child couple consisting of the adult STE03 (1,731-1,539 BC) and subadult STE05 (1,663-1,511 BC) (Supplementary Data 5). Furthermore, with the analysis of the unilinear transmitted markers we found a similar Y-Chromosomal haplogroup (G2a2b2a1a1b*) but different maternal lineages (H1q, H6a1a, and K2a) in most individuals from this archaeological site (except maybe for STE01 and STE03 which share the same mtDNA haplogroup H1q but with quality assignment < 90% in STE01), suggesting relatedness on the paternal line. However, these results on *Stenico* should be taken with caution since, for two samples (STE01 and STE03), only shotgun data are available. Moreover, for the pair STE03/STE05, results obtained by TKGWV2 are based on too few SNPs (for STE03, <10.000) (Supplementary Data 6) while the KIN method found a Log likelihood ratio < 1 for this couple (Supplementary Data 8). The same applies to two other pairs, including STE03/STE04 and STE04/STE05. Additionally, the last two possible related males from Stenico, STE01 and STE04, are shown in READ |Z| < 1 (Supplementary Data 7). Finally, the different levels of resolution of the Y-Chromosomal haplogroup G2a2b2a1a1b* in these samples as well as the high frequency of this lineage in the alpine dataset (Supplementary Data 2-4), should be taken

into account in the interpretation of these results for this site. However, despite some uncertainty about these relationships, as a precautionary measure, only one individual from Stenico (STE05) with the highest number of SNPs was included in downstream analyses (Supplementary Data 5).

In conclusion, kinships were found in individuals from all chronologies and involved both sexes, with a tendency to have more maternal than paternal close ($1^{st}$-degree relationships) kinships. The related individuals were interred in the same archaeological site (in single or multiple graves) but apparently without a clear pattern. However, we do not find extended close biological relationships (up to the $3^{rd}$-degree) among the alpine individuals who resulted to be mostly unrelated. Nevertheless, we cannot exclude that lower-grade kinship cases may exist between specimens analysed in this study.

## Text S5. Run of homozygosity (ROH)

We detected runs of homozygosity (ROH) in our prehistoric alpine individuals using the method implemented in hapROH[90] (Fig. S18) to estimate their level of inbreeding and the effective population size (Ne). The analysis was performed on 46 alpine individuals, including possible relatives estimated by kinship analyses, but excluding four samples with a number of SNPs on the 1240K panel below 400.000 (MEZ01 and VEL703) and the two samples for which only shotgun data are available (STE01, STE03) (Supplementary Data 4 and Supplementary Data 9).

A long ROH of length >20 cM ($ROH_{>20}$) reflects the union of recent kin, whereas a short ROH of length between 4 and 8 cM ($ROH_{[4-8]}$) reflects background relatedness reflecting the local population sizes with a high level of $ROH_{[4-8]}$ indicating low population size[84]. First, the ME alpine individual (MAD01) shows the highest sum of ROH (sROH = 87 cM)] as well as one of the highest sum of short ROH (Mean $sROH_{[4-8]}$ = 25 cM), suggesting a smaller population size during ME (Fig. S18A, Supplementary Data 9).

The sum of short ROH ($sROH_{[4-8]}$) and mean estimates of $sROH_{[4-8]}$ for each individual, from MN to MBA, was plotted indicating the effective population size variations through time in the EIAlp (Fig. S18B). The results show a decrease in the values of the $sROH_{[4-8]}$ from MN to CA, with a drop in the transition between these two periods, indicating an increase in the local population size during NE (mean $sROH_{[4-8]}$ for MN = 13.46 cM and for CA = 4.38 cM ) as expected and often observed for farming communities[90]. This is followed by an increase of $sROH_{[4-8]}$ values in the following periods with reduction in the local population size and with

similar $sROH_{[4-8]}$ values and population size trend from CA-EBA to MBA (mean $sROH_{[4-8]}$ for CA-EBA = 8.39 cM, for EBA = 7.66 cM and for MBA =9.1 cM ) (Fig. S18B, Supplementary Data 9).

Concerning the level of inbreeding in our prehistoric alpine individuals, four of them [COR01 (CA, male), NOG301 (EBA, male), ROM308 (CA, male) and SOL01 (MN, female)] show ROH values superior to 20 cM. Indeed, the sum of $ROH_{>20}$ for these samples ranged between 21.6 and 32.6 cM suggesting that these individuals are offspring from $2^{nd}$-degree cousins (Fig. S18A).

## Text S6. $f_3$ and $f_4$-statistics

The migrations of NE farmers in Europe followed two main routes: one along the Mediterranean coastline to Iberia and the other along the Danube River to central Europe (e.g.)[91,92]. The first migration has been associated with the *Impressed Ware* culture while the one along the Danube River with the *Linearbandkeramik* (LBK) culture (e.g.) [93,94]

In order to explore possible genomic affinities of our alpine samples to cultural groups associated with one of these routes, we performed $f_3$ and $f_4$-statistics based on the results of our PCA analyses. Looking at the NE samples, this plot shows that the alpine genomes are between NE individuals from Central Europe and the Balkans and NE samples from Spain (zoom in Fig. 2A and Fig. S20A).

In outgroup $f_3$-statistics (Supplementary Data 13, Fig. S28) our samples were grouped based on chronologies and ancestries (Fig. S24) and were compared to populations from similar or previous periods. The MN alpine group shows a slightly higher genetic affinity with the early NE group from Croatia of Impressed Ware culture[95], but also to the MN group from *Baalberge* culture in Germany[85], suggesting that both routes may have involved this alpine region. This is supported by the *$f_4$-statisitics* analysis (Supplementary Data 14-15 and Fig. S27) which was also performed by comparing the alpine individuals separately or grouped (Fig. S24), with populations belonging to the Impressed Ware culture or EN from Spain and the LBK.

We observed that only a few alpine prehistoric samples, mostly from the CA-EBA group (e.g., SOL03, ROM303, ROM308, and PAL02), display a higher genetic affinity with Spain_EN than with Hungary_MN_LBK (with $|Z| > 3$). However, in general, results showed no clear signals ($|Z| < 3$) of excess in genetic affinity to either one of the two groups, Spain_EN or LBK (Supplementary Data 14-15 and Fig. S27).

Nevertheless, it is likely that the genomic data and tests used here are not powerful enough to distinguish the two hypotheses and routes, as already pointed out in other studies (e.g.)[96].

## Text S7. Timing of the genetic admixture

Admixture time was determined using the software DATES (Distribution of Ancestry Tracts of Evolutionary Signals)[97], which measures the ancestry covariance within the genome of an individual taking into account the difference in allele frequency between two ancestral populations. The analysis was performed on the alpine individuals or groups defined according to their ancestry and chronology (Fig. S24, Fig. S26).

The admixture time between Villabruna and EHGs used as sources in MAD01 could not be estimated due to the high standard error (superior to the admixture time). However, when a group of WHGs (Luxembourg_ Loschbourg.DG; Italy_Villabruna; Spain_Labrana; Switzerland_Bichon.SG; Italy_OrienteC), rather than the individual of Villabruna only, were used for the estimation to improve the inference, the admixture time between the two HGs group ranged between 13,745 to 8,326 BC (median 11,031 BC) (Supplementary Data 20, Fig. S26). Then, we confirm by qpAdm analyses that this model of admixture in MAD01 is fitting (p-value= 0.048) (Supplementary Data 16). Our finding agrees with previous study which has been shown that ME individual from Europe dated < 7.5 Ka carried EHGs-related ancestry[98]. For the Southeastern Iron Gates population, the admixture time between ME and EHG was found to be ~9,200 BC (± 1,800 BC)[97] while for Sicily[99] was estimated around 8,800 years ago. We also estimate the admixture time between the HGs (MAD01) and early NE Anatolian ancestries (Turkey_N) as sources in the alpine genomes from three groups (MN, CA, CA-EBA) and one MBA alpine individual (STE05) (Fig. S24, Fig. S26 and Supplementary Data 20). For the remaining groups or individuals (CA-EBA_1; CA_EBA_2, EBA; ROM305 and SIU01), estimations were not realistic (admixture time predates 10$^{th}$ millennium BC) or were technically irrelevant (standard error superior to the estimated time of admixture).

As one would expect, we found an earlier time of admixture for the MN group (between 6,558 to 4,456 BC, median 5,507 BC) than for CA (4,811-2,574 BC, median 3,692 BC), CA-EBA (5,601-2,094 BC, median 3,852 BC), and STE05 (5,065-2,722 BC, median 3,893 BC) (Supplementary Data 20). However, due to the large standard error observed in all the cases, it is difficult to confirm that the admixture really happened at two different times, which will indicate continuous or several pulses of admixture instead of a single one[97].

Also in this case, to improve inference we used a group of WHGs (Hungary_Koros; Germany_Blatterhohle; Switzerland_Bichon.SG; Croatia_VelaSpila; Italy_OrienteC; Falkenstein; BerryAuBac; Iboussieres25-1; Iboussieres31-2; Rochedane; Drigge, MAD01) rather than the single local ME individual (MAD01) as a source of HGs (Supplementary Data 20) and we confirm the models by qpAdm analyses in most cases (p-value > 0.01), except for

the PrehAlps_CA_EBA group  (Supplementary Data 19) (Fig. S26). The results agree with the previous ones (with MAD01 and Turkey_N as sources) but are more accurate with lower standard errors, although there is still some overlap (Fig. S26). Indeed, the admixture time for the MN group ranged between 6,073 to 5,117 BC (median 5,595 BC); for CA between 5,646 to 3,817 BC (median 4,731 BC); CA-EBA from 5,157 to 3,904 BC (medium 4,531 BC), and for STE05 from 4,648 to 2,711 BC (median 3,679 BC) (Supplementary Data 20). The estimated admixture event in the CA Iceman, using different NE proxies and 29 years for one generation, was found to be 4880 ± 635 BC or 4400 ± 432 BC[100].

In addition, the admixture dates between local CA or CA-EBA groups used as sources together with the steppe-related ancestry (Yamnaya population), were estimated in the groups or individuals carrying this component (Supplementary Data 20, Fig. 4 and Fig. S27). The time of admixture in LAS01 (between alpine CA and steppe-related ancestry) was estimated between 2911,9 and 2218,7 (median 2565,3 BC). Lastly, our estimation in SIU01 ranged between 2,453 and 1,854 BC (median 2,154 BC), within the same range as LAS01 (Supplementary Data 20). However, based on the general results found for SIU01, we cannot exclude a different origin for this sample compared to the other alpine individuals implying that the result from DATES could be biased as the alpine individuals from CA-EBA might not be the best source for SIU01.

## Text S8. Approach used in the analyses to avoid possible bias using the Daicel Arbor Biosciences capture kit

For capture analysis, we used (Human Affinities, Version 1.0, March 2021 - Daicel Arbor Bioscience) which includes three different target sets: 1) Prime 1240K with approx. 1.24 million population-informative SNPs, 2) Y Chromosome 46K targets sites on the Y Chromosome, 3) MitoTrio probes which cover the complete mtDNA genome.

Recently, two studies[101,102] have shown that capture data obtained by using this commercial kit, may present allelic read coverage bias at a portion of targeted sites and showed limitations in cross-compatibility of this kit with other assays, shotgun sequencing or other target enrichment panels such as “Twist Ancient DNA” (Twist Bioscience) and 1240K assay[85].  In practice, co-analysis of data produced with different panels could lead to the observation of artefactual genetic similarities among samples analysed with the same Arbor kit. This has been found to potentially affect the estimation of genetic affinity, especially in the context of f-statistics (including $f_3$-statistics and $f_4$-statistics), among samples and populations. Even if not tested, the authors also hypothesized that qpAdm and qpWave analyses might be biased as well.

In general, as a first step in our analysis, we ensured that no individuals in the AADR dataset used for comparisons had been analysed with the same Arbor kit. Furthermore, with regard to our alpine dataset which includes samples analysed with the same kit, we expect that any bias would affect all samples equally. One exception is the Iceman since the genomic data for this individual have been produced using a different approach (high coverage genome[86] , see below).

As additional precautionary measures, we strictly followed the recommendations reported by Davidson and collaborators[102].

For all *f-statistics* performed in this study, the alpine target individual/population used for the analyses was the only one enriched with the Arbor kit (e.g. prehistoric alpine samples) whereas the outgroup and individuals/populations used for comparison were enriched using different approaches avoiding possible distortion in genomic affinities. Even for the Iceman, no distortion should be expected when running the f-statistic on this individual alone, as it does not include Arbor's data. The only exception could be the qpAdm analysis, performed using as a source for WHGs the local alpine ME individuals (MAD01), enriched with the same Arbor kit. In order to check if our results obtained using the local ME, could have affected our analyses, an additional model was performed using an alternative ME individual (*Drigge* DRI, from Germany and dating from 7,412-6,947 BP) enriched with a different approach[103,104]. This sample was chosen as it has a high affinity to MAD01 as visible in the MDS plot (Fig. 2B). Moreover, these two ME individuals share similar admixture proportions of WHGs and EHGs related-ancestry (*Oberkassel,* WHGs = 87.4 ± 2.5% and EHGs = 12.6 ± 2.5%). Results show that for a model with DRI and early NE farmers from Anatolian used as sources, similar proportions of admixture were found compared to the model using MAD01 (DRI from 9.5 ± 2.1% to 20.3 ± 2.1% and Anatolian farmers from 79.7 ± 2.3% to 90.5 ± 2.3%) (Supplementary Data 17). These results indicate that our qpAdm analysis is not affected by genetic bias and confirm the authenticity of our findings. Additionally, if we consider the Iceman, the results obtained in our study from qpAdm analysis for this sample are very similar to those that have been found by Wang and collaborators[86] confirming the authenticity of our results (see Results section).

Consequently, we consider MAD01 as a source in the qpAdm models as this ME sample came from the same geographical region as other prehistoric individuals of this study.

Regarding the other analyses performed in this study (e.g. kinship analyses and PCA), the use of the genomic data produced by the Arbor kit could result in very little or no bias. In fact, it has been found[102] that kinship analysis performed using the READ method, based on the

mismatch rate between pairs of individuals and applied in this study, is only slightly affected when analyzing data produced using different kits/methods. Other kinship methods were not tested by Davidson and colleagues. However, we expect that the use of our capture data in the analyses using the other two methods (TKGWV2 and KIN) that use different approaches than READ (see Methods), will not significantly affect our results. This is especially true for the KIN method as it is based on identical by-descent segments (IBD) inherited by a common ancestor. Moreover, the two methods produced similar results as READ.

Additionally, in our study, only capture data obtained using exclusively the Arbor kit were used for kinship analyses, except for two individuals (STE01 and STE03) for which only shotgun data are available. However, these samples were excluded from downstream analyses due to uncertainty of their kinships (see Text S4 and Supplementary Data 5). Moreover, it should be noted that our results on kinship analyses were performed using three different approaches and only kinships confirmed by at least two methods were considered. These results based on autosomal data were also confirmed by Y-Chromosome and mtDNA data which further support relatedness along the paternal or maternal lines and are supported by archaeological information since all kinships were detected among individuals recovered from the same archaeological site.

Finally, as far as PCA analyses are concerned, we are confident that our results were genuine since the studies from[101,102], clearly showed that this analysis is not affected by any bias. In fact, the plots performed projecting the ancient samples, generated by the Arbor kit or using different assays (Twist Bioscience, 1240K array, shotgun), on the PCA constructed by using data from present-day populations provided almost identical results.

## Text S9. Analysis of phenotypic SNPs

Phenotypic traits such as hair, eye and skin color in the alpine prehistoric alpine individuals were predicted by using the Hirisplex panel which consist of 41 SNPs (https://hirisplex.erasmusmc.nl/, see Supplementary Data 21 for details) from 19 genes. Full prediction (for all three phenotypes together) could be done only for six individuals including three CA (SOL02, COR01 and NOG201), two CA-EBA (COR02 and ROM402) and one EBA sample (ROM305), which all likely had brown eye-color associated with dark-brown to black hair colour, similar to what have been found for the Iceman[86,105]. It was possible to predict the skin colour in 19 individuals from almost all chronologies (three MN, seven CA, eight CA-EBA and one EBA). These included 18 samples, with a possible pale to dark intermediate skin colour, and one individual (MEZ03) from CA who was predicted to have dark to black skin

colour (Supplementary Data 21). Differently from the results found by[86], we predicted an intermediate rather than a darker skin colour for the Iceman in our study. This incongruence might be due to the smaller number of SNPs analysed in our study compared to Wang (41 SNPs vs. 170 SNPs associated with skin-pigmentation). Moreover, Wang et al.[86] calculated a polygenetic score to predict skin colour whereas Hirisplex gives prediction for only four categories (pale, intermediate, dark and dark-to-black skin colour). For these reasons, we considered our results on these complex traits with great caution.

In addition, we attempt to analyze the allele frequencies of 72 SNPs, which have been investigated by Wang and collaborators[86], related to phenotypic traits such as diabetes, metabolic disorders, male-pattern baldness, lactase persistence, agriculturalist's diet and adaptation to high altitude (see Supplementary Data 22 for details). The allele frequencies were calculated in the alpine individuals grouped according to chronology (ME, MN, CA, CA-EBA and EBA) but also grouped based on different ancestral models and period (Fig. S24). However, due to missing data, the allele frequency could not be calculated for some SNPs as well as in some groups (Supplementary Data 22). The frequency of some interesting phenotypic SNPs, such as the derived allele associated with lactose tolerance (MCM6/rs4988235), could be determined for all groups. This allele was found to be absent in all prehistoric alpine individuals from ME to MBA.

Additionally, two SNPs (e.g. rs1495741 and rs4751995) which have been associated with the adaptation to agricultural lifestyle and diet were successfully analysed in this study. The derived allele of SNP rs1495741 in the NAT2 gene is associated with slower acetylation which has been hypothesized to be advantageous in agricultural populations[106–108] .It has been shown that the Iceman is a slow metaboliser with high concentrations of plant-oriented fatty acids[86], which is also the case in the other CA analysed individuals. Additionally, our study showed the same result for all the other ancient alpine groups from the MN to MBA, including the ME individual, supporting previous findings that showed no change in the allele frequency of the derived allele of SNP rs1495741 over the last 10,000 years[109] .

Moreover, the SNP rs4751995 of the gene PLRP2 has been associated with a high concentration of fatty acids of vegetable origin and therefore probably a better ability to digest plants, but also with the consumption of cereals in the diet[110]. The alpine ME sample has the reference allele at this position while the derived allele is observed in the alpine individuals from MN group onwards. The presence of the derived allele increases from MN to CA-EBA (from 62.5% to 75%) with a decline in the following CA-EBA period (50%) while the alpine

groups from EBA show only this allele (Supplementary Data 22). In general, these results may suggest adaptation in EIAlp to a farmers' diet.

Furthermore, the presence of allele related to reduce hair curliness, as has been found in the Iceman[86], was observed in all time periods. Finally, several variants on the EPAS1 gene associated with the hemoglobin level and which has been related to high altitude adaptation in Tibet (e.g.)[111], were also successfully analysed in this study. None of these variants, except for one (rs116062164), were observed in our alpine individuals, likely indicating no adaptation of alpine prehistoric groups to high altitude.

**References**

1. Corrain, C., Graziati, G. & Leonardi, P. La sepoltura epipaleolitica nel riparo di Vatte di Zambana (Trento). *Preistoria Alpina* 12, 175–212 (1976).

2. Sparacello, V. S. *et al.* A history of violence in the Mesolithic female skeleton from Mezzocorona-Borgonuovo (Trento, northeastern Italy). *Quat Sci Rev* 311, 108149 (2023).

3. Fontana, F. *et al.* A snapshot of late mesolithic life through death: An appraisal of the lithic and osseous grave goods from the Castelnovian burial of Mondeval de Sora (Dolomites, Italy). *PLoS One* 15, (2020).

4. Hodgkins, J. *et al.* An infant burial from Arma Veirana in northwestern Italy provides insights into funerary practices and female personhood in early Mesolithic Europe. *Sci Rep* 11, (2021).

5. Dalmeri, G. & Pedrotti, A. Distribuzione topografica dei siti del Paleolitico Superiore finale e Mesolitico in Trentina Alto-Adige e nelle Dolomiti Venete (Italia). Topographic distribution of Late Upper Palaeolithic and Mesolothic sites in Trentino Alto-Adige region and in the Venetian Dolomites (ltaly). *Preistoria Alpina* 28, 247–267 (1994).

6. Bagolini, B. & Grifoni Cremonesi, R. Il Neolitico italiano: facies culturali e manifestazioni funerarie. *Bullettino di Paletnologia Italiana* 85, 139–170 (1994).

7. Bernabò Brea, M. & Mazzieri, P. Osservazione sulla sfera rituale del mondo VBQ in base ai dati forniti dagli insediamenti dell'Emilia occidentale. in *Il pieno sviluppo del Neolitico in Italia, Museo Archeologico del Finale – Finale Ligure Borgo 8–10 giugno 2009* (eds. Bernabò Brea, M., Maggi, R. & Manfredini, A.) 315–321 (Rivista di Studi Liguri, 2014).

8. Mottes, E. & Nicolis, F. Forme della ritualità funeraria tra età del Rame e antica età del Bronzo nel territorio della Valle dell'Adige (Trentino Alto Adige, Italia settentrionale). in *Un lungo percorso di scienza. Scritti in onore di Leone Fasani, Memorie del Museo Civico di Storia Naturale di Verona – 2. serie - Sezione Scienze dell'Uomo – 13, Millenni. Studi di Archeologia Preistorica 22* (eds. Martini, F. & Salzani, L.) vol. 22 191–219 (2019).

9. Barfield, L. H. Burials and boundaries in chalcolithic Italy. in *Papers in Italian Archaeology IV. The Cambridge Conference. Part II: Prehistory* (eds. Malone, C. & Stoddart, S.) 152–176 (BAR Publishing, Oxford, 1985).

10. Nicolis, F. Le evidenze funerarie dell'antica età del Bronzo in Italia settentrionale. in *Graves and Funerary Rituals during the Late Neolithic and the Early Bronze Age in Europe (2700-2000 BC)* (eds. Besse, M. & Desideri, J.) 111–145 (BAR International Series 1284, 2004).

11. Nicolis, F. *Some Observations on the Cultural Setting of the Bell Beakers of Northern Italy (Proceedings of the International Colloquium, Riva Del Garda, 11-16 May 1998).* vol. II (Trento, 2001).

12. Battisti, M. & Tecchiati, U. *The Archaeological Excavations in the Castel Corno Caves (Isera, Trento, Italy). Burial Places and Settlement of a Small Alpine Community between the 25th and 17th Centuries BC.* (Archaeopress, 2022).

13. Avanzini, M. *et al.* Bersaglio di Mori, dati e ricerche. *Annali del Museo Civico di Rovereto* 1, 23–66 (1985).

14. Avanzini, M. *et al.* Bersaglio di Mori, dati e ricerche. *Annali del Museo Civico di Rovereto* 1, 23–66 (1985).

15. Mazzucchi, A., Bonelli, G., Battisti, M. & Tecchiati, U. Le sepolture preistoriche delle Grotte di Castelcorno di Isera (TN). *Annali del Museo Civico di Rovereto* 35, 3–31 (2020).

16. Barfield, L. H. L'insediamento neolitico "ai Corsi" presso Isera (Trento). *Studi Trentini di Scienze Naturali* XLVII, 56–77 (1970).

17. Pedrotti, A. Il Neolitico. in *Storia del Trentino, I, Preistoria e Protostoria* (eds. Lanzinger, M., Marzatico, F. & Pedrotti, A.) 119–182 (Il Mulino, Bologna, 2001).

18. Angelini, B., Artioli, G., Pedrotti, A. & Tecchiati, U. La metallurgia dell'età del Rame dell'Italia settentrionale con particolare riferimento al Trentino e all'Alto Adige. Le risorse minerarie e i processi di produzione del metallo. in *L'età del Rame: la pianura padana e le Alpi al tempo di Ötzi* (ed. De Marinis, R. C.) 101–116 (Compagnia della stampa Massetti Rodella, Brescia, 2013).

19. Visentini, P. La fine del Neolitico nell'Italia nord-orientale insediamenti e produzioni tra V e IV millennio a.C. in *Millenni, Studi di archeologia preistorica* vol. 15 (Museo e Istituto Fiorentino di Preistoria 'Paolo Graziosi', Firenze, 2018).

20. Mottes, E., Petrucci, G., Rottoli, M. & Visentini, P. Evolution of the Square Mouthed Pottery Culture in Trentino-Alto Adige, Veneto and Friuli: Cultural, Chronological, Palaeoeconomic and Environmental aspects. *Gortania - Geologia, Paleontologia, Paletnologia* 31, 97–124 (2010).

21. De Marinis, R. C. & Pedrotti, A. L'età del Rame nel versante italiano delle Alpi centro-occidentali. in *La Valle d'Aosta nel quadro della preistoria e protostoria dell'arco alpino centro-occidentale. Atti della XXXI Riunione Scientifica. Courmayeur, 2-5 giugno 1994* 247–300 (Istituto Italiano di Preistoria e Protostoria, Firenze, 1997).

22. Pedrotti, A. Il Neolitico. in *Storia del Trentino. La preistoria e la protostoria* (eds. Lanzinger, M., Marzatico, F. & Pedrotti, A.) vol. 1 119–181 (Il Mulino, Bologna, 2001).

23. De Marinis, R. C. La necropoli di Remedello Sotto e l'età del Rame nella pianura padana a nord del Po. in *L'età del Rame: la pianura padana e le Alpi al tempo di Ötzi.* (ed. De Marinis, R. C.) 301–351 (Compagnia della stampa Massetti Rodella, Brescia, 2013).

24. Barfield, L. H. La stazione neolitica de "La Vela" presso Trento. Considerazioni sulle tombe a cista nel Trentino Alto Adige. *Studi Trentini di Scienze Naturali* XLVII, 35–55 (1970).

25. Mottes, E. *Le Spirali Del Tempo, Meandri Del Passato. Gli Scavi Archeologici a La Vela Di Trento Dal 1960 al 2007*. (Provincia autonoma di Trento, Soprintendenza per i Beni Archeologici, Trento, 2007).

26. Mottes, E. *Vasi a Bocca Quadrata. Evoluzione Delle Conoscenze, Nuovi Approcci Interpretativi*. (Provincia autonoma di Trento, Soprintendenza per i beni culturali, 2021).

27. Pedrotti, A. L'abitato neolitico de «La Vela» di Trento. in *Die ersten Bauern. Pfahlbaufunde Europas* vol. 2 219–224 (Schweizerisches Landesmuseum Zurich, Zürich, 1990).

28. Corrain, C. & Capitanio, M. I resti scheletrici umani della necropoli neolitica di 'La Vela' (Trento). *Preistoria Alpina* 30, 5–42 (1996).

29. Pedrotti, A. La pietra levigata nei corredi delle sepolture neolitiche dell'Italia settentrionale. in *Le vie della pietra verde. L'industria litica levigata nella preistoria dell'Italia settentrionale* (ed. Venturino Gambari, V.) 150–164 (Omega, Torino, 1996).

30. Mazzieri, P., Colombo, M., Bernabò Brea, M. & Grifoni Cremonesi, R. Contatti e scambi tra la cultura Serra d'Alto e i vasi a Bocca Quadrata: il caso delle ollette tipo San Martino. in *Xarxes al Neolític. Actes Congrés Internacional, Gavá- Bellaterra, 2-4.02.2011* 351–361 (Rubricatum: revista del Museu de Gavà, Gavá, Bellaterra, 2012).

31. Fasani, L. La sepoltura e il forno di fusione de La Vela di Valbusa (Trento). *Preistoria Alpina* 24, 165–181 (1990).

32. Corrain, C. Il profilo antropologico dell'inumato di Vela (Trento), della prima età del bronzo. *Preistoria Alpina* 7, 227–278 (1971).

33. Corrain, C. Il profilo antropologico dell'inumato di Vela (Trento), della prima età del bronzo. *Preistoria Alpina* 7, 227–278 (1971).

34. Tecchiati, U. Il riparo del santuario in 'Val Cornelio' (Comune di Lasino-Trentino): una successione stratigrafica dall'Eneolitico recente al Bronzo finale. (University of Trento, 1991).

35. Bonardi, A. & Tecchiati, U. Risultati delle ricerche 1994 e 1996 nel sito dell'età del Bronzo del Riparo del Santuario in Val di Cavedine (TN). *Annali del Museo Civico di Rovereto* 20, 3–21 (2005).

36. Tecchiati, U. Alcune considerazioni sull'ultima fase di frequentazione del Riparo del Santuario di Lasino in Val di Cavedine (Bronzo recente e finale: XIII-X sec. a.C.). *Judicaria* 86, 77–88 (2014).

37. Chiusole, P. & Bergamo Decarli, G. B. Sondaggio al riparo del 'Santuario' in 'Val Cornelio' nel Comune di Lasino (Trentino). *Pubblicazione della Società Museo Civico di Rovereto* LXXIV, (1969).

38. Chiusole, P. & Vettori, S. Sondaggio stratigrafico al riparo del "Santuario" in "Val Cornelio" nel comune di Lasino (Trentino). *Pubblicazione della Società Museo Civico di Rovereto* LXXVI, (1972).

39. Tecchiati, U. Sepolture e resti umani sparsi in abitati della preistoria e della protostoria dell'Italia settentrionale con particolare riferimento al Trentino-Alto Adige. *Notizie Archeologiche Bergomensi* 3, 1–18 (2011).

40. Riedel, A. & Tecchiati, U. La fauna del Riparo del Santuario (Comune di Lasino – Trentino): aspetti archeozoologici, paleoeconomici e rituali. *Annali dei Musei Civici di Rovereto, Sezione Archeologia, Storia, Scienze Naturali* 8, 3–46 (1992).

41. Angelini, B., Pasquali, T. & Pedrotti, A. Madonna Bianca Rockshelter (Trento). *Preistoria Alpina* 33, 229–231 (2001).

42. Dalmeri, G. & Nicolodi, F. Siti e collezioni antropologiche, preistoriche e protostoriche provinciali del Museo Tridentino di Scienze Naturali (verifica di archivio riferita al 1998). *Preistoria Alpina* 40, 63–81 (2005).

43. Dalmeri, G., Mottes, E. & Nicolis, F. The Mesolithic burial of Mezzocorona-Borgonuovo (Trento): some preliminary comments. *Preistoria Alpina* 40 (2004), 63–82 (2005).

44. Bagolini, B. *et al.* Il riparo di Moletta Patone di Arco nel Trentina meridionale. *Preistoria Alpina* 20, 103–146 (1984).

45. Corrain, C., Bettini, V., Mayellaro, F., Martino, R. & Velussi, C. Caratteristiche dentarie degli inumati di Moletta Patone. *Preistoria Alpina* 21, 85–89 (1985).

46. Barfield, L. H. *Excavations in the Riparo Valtenesi, Manerba, 1976-1994.* (ORIGINES, 2007).

47. Ferembach, D., Schwidetzky, I. & Stloukal, M. Empfehlungen für die Alters- und Geschlechtsdiagnose am Skelett. *Homo* 30, 1–32 (1979).

48. Rösing, F. W. Methoden und Aussagemöglichkeiten der anthropologischen Leichenbrandbearbeitung. *Archäologie und Naturwissenschaften* 1, 53–80 (1977).

49. Bagolini, B. & Biagi, P. Balkan influences in the Neolithic of Northern Italy. *Preistoria Alpina* 21, 49–57 (1985).

50. Nicolis, F. Il culto dei morti nell'antica e media età del Bronzo. in *Storia del Trentino. La preistoria e la protostoria* (eds. Lanzinger, M., Marzatico, F. & Pedrotti, A.) vol. 1 337–365 (Il Mulino, Bologna, 2001).

51. Mottes, E., Degasperi, N., Gaudio, D. & Zana, M. Nuova sepoltura dell'età del Rame a Nogarole di Mezzolombardo (Trento) in Valle dell'Adige. *Archeologia delle Alpi 2017-2019* 11–19 (2019).

52. Bagolini, B. *Introduzione al Neolitico Dell'Italia Settentrionale, Introduzione Alla Ricerca Preistorica.3*. (Pordenone, 1980).

53. Corrain, C. & Erspamer, G. Gli scheletri tardo-neolitici giovanili di Volano presso Rovereto (Trento). *Preistori Alpina- Museo Tridentino di Scienze Naturali* 14, 197–203 (1978).

54. Reimer, P. J. *et al.* The IntCal20 Northern Hemisphere Radiocarbon Age Calibration Curve (0–55 cal kBP). *Radiocarbon* 62, 725–757 (2020).

55. Perini, R. Notizie sulle scoperte preistoriche al Loc di Romagnano (Trento). *Natura Alpina* XX, 65–69 (1969).

56. Perini, R. I depositi preistorici di Romagnano - Loc (Trento). *Preistoria Alpina* 7, 7–106 (1971).

57. Broglio, A. Risultati preliminari delle ricerche sui complessi epipaleolitici della Valle dell'Adige. *Preistoria Alpina (Rendiconti)* 7, 135–241 (1971).

58. Fontana, F., Flor, E. & Duches, R. Technological continuity and discontinuity in the Romagnano Loc III rock shelter (NE Italy) Mesolithic series. *Quaternary International* 423, 252–265 (2016).

59. Bagolini, B. & Biagi, P. Le più antiche facies ceramiche dell'ambiente Padano. *Rivista di Scienze Preistoriche* 32, 219–233 (1977).

60. Perini, R. La necropoli di Romagnano-Loc III e IV. Le tombe all'inizio dell'età del bronzo nella regione Sudalpina Centroorientale. *Preistoria Alpina* 11, 295–315 (1975).

61. Capitanio, M. I resti scheletrici umani, riferibili agli inizi dell'Età del Bronzo, finora rinvenuti a Loc di Romagnano (Trento). *Preistoria Alpina* 9, 7–43 (1973).

62. Marzatico, F. & Tecchiati, U. *L'età Del Bronzo in Trentino e Alto Adige-Südtirol. Atti della XXXIII riunione scientifica. Preistoria e protostoria del Trentino Alto Adige/Südtirol. Trento, 21-24 ottobre 1997* (Istituto Italiano di Preistoria e Protostoria, Trento, 2002).

63. Perini, R. *L'età Del Bronzo Antico e Medio*. (Il Mulino, Bologna, 2001).

64. Mottes, E. L'insediamento e la necropoli dell'antica età del Bronzo dei Solteri di Trento. in *L'Antica età del bronzo, Atti del Congresso di Viareggio (9-12.01.1995)* (ed. Cocchi Genick, D.) 542–543 (OCTAVER, Firenze, 1996).

65. Corrain, C. & Capitanio, M. I resti scheletrici umani provenienti dalle stazioni trentine del Neo-eneolitico e dell'Età del Bronzo. *Studi Trentini di Scienze Naturali, Sez.B.* 44, 135–250 (1967).

66. Perini, R. *Sulle Tracce Delle Antiche Genti Giudicariesi*. (Provincia Autonoma di Trento, Trento, 1983).

67. Perini, R., Corrain, C. & Capitanio, M. Le necropoli a tumulo di Stenico - Calferi (Trento): notizie archeologiche e studio antropologico. *Archivio per l'Antropologia e la Etnologia* 121, 45–49 (1991).

68. Perini, R. L'età del Bronzo Antico e Medio. in *Storia del Trentino. La preistoria e la protostoria* (eds. Lanzinger, M., Marzatico, F. & Pedrotti, A.) vol. 1 287–355 (Il Mulino, Bologna, 2001).

69. Bassetti, M., Degasperi, N. & Nicolis, F. Volano prima della storia. in *Volano. Storia di una comunità* (eds. Adami, R., Bonazza, M. & Varanini, G. M.) 27–57 (Nicolodi Editore, Rovereto, 2005).

70. Tecchiati, U. Luoghi di culto, sepolture e sepolcreti dell'età del Rame dell'area atesina. in *L'età del Rame: la pianura padana e le Alpi al tempo di Ötzi* (ed. De Marinis, R. C.) 457–480 (Compagnia della stampa Massetti Rodella, Brescia, 2013).

71. Rizzi, J., Conzato, A. & Marzoli, C. Anthropological and taphonomic investigation of a Copper Age multiple burial from South Tyrol, Italy. *Notizie Archeologiche Bergomensi* 18, 33–45 (2010).

72. Paladin, A. *et al.* Archaeological questions and genetic answers: Male paternal kinship in a copper age multiple burial from the eastern Italian Alps. *J Archaeol Sci Rep* 50, 104103 (2023).

73. Salzani, P. & Tecchiati, U. Circolazione dei materiali e delle materie prime e loro contributo allo sviluppo e alla diffusione di elementi legati alla sfera dell'ideologia e della spiritualità tra il IV e III Millennio BC: area alpina e area padana centro orientale a confronto. in *Le quistioni nostre paletnologiche più importanti. Trent'anni di tutela e ricerca preistorica in Emilia occidentale. Atti del Convegno di Studi in onore di Maria Bernabò Brea. Parma, Palazzo della Pilotta, 8-9 giugno 2017* (eds. Maffi, M., Bronzoni, L. & Mazzieri, P.) 193–212 (2019).

74. Steiner, H. Schlanders, 'Talele'. in *Tutela dei Beni Culturali. Ufficio Beni Archeologici Provincia Autonoma di Bolzano - Alto Adige* 194–196 (2009).

75. Steiner, H., Zink, A. & Tecchiati, U. Frühbronzezeitliche Hockerbestattung aus Schlanders. *Der Schlern* 91, 14–29 (2017).

76. De Marinis, R. C. Riti funerari e problemi di paleo-demografia dell'antica etÀ del Bronzo nell'Italia settentrionale. *Notizie Archeologiche Bergomensi* 11, 5–78 (2003).

77. Tecchiati, U. Alle soglie dell'età del Rame. Il luogo di culto di Varna-Circonvallazione (Bz). in *Studi in memoria di Angelo Rampinelli Rota. Atti del Convegno Le manifestazioni del sacro e l'età del rame nella regione alpina e nella Pianura Padana. Brescia, Palazzo Broletto, 23-24 maggio 2014* (ed. De Marinis, R. C.) 85–110 (Compagnia della stampa Massetti Rodella, Brescia, 2014).

78. Dal Ri, L., Rizzi, G. & Tecchiati, U. L'area megalitica dell'etá del Rame Velturno- loc. Tanzgasse (BZ). Aggiornamento sullo stato delle ricerche. in *L'area funeraria e cultuale dell'etá del Rame di Sovizzo nel contesto archeologico dell'Italia settentrionale. Quaderni di Archeologia Vicentina* (ed. Bianchin Citton, E.) vol. 1 125–174 (Museo Naturalistico Archeologico - Vicenza, Vicenza, 2004).

79. Dal Ri, L., Rizzi, G. & Tecchiati, U. *Una Necropoli Preistorica a Barbiano in Valle d'Isarco (Bolzano)*. (2002).

80. Tecchiati, U. Sotciastel. Nascita e abbandono di un villaggio fortificato dell'età del Bronzo e sue relazioni con il popolamento della macroregione padano-alpina. *Ladinia* 44, 15–52 (2020).

81. Tecchiati, U. Principali risultati delle ricerche sul villaggio fortificato di Sotćiastel (Val Badia, BZ) e alcuni problemi dell'età del bronzo dell'alto bacino dell'Adige. *Ladinia* 22, 13–61 (1998).

82. Tecchiati, U. Sepolture e resti umani sparsi in abitati della preistoria e della protostoria dell'Italia settentrionale con particolare riferimento al Trentino-Alto Adige. *Notizie Archeologiche Bergomensi* 19, 49–63 (2011).

83. Nadeau, M.-J. *et al.* Dating the finds contained in the cenotaph of Queen Editha. in *Königin Editha und ihre Grablegen in Magdeburg* (eds. Melle, H., Schenkluhn, W. & Schmuhl, B.) vol. 18 (Landesamt für Denkmalpflege und Archäologie Sachsen-Anhalt - Landesmuseum für Vorgeschichte, 2012).

84. Reimer, P. J. *et al.* The IntCal20 Northern Hemisphere Radiocarbon Age Calibration Curve (0–55 cal kBP). *Radiocarbon* 62, 725–757 (2020).

85. Mathieson, I. *et al.* Genome-wide patterns of selection in 230 ancient Eurasians. *Nature* 528, 499–503 (2015).

86. Wang, K. *et al.* High-coverage genome of the Tyrolean Iceman reveals unusually high Anatolian farmer ancestry. *Cell Genomics* 3, (2023).

87. Kuhn, J. M. M., Jakobsson, M. & Günther, T. Estimating genetic kin relationships in prehistoric populations. *PLoS One* 13, (2018).

88. Fernandes, D. M., Cheronet, O., Gelabert, P. & Pinhasi, R. TKGWV2: an ancient DNA relatedness pipeline for ultra-low coverage whole genome shotgun data. *Sci Rep* 11, (2021).

89. Popli, D., Peyrégne, S. & Peter, B. M. KIN: a method to infer relatedness from low-coverage ancient DNA. *Genome Biol* 24, (2023).

90. Ringbauer, H., Novembre, J. & Steinrücken, M. Parental relatedness through time revealed by runs of homozygosity in ancient DNA. *Nat Commun* 12, (2021).

91. Guilaine, J. The Neolithization of Mediterranean Europe: Mobility and interactions from the Near East to the Iberian peninsula. in *The Oxford Handbook of Neolithic Europe* (eds. Fowler, C., Harding, J. & Hofmann, D.) 81–98 (Oxford University Press, Oxford, 2015).

92. Barnett, W. K. Cardial pottery and the agricultural transition in Mediterranean Europe. in *Europe's First Farmers* (ed. Douglas Price, T.) 93–116 (Cambridge University Press, 2000).

93. Rivollat, M. *et al.* Ancient genome-wide DNA from France highlights the complexity of interactions between Mesolithic hunter-gatherers and Neolithic farmers. *Science Advance* 6, eaaz5344 (2020).

94. Olalde, I. *et al.* A common genetic origin for early farmers from mediterranean cardial and central european LBK cultures. *Mol Biol Evol* 32, 3132–3142 (2015).

95. Mathieson, I. *et al.* The genomic history of southeastern Europe. *Nature* 555, 197–203 (2018).

96. Haak, W. *et al.* Massive migration from the steppe was a source for Indo-European languages in Europe. *Nature* 522, 207–211 (2015).

97. Chintalapati, M., Patterson, N. & Moorjani, P. The spatiotemporal patterns of major human admixture events during the European Holocene. *Elife* 11, (2022).

98. Posth, C. *et al.* Palaeogenomics of Upper Palaeolithic to Neolithic European hunter-gatherers. *Nature* 615, 117–126 (2023).

99. Yu, H. *et al.* Genomic and dietary discontinuities during the Mesolithic and Neolithic in Sicily. *iScience* 25, 104244 (2022).

100. Wang, K. *et al.* High-coverage genome of the Tyrolean Iceman reveals unusually high Anatolian farmer ancestry. *Cell Genomics* 3, 100377 (2023).

101. Rohland, N. *et al.* Three assays for in-solution enrichment of ancient human DNA at more than a million SNPs. *Genome Res* 32, 2068–2078 (2022).

102. Davidson, R. *et al.* Allelic bias when performing in-solution enrichment of ancient human DNA. *Mol Ecol Resour* 23, 1823–1840 (2023).

103. Fu, Q. *et al.* The genetic history of Ice Age Europe. *Nature* 534, 200–205 (2016).

104. Posth, C. *et al.* Palaeogenomics of Upper Palaeolithic to Neolithic European hunter-gatherers. *Nature* 615, 117–126 (2023).

105. Keller, A. *et al.* New insights into the Tyrolean Iceman's origin and phenotype as inferred by whole-genome sequencing. *Nat Commun* 3, (2012).

106. Luca, F. *et al.* Multiple advantageous amino acid variants in the NAT2 gene in human populations. *PLoS One* 3, e3136 (2008).

107. Sabbagh, A., Darlu, P., Crouau-Roy, B. & Poloni, E. S. Arylamine N-Acetyltransferase 2 (NAT2) Genetic Diversity and Traditional Subsistence: A Worldwide Population Survey. *PLoS One* 6, e18507 (2011).

108. Magalon, H. *et al.* Population genetic diversity of the NAT2 gene supports a role of acetylation in human adaptation to farming in Central Asia. *European Journal of Human Genetics* 16, 243–251 (2008).

109. Mathieson, S. & Mathieson, I. FADS1 and the timing of human adaptation to agriculture. *Mol Biol Evol* 35, 2957–2970 (2018).

110. Hancock, A. M. *et al.* Human adaptations to diet, subsistence, and ecoregion are due to subtle shifts in allele frequency. *Proc Natl Acad Sci U S A* 107, 8924–8930 (2010).

111. Bhandari, S. *et al.* Genetic evidence of a recent Tibetan ancestry to Sherpas in the Himalayan region. *Sci Rep* 5, 16249 (2015).

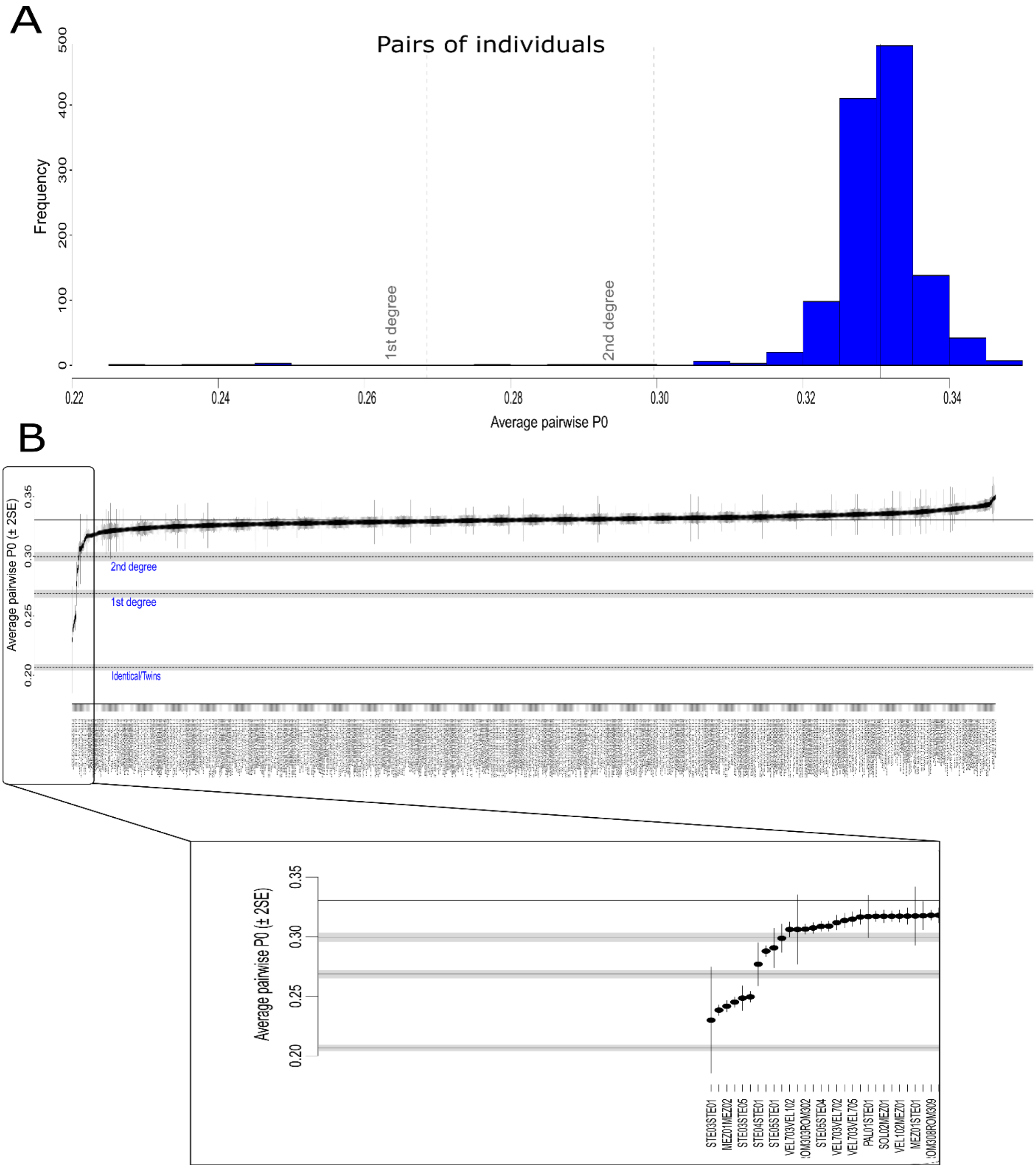


**Figure S17. Kinships estimated by READ.** (A) Histogram of the non-normalized average *P0* values. The solid vertical line indicates the median value used for normalization and the dashed lines show the cutoffs for the different levels of relatedness. (B) Pairwise comparisons values of the non-normalized average *P0* between all the individuals. Error bars show the standard errors of the mean, and the horizontal lines indicate the same as in (A).

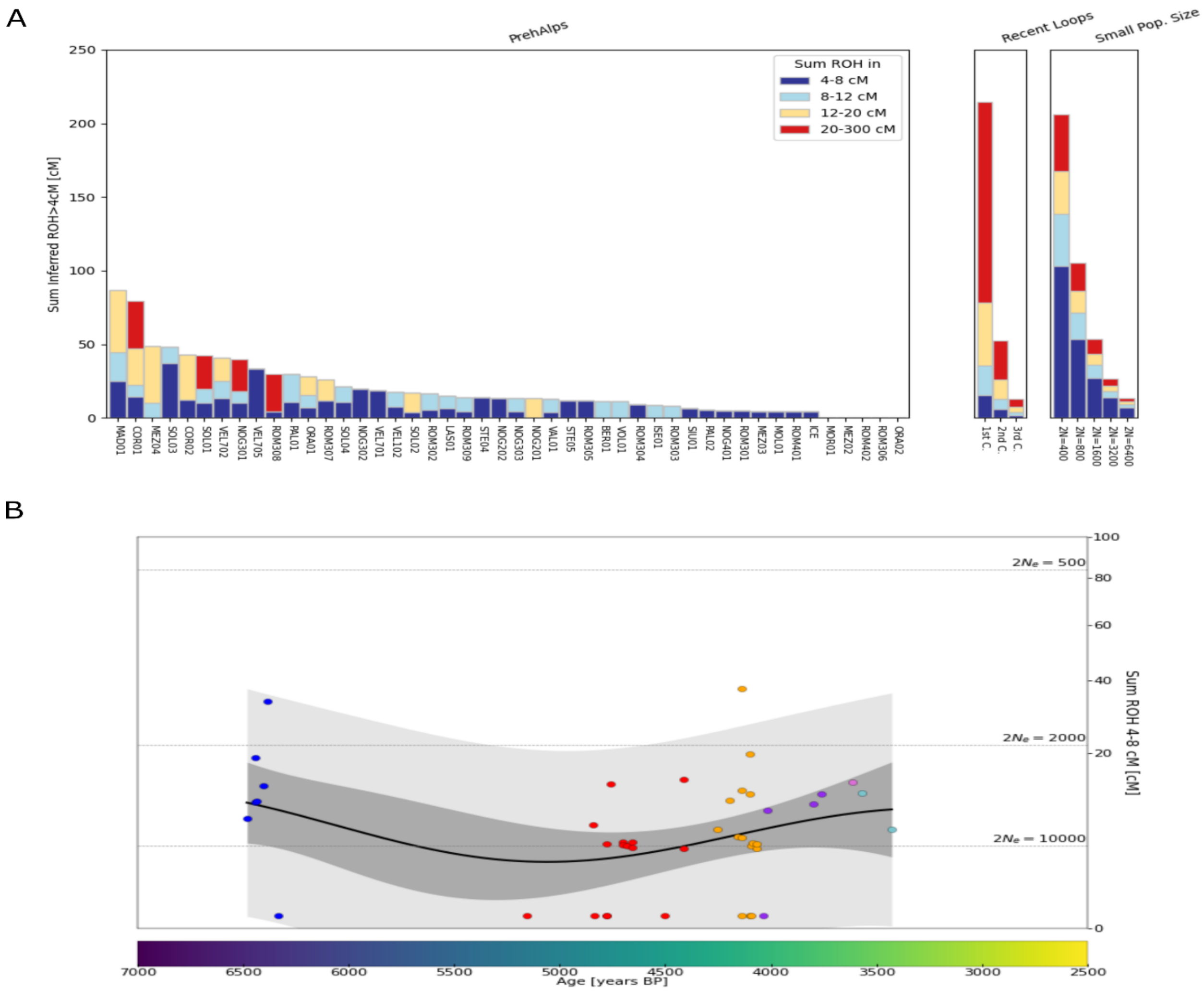


**Figure S18**. **Run of Homozygosity (ROH) values**. **(A)** Each vertical bar represents an individual and the length of each colored bar is determined by the sum of ROH falling into four different classes (4-8, 8-12, 12-20 and >20 cM). On the left is represented the results for the prehistoric alpine individuals and on the right is shown expected ROH for different levels of offspring of close kin (first, second or third cousins) or population size (*Ne*), based on analytical calculations[84]. **(B)** Plot of the sum of ROH of length between 4-8 cM ($sROH_{[4-8]}$) for each individual. The mean estimate (black line), the 95% empirical confidence intervals for both individuals (light gray) and the estimated mean (dark gray) were calculated from a Gaussian Process model. The horizontal dashed lines indicate the expected panmictic population sizes for the corresponding $sROH_{[4-8]}$. The colored dots represent each individual by time period (blue = middle Neolithic, red = Copper Age, orange = Copper Age - Early Bronze Age, purple = Early Bronze Age, pink = Early / Middle Bronze Age and light blue = Middle Bronze Age).

A.

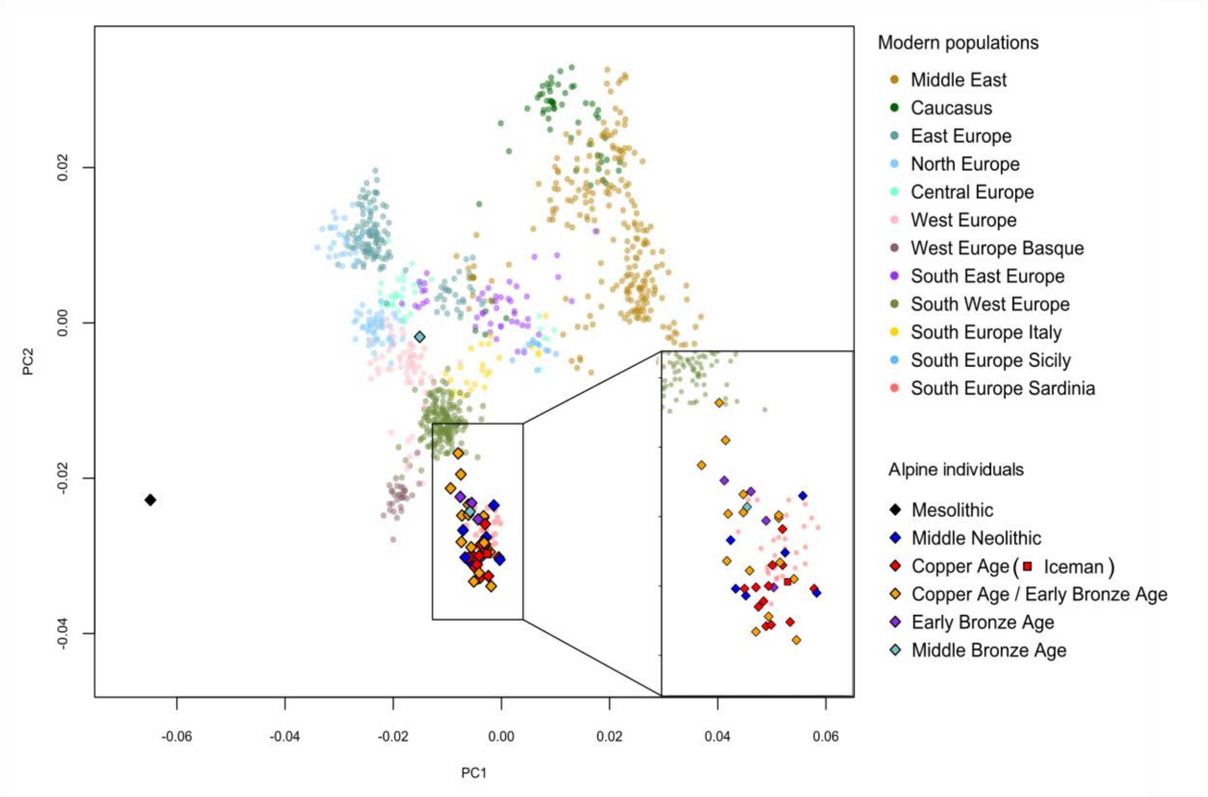


B.

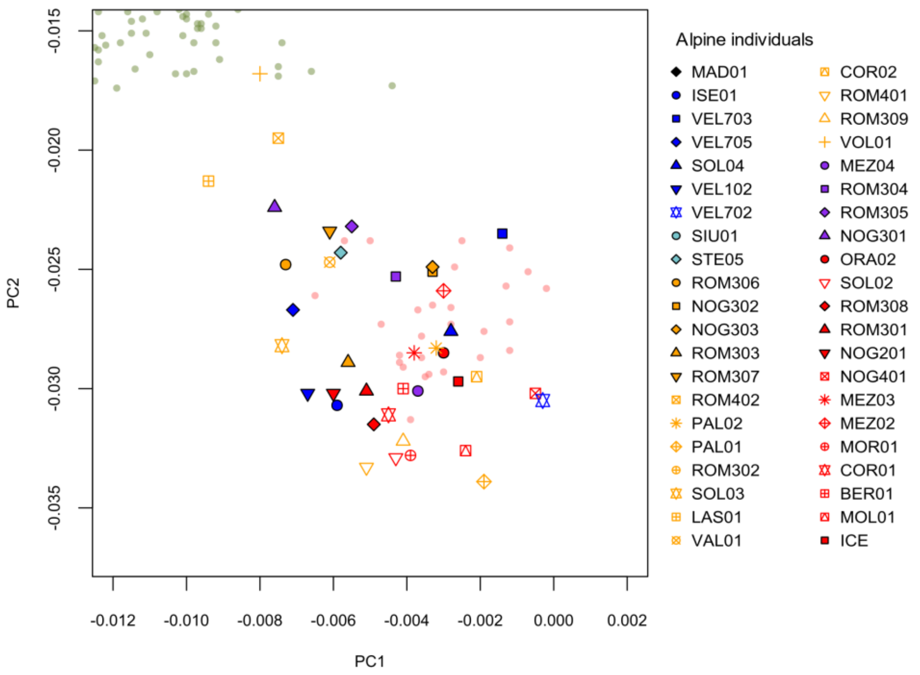


**Figure S19. Principal Component Analysis (PCA).** (**A**) PCA of ancient alpine individuals, projected onto the variation of present-day individuals from West Eurasia. **(B)** Zoom of Fig. A with all alpine prehistoric individuals also marked by different symbols.

A.

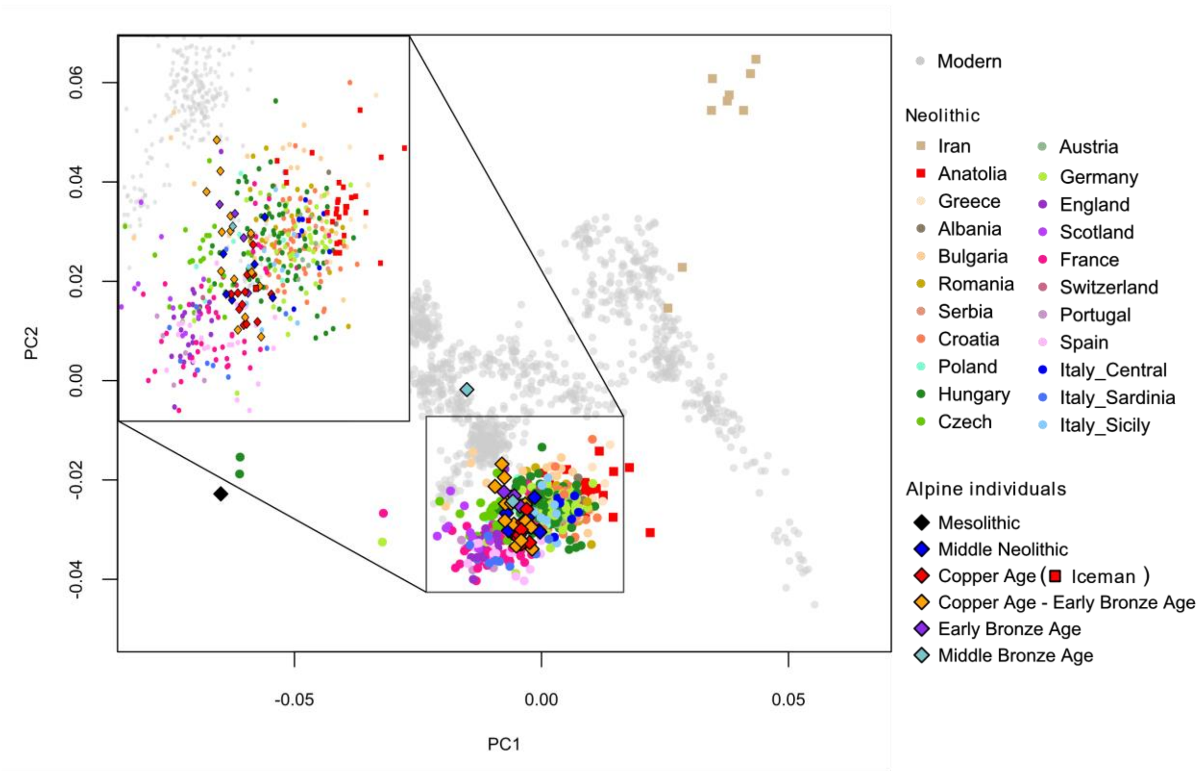
Modern
Neolithic
Iran
Anatolia
Greece
Albania
Bulgaria
Romania
Serbia
Croatia
Poland
Hungary
Czech
Austria
Germany
England
Scotland
France
Switzerland
Portugal
Spain
Italy_Central
Italy_Sardinia
Italy_Sicily
Alpine individuals
Mesolithic
Middle Neolithic
Copper Age ( Iceman )
Copper Age - Early Bronze Age
Early Bronze Age
Middle Bronze Age
PC1
PC2
-0.05
0.00
0.05
-0.04
-0.02
0.00
0.02
0.04
0.06

B.

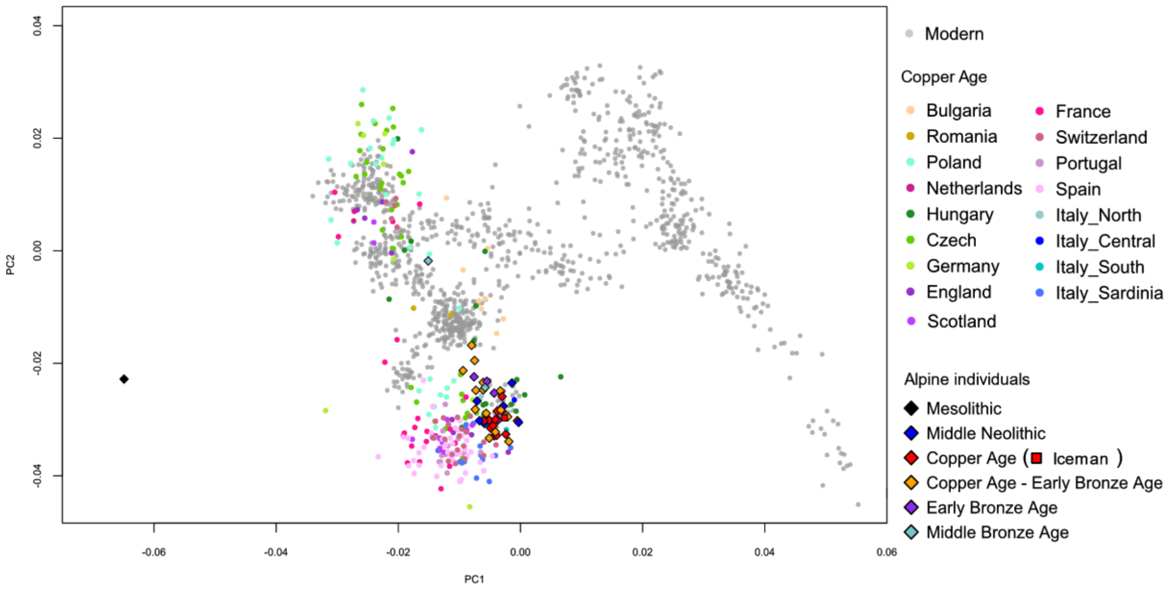
Modern
Copper Age
Bulgaria
Romania
Poland
Netherlands
Hungary
Czech
Germany
England
Scotland
France
Switzerland
Portugal
Spain
Italy_North
Italy_Central
Italy_South
Italy_Sardinia
Alpine individuals
Mesolithic
Middle Neolithic
Copper Age ( Iceman )
Copper Age - Early Bronze Age
Early Bronze Age
Middle Bronze Age
PC1
PC2

C.

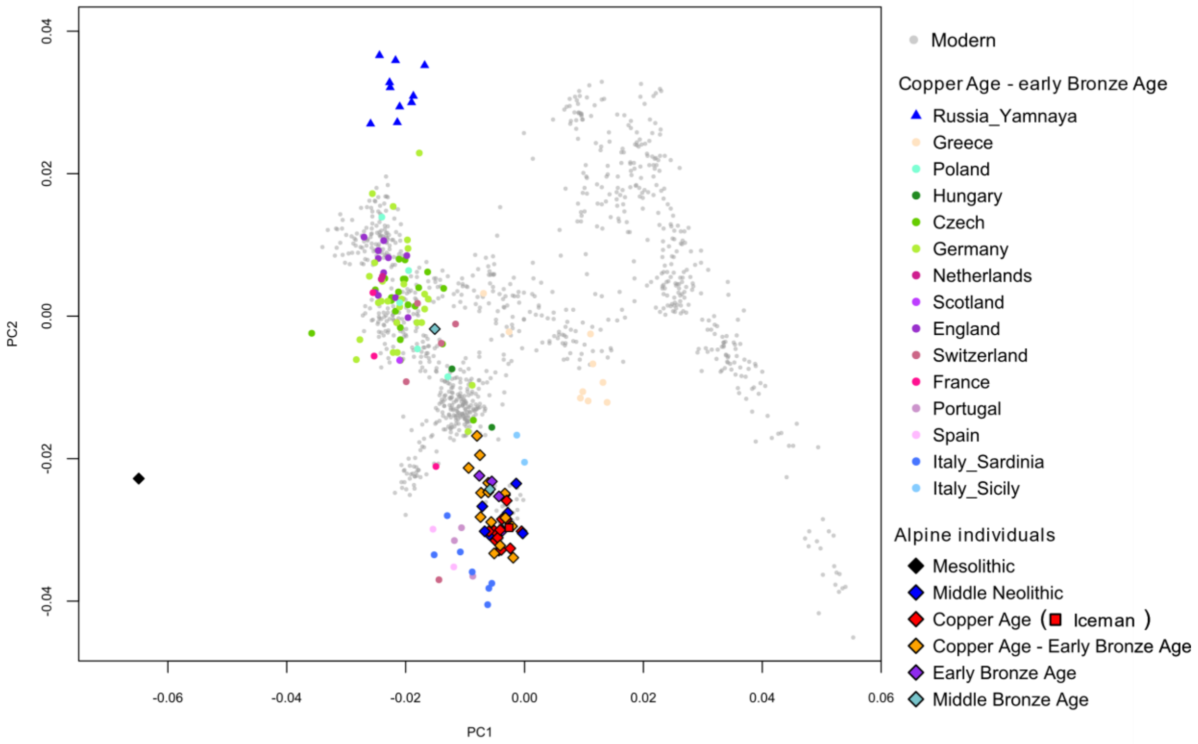


D.

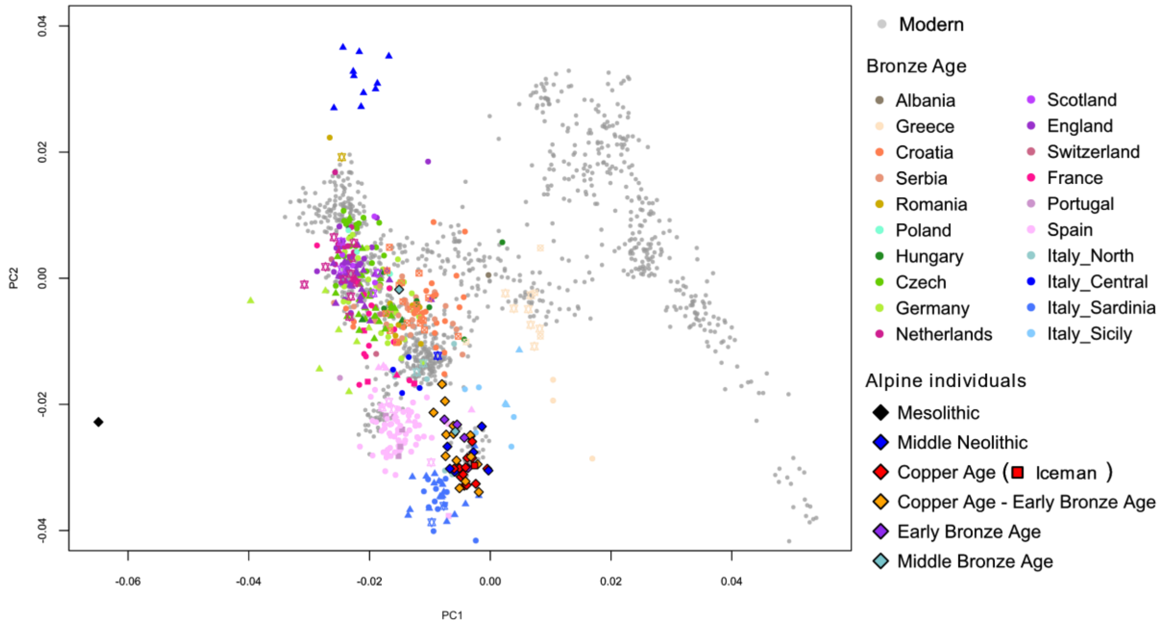


**Figure S20. Principal Component Analyses (PCA)**. PCA of prehistoric alpine individuals from this study and available data from present-day populations and other ancient individuals from West Eurasian and from different chronologies**.** **(A)** Published ancient data are restricted to samples from Neolithic **(B)** published ancient data from Copper Age **(C)** published ancient data from Copper Age-Early Bronze Age and **(D)** published ancient data from Bronze Age.

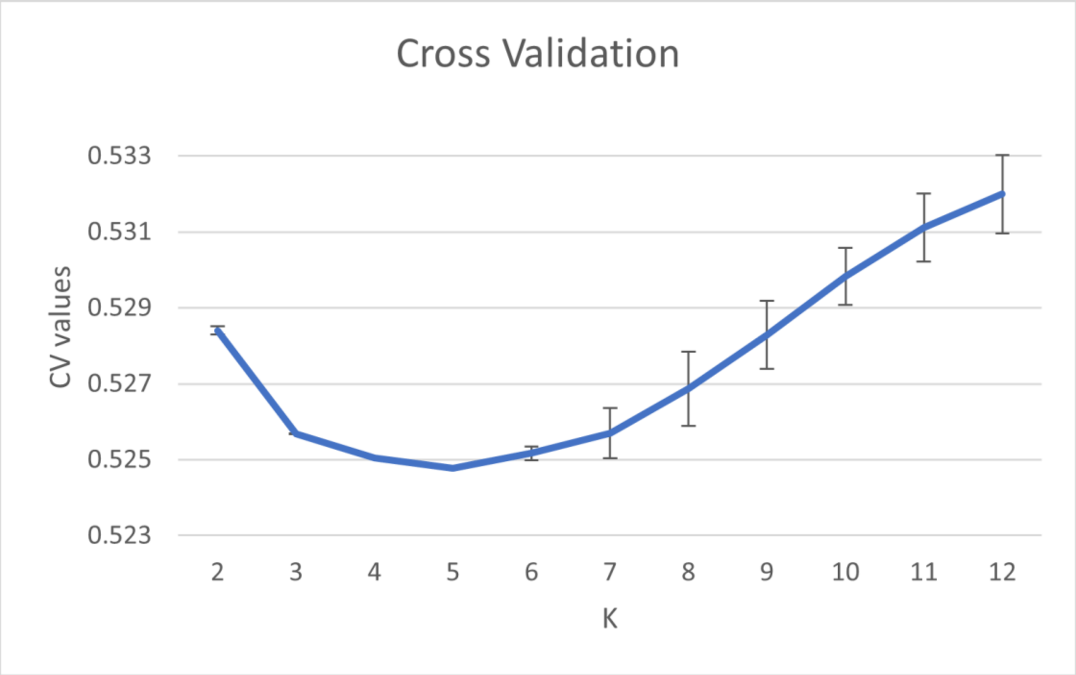


**Figure S21. Cross-validation (cv) values**. Plot of the cross-validation (cv) values for unsupervised clustering analyses by ADMIXTURE (for K=2 to K=12). The standard deviation for each K based on the 10 runs is indicated by the black bar. The lowest cv value indicates the K with lowest error.

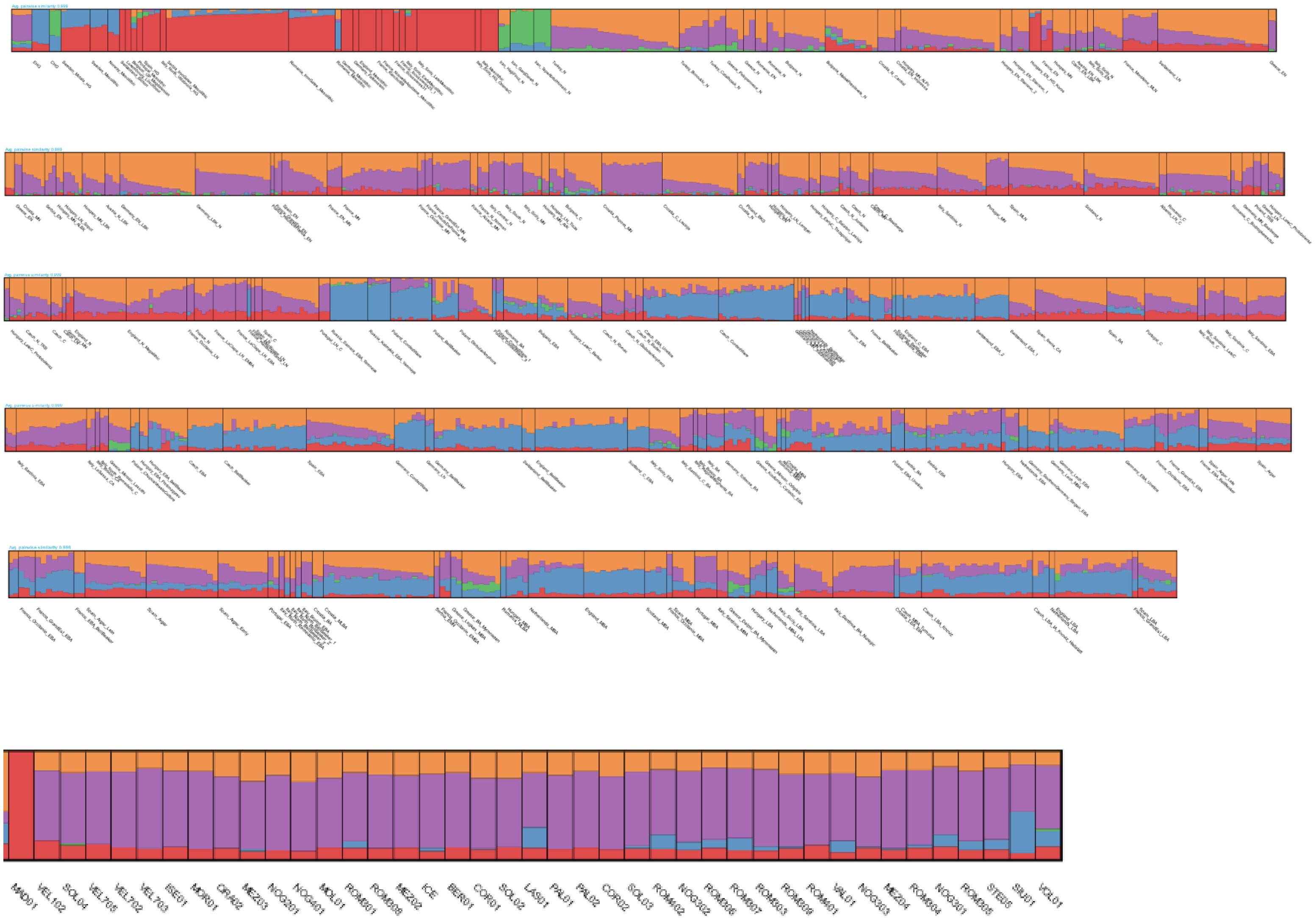


**Figure S22. Unsupervised clustering analyses**. Unsupervised clustering analyses by Admixture (K=5) for 1341 ancient Eurasian individuals (Supplementary Data10) and 41 Prehistoric Alps individuals.

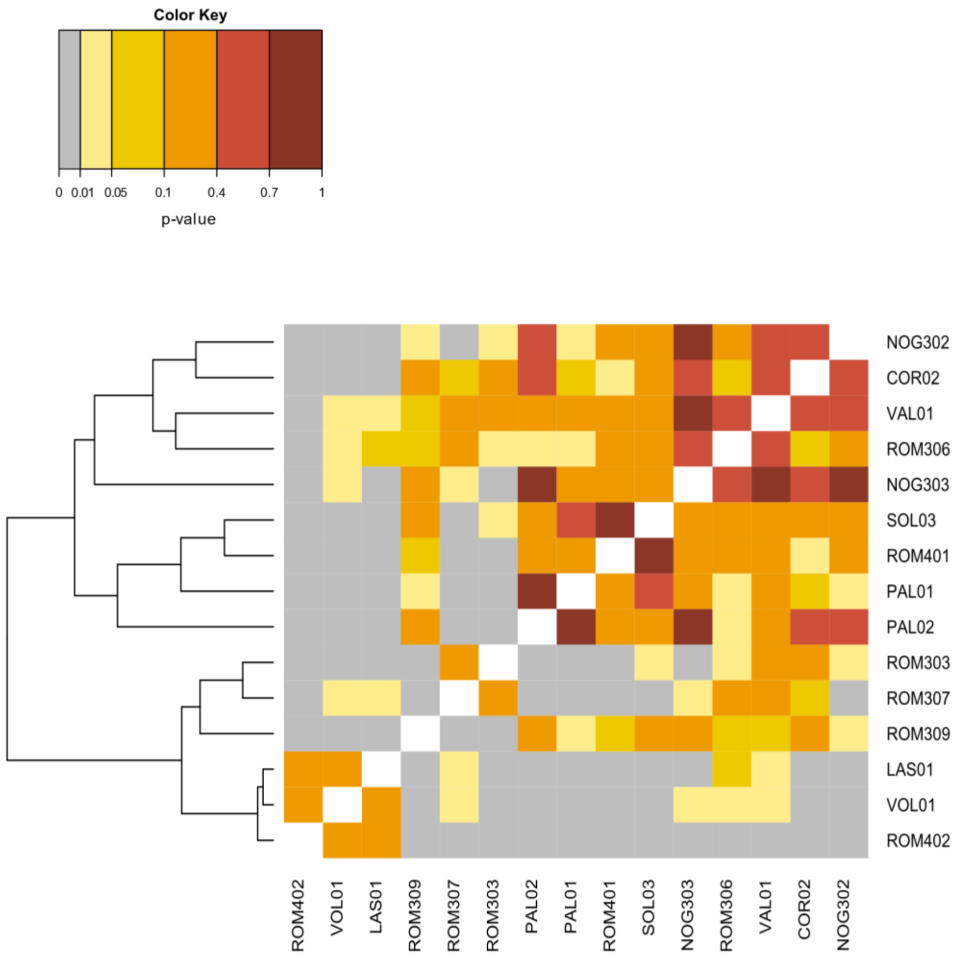


**Figure S23. *Pairwise-qpWave* analysis**. *Pairwise-qpWave* analysis for alpine individuals from Copper Age-Early Bronze Age (CA-EBA) (related to Fig. 3).

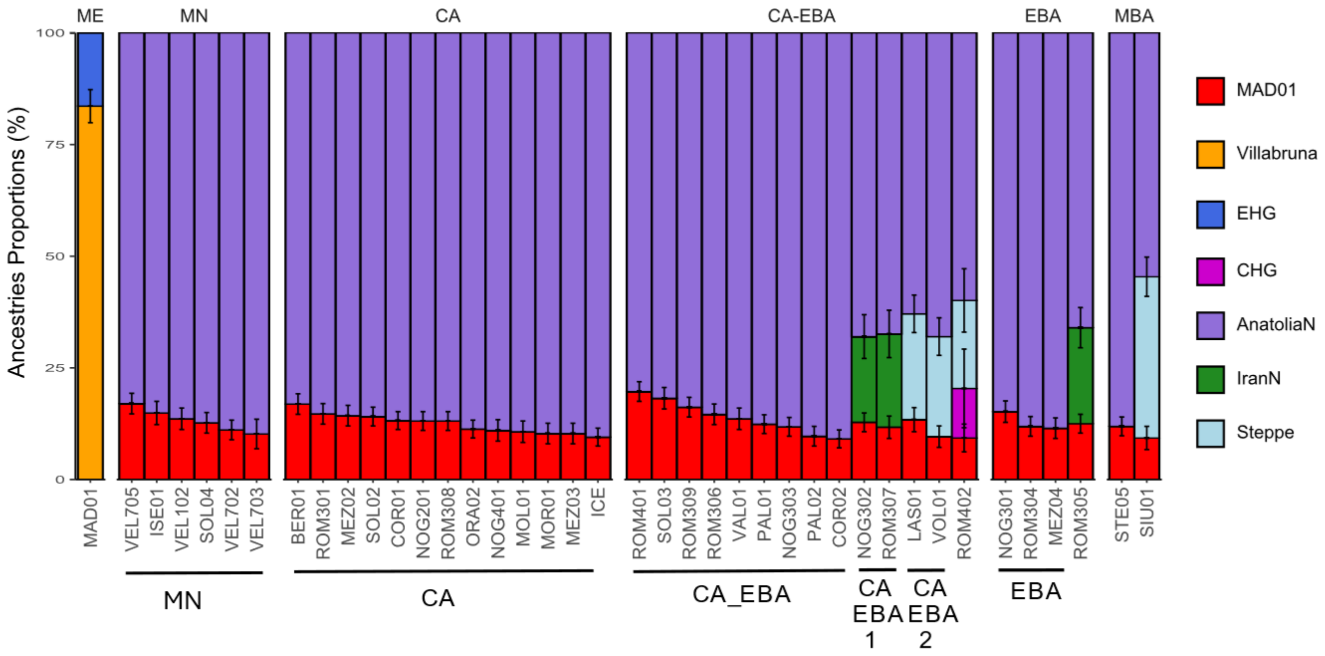


**Figure S24. Definition of alpine groups.** Alpine individuals or groups defined according to their ancestry and chronology (refer to Fig. 4 and Supplementary Data 19).

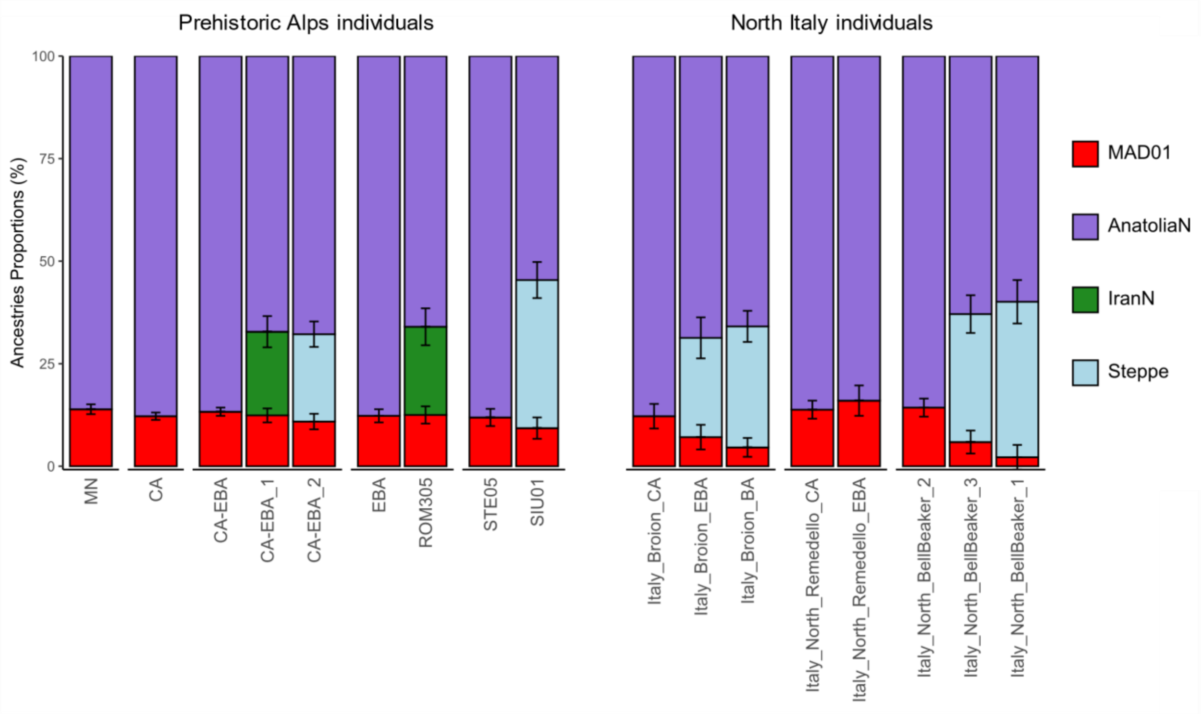


**Figure S25. qpAdm analysis**. qpAdm analysis in prehistoric alpine groups or individuals (left) defined based on their chronologies and ancestries (refer to Fig. 4 and Supplementary Data 19) and in groups from Northern Italy (right).

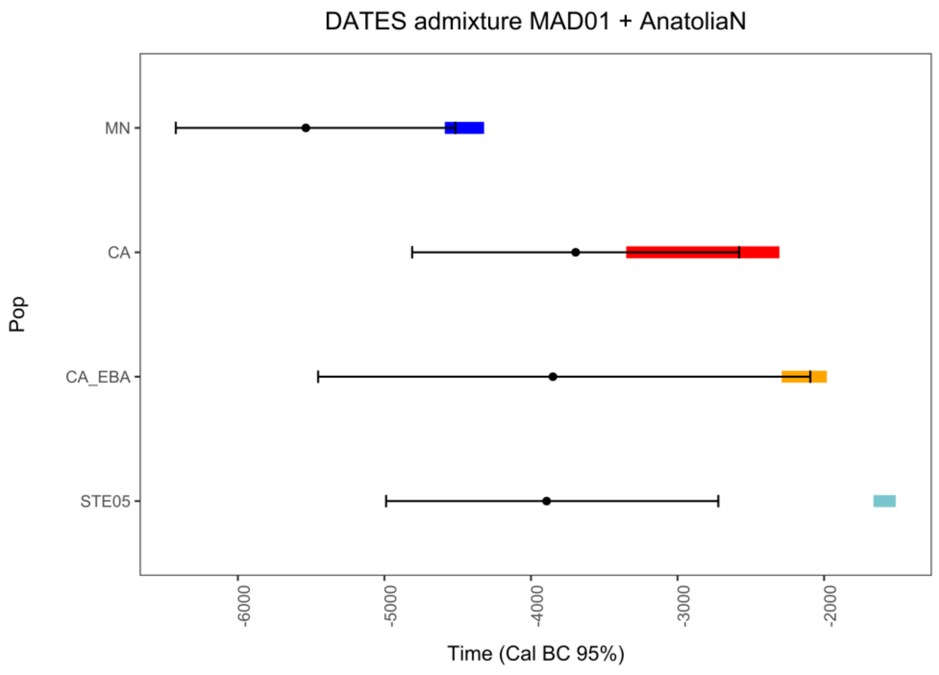


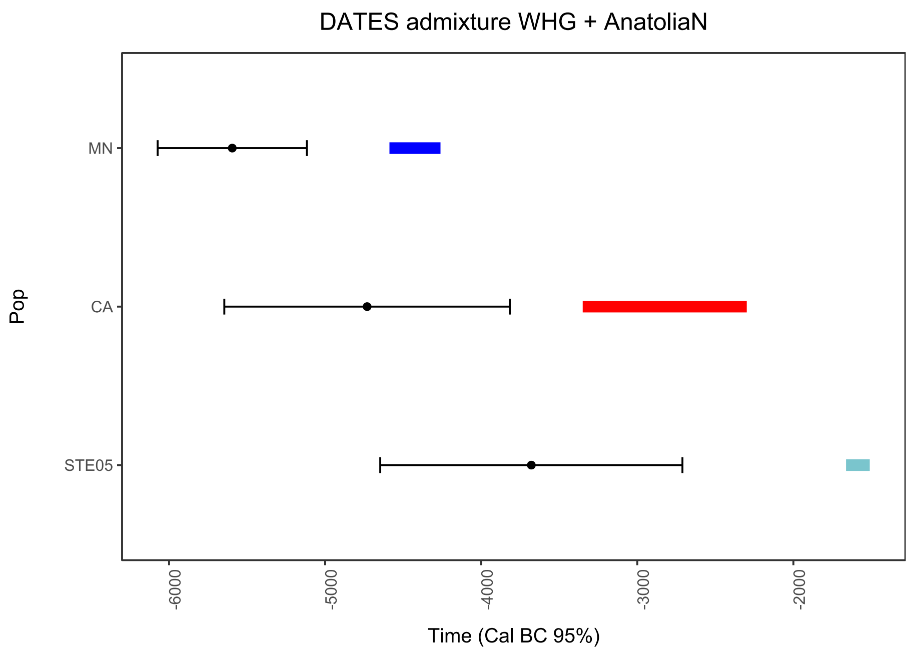


**Figure S26. Estimates of admixture time by DATES.** Timing of the genetic admixture by DATES in groups or individuals defined based on their chronologies and ancestries (Fig. 4 and Supplementary Data 20). We only present results for which it was possible to find a reliable date of admixture between MAD01 alone (top) or a group of WHG (bottom) and the first farmers in NE Anatolia (AnatoliaN) as sources. Furthermore, we only report estimates for which the two-way models of admixture were confirmed by qpAdm analyses (p-value > 0.01) (Supplementary Data 19). The colored rectangles indicate the time frame of the radiocarbon dating of the individuals. The dots and the error bars represent respectively the estimated time of admixture between MAD01 and early NE Anatolian-related ancestries and their standard errors.

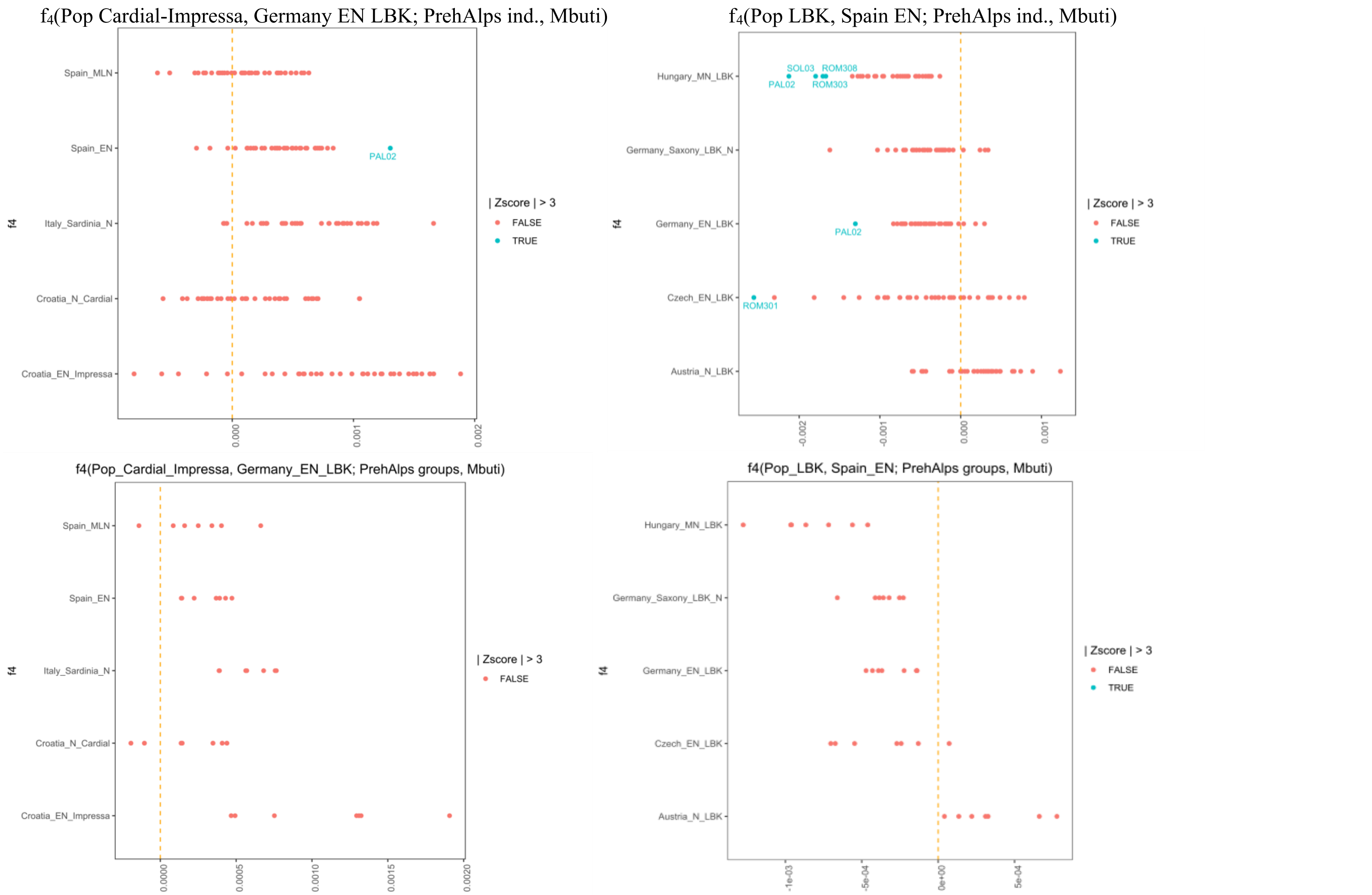


**Figure S27. *$f_4$-stastistic* analysis**. *$f_4$-stastistic* analysis of the form $f_4$(Pop1, Pop2; Ind PrehAlps, Mbuti) for individuals (top) and for groups defined before (bottom). We compared populations associated with the two main Neolithic routes.

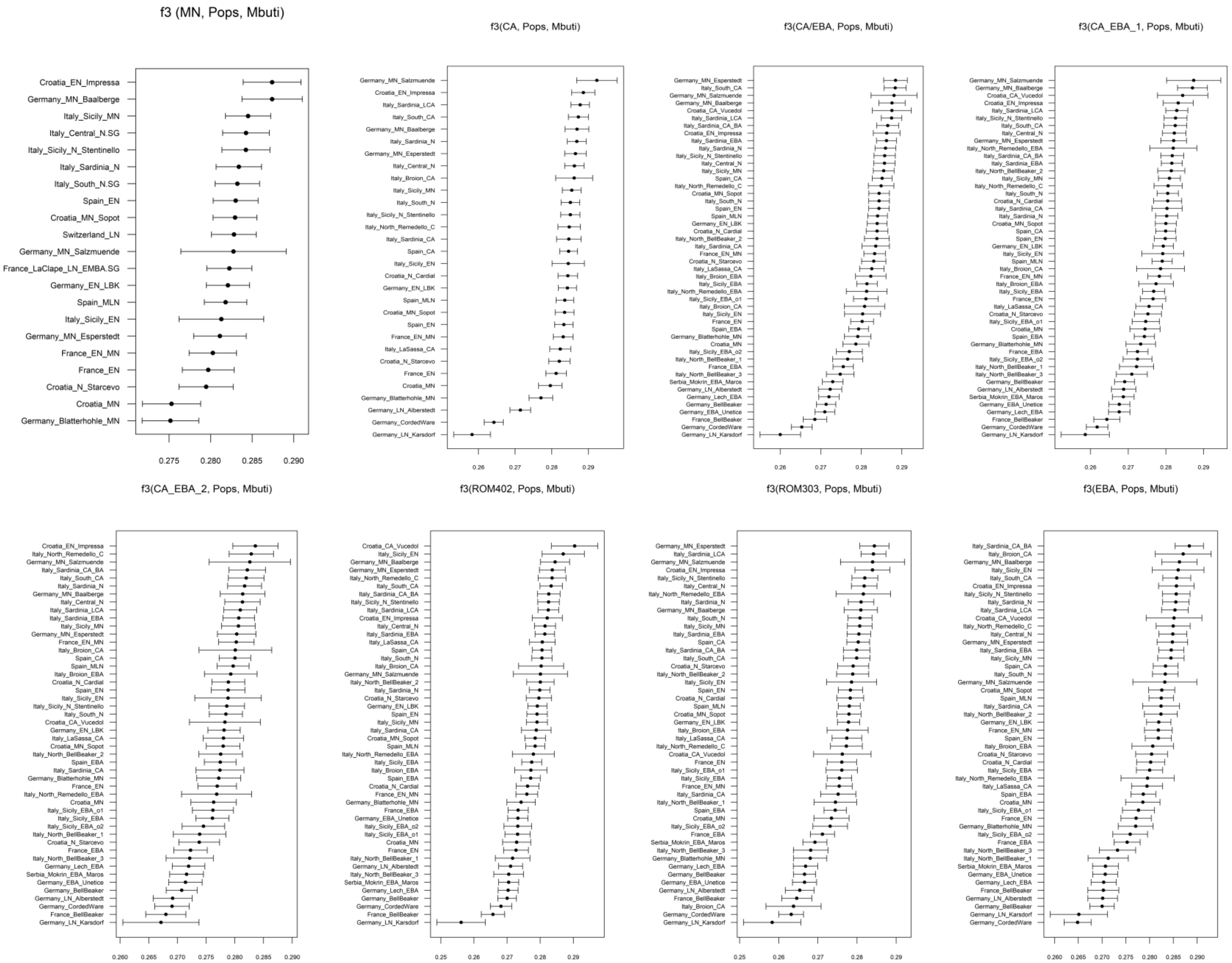
f3 (MN, Pops, Mbuti)
Croatia_EN_Impressa
Germany_MN_Baalberge
Italy_Sicily_MN
Italy_Central_N.SG
Italy_Sicily_N_Stentinello
Italy_Sardinia_N
Italy_South_N.SG
Spain_EN
Croatia_MN_Sopot
Switzerland_LN
Germany_MN_Salzmuende
France_LaClape_LN_EMBA.SG
Germany_EN_LBK
Spain_MLN
Italy_Sicily_EN
Germany_MN_Esperstedt
France_EN_MN
France_EN
Croatia_N_Starcevo
Croatia_MN
Germany_Blatterhohle_MN
0.275 0.280 0.285 0.290
f3(CA, Pops, Mbuti)
f3(CA/EBA, Pops, Mbuti)
f3(CA_EBA_1, Pops, Mbuti)
f3(CA_EBA_2, Pops, Mbuti)
f3(ROM402, Pops, Mbuti)
f3(ROM303, Pops, Mbuti)
f3(EBA, Pops, Mbuti)

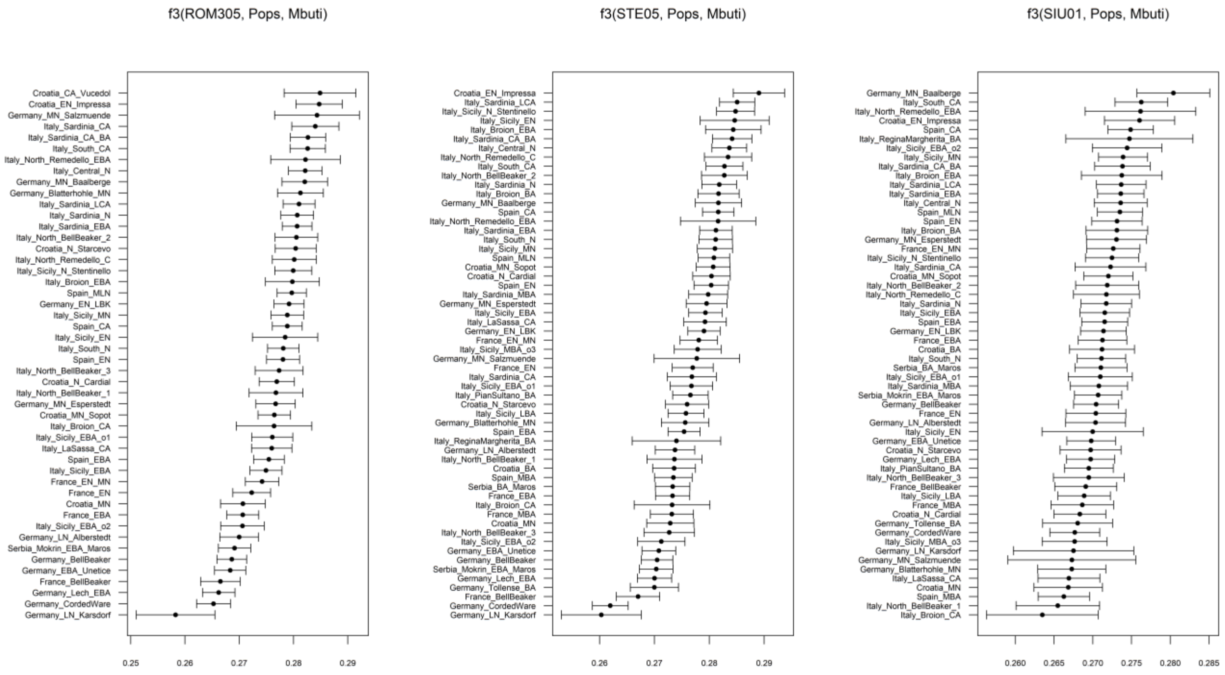


**Figure S28.** ***f3-statistics*** **analysis**. *f3-statistics* analysis of the form *f3*(PrehAlps, Pops, Mbuti). The PrehAlps represent the prehistoric alpine groups or individuals as defined previously. The 'Pops' correspond to European populations from similar or previous time period as the prehistoric alpine groups/individuals analysed and Mbuti is the outgroup population. In each plot, the dot represents the f3 statistics value and the bar the standard error.

**Table SM1:** qpAdm analysis results for best fitting models in each prehistoric alpine individual

| Target | Source 1 | Source 2[a] | Source 3[b] | Source 4 | proportions Source 1 (%) | proportions Source 2 (%) | proportions Source 3 (%) | proportions Source 4 (%) | SE Source 1 (%) | SE Source 2 (%) | SE Source 3 (%) | SE Source 4 (%) | p-value |
|---|---|---|---|---|---|---|---|---|---|---|---|---|---|
| **MAD01** | Villabruna | EHG | - | - | 83.6 | 16.4 | - | - | 3.7 | 3.7 | - | - | 0.145 |
| **VEL102** | MAD01 | Anatolia N | - | - | 13.6 | 86.4 | - | - | 2.4 | 2.4 | - | - | 0.738 |
| **SOL04** | MAD01 | Anatolia N | - | - | 12.7 | 87.3 | - | - | 2.3 | 2.3 | - | - | 0.657 |
| **VEL705** | MAD01 | Anatolia N | - | - | 17 | 83 | - | - | 2.3 | 2.3 | - | - | 0.115 |
| **VEL702** | MAD01 | Anatolia N | - | - | 11.1 | 88.9 | - | - | 2.2 | 2.2 | - | - | 0.039 |
| **VEL703** | MAD01 | Anatolia N | - | - | 10.2 | 89.8 | - | - | 3.3 | 3.3 | - | - | 0.693 |
| **ISE01** | MAD01 | Anatolia N | - | - | 14.9 | 85.1 | - | - | 2.6 | 2.6 | - | - | 0.654 |
| **MEZ02** | MAD01 | Anatolia N | - | - | 14.3 | 85.7 | - | - | 2.3 | 2.3 | - | - | 0.148 |
| **ICE** | MAD01 | Anatolia N | - | - | 9.5 | 90.5 | - | - | 2 | 2 | - | - | 0.123 |
| **MOR01** | MAD01 | Anatolia N | - | - | 10.3 | 89.7 | - | - | 2.3 | 2.3 | - | - | 0.031 |
| **ORA02** | MAD01 | Anatolia N | - | - | 11.3 | 88.7 | - | - | 2 | 2 | - | - | 0.213 |
| **MEZ03** | MAD01 | Anatolia N | - | - | 10.3 | 89.7 | - | - | 2.3 | 2.3 | - | - | 0.77 |
| **NOG201** | MAD01 | Anatolia N | - | - | 13.1 | 86.9 | - | - | 2.1 | 2.1 | - | - | 0.049 |
| **NOG401** | MAD01 | Anatolia N | - | - | 11 | 89 | - | - | 2.4 | 2.4 | - | - | 0.108 |
| **MOL01** | MAD01 | Anatolia N | - | - | 10.7 | 89.3 | - | - | 2.4 | 2.4 | - | - | 0.028 |
| **ROM301** | MAD01 | Anatolia N | - | - | 14.7 | 85.3 | - | - | 2.3 | 2.3 | - | - | 0.134 |
| **ROM308** | MAD01 | Anatolia N | - | - | 13.1 | 86.9 | - | - | 2.1 | 2.1 | - | - | 0.903 |
| **BER01** | MAD01 | Anatolia N | - | - | 16.9 | 83.1 | - | - | 2.3 | 2.3 | - | - | 0.603 |
| **COR01** | MAD01 | Anatolia N | - | - | 13.2 | 86.8 | - | - | 2 | 2 | - | - | 0.661 |

| | | | | | | | | | | | | | |
|---|---|---|---|---|---|---|---|---|---|---|---|---|---|
| **SOL02** | MAD01 | Anatolia N | - | - | 14.1 | 85.9 | - | - | 2.1 | 2.1 | - | - | 0.076 |
| **ROM309** | MAD01 | Anatolia N | - | - | 16.2 | 83.8 | - | - | 2.2 | 2.2 | - | - | 0.071 |
| **ROM401** | MAD01 | Anatolia N | - | - | 19.7 | 80.3 | - | - | 2.2 | 2.2 | - | - | 0.379 |
| **LAS01** | MAD01 | Anatolia N | Steppe | - | 13.4 | 62.9 | 23.7 | - | 2.7 | 3.8 | 4.2 | - | 0.874 |
| **PAL01** | MAD01 | Anatolia N | - | - | 12.4 | 87.6 | - | - | 2.1 | 2.1 | - | - | 0.262 |
| **PAL02** | MAD01 | Anatolia N | - | - | 9.7 | 90.3 | - | - | 2.2 | 2.2 | - | - | 0.369 |
| **COR02** | MAD01 | Anatolia N | - | - | 9.1 | 90.9 | - | - | 2 | 2 | - | - | 0.276 |
| **SOL03** | MAD01 | Anatolia N | - | - | 18.2 | 81.8 | - | - | 2.4 | 2.4 | - | - | 0.603 |
| **ROM402** | MAD01 | Anatolia N | Steppe | CHG | 9.3 | 59.9 | 19.7 | 11.1 | 3.1 | 6.4 | 7.1 | 8.8 | 0.016 |
| **NOG302** | MAD01 | Anatolia N | IranN | - | 12.8 | 68 | 19.2 | - | 2.1 | 5.9 | 4.9 | - | 0.224 |
| **ROM306** | MAD01 | Anatolia N | - | - | 14.6 | 85.4 | - | - | 2.3 | 2.3 | - | - | 0.29 |
| **ROM307** | MAD01 | Anatolia N | IranN | | 11.7 | 67.4 | 20.9 | - | 2.5 | 6.5 | 5.3 | - | 0.66 |
| **VAL01** | MAD01 | Anatolia N | - | - | 13.6 | 86.4 | - | - | 2.4 | 2.4 | - | - | 0.189 |
| **NOG303** | MAD01 | Anatolia N | - | - | 11.8 | 88.2 | - | - | 2.1 | 2.1 | - | - | 0.61 |
| **VOL01** | MAD01 | Anatolia N | Steppe | - | 9.6 | 68 | 22.4 | - | 2.4 | 3.9 | 4.2 | - | 0.05 |
| **MEZ04** | MAD01 | Anatolia N | - | - | 11.5 | 88.5 | - | - | 2.3 | 2.3 | - | - | 0.066 |
| **ROM304** | MAD01 | Anatolia N | - | - | 11.9 | 88.1 | - | - | 2.2 | 2.2 | - | - | 0.319 |
| **NOG301** | MAD01 | Anatolia N | - | - | 15.2 | 84.8 | - | - | 2.4 | 2.4 | - | - | 0.627 |
| **ROM305** | MAD01 | Anatolia N | IranN | - | 12.5 | 66 | 21.5 | - | 2.1 | 5.3 | 4.5 | - | 0.109 |
| **STE05** | MAD01 | Anatolia N | - | - | 11.9 | 88.1 | - | - | 2.1 | 2.1 | - | - | 0.174 |
| **SIU01** | MAD01 | Anatolia N | Steppe | - | 9.3 | 54.6 | 36.1 | - | 2.6 | 3.6 | 4.4 | - | 0.115 |

[a] EHG = East Hunter-Gatherer; AnatoliaN = early Neolithic Anatolian farmers

[b] IranN = Neolithic Iranian; Steppe = Yamnaya herders from Pontic Steppe

[c] The *p-value* was calculated using a likelihood ratio test in which the constrained model is the null hypothesis and the unconstrained model is the alternative hypothesis.

CHGs = Caucasus Hunter Gatherers

**Table SM2.** qpAdm analysis results for best fitting models in prehistoric alpine groups

| target[a] | Source 1 | Source 2 | Source 3 | Source 4 | proportions Source 1 (%) | proportions Source 2 (%) | proportions Source 3 (%) | proportions Source 4 (%) | SE Source 1 (%) | SE Source 2 (%) | SE Source 3 (%) | SE Source 4 (%) | P value |
|---|---|---|---|---|---|---|---|---|---|---|---|---|---|
| **MN** | MAD01 | Anatolia N | - | - | 13.9 | 86.1 | - | - | 1.2 | 1.2 | - | - | 0.958 |
| **CA** | MAD01 | Anatolia N | - | - | 12.2 | 87.8 | - | - | 0.9 | 0.9 | - | - | 0.011 |
| **CA_EBA** | MAD01 | Anatolia N | - | - | 13.3 | 86.7 | - | - | 1 | 1 | - | - | 0.022 |
| **CA_EBA_1** | MAD01 | Anatolia N | IranN | - | 12.4 | 67.2 | 20.4 | - | 1.7 | 4.6 | 3.8 | - | 0.9821 |
| **CA_EBA_2** | MAD01 | Anatolia N | Steppe | - | 10.9 | 67.8 | 21.3 | - | 1.9 | 2.8 | 3.1 | - | 0.18 |
| **EBA** | MAD01 | Anatolia N | - | - | 12.3 | 87.8 | - | - | 1.6 | 1.6 | - | - | 0.021 |

[a] See Fig. S24 for details about individuals included in each group. MN = Middle Neolithic; CA = Copper Age; CA_EBA = Copper Age – early Bronze Age; EBA = Early Bronze Age.

[b] The *p-value* was calculated using a likelihood ratio test in which the constrained model is the null hypothesis and the unconstrained model is the alternative hypothesis.
