# Supplementary material for: Genomic diversity and structure of prehistoric alpine individuals from the Tyrolean Iceman’s territory
Source: Nat Commun. 2025 Jul 11;16:6431. doi: 10.1038/s41467-025-61601-8 (PMC12254411; doi:10.1038/s41467-025-61601-8)
Supplement: Supplementary file 2 — Description of Additional Supplementary Files [file 41467_2025_61601_MOESM2_ESM.pdf]

Description of Additional Supplementary Files

File Name: Supplementary Data 1

Description: Archaeological, anthropological and dating information on the alpine individuals analysed in this study and from the literature (ICE: Wang et al. 2023; ORA01 amd ORA02: Paladin et al. 2023).

File Name: Supplementary Data 2

Description: Results of the molecular screening

File Name: Supplementary Data 3

Description: Results of the capture analysis

File Name: Supplementary Data 4

Description: Final dataset of the alpine individuals used for downstream analyses

File Name: Supplementary Data 5

Description: Results of kinship analyses based on the three methods applied in this study (lcMLkin, READ and TKGWV2)

File Name: Supplementary Data 6

Description: Results of the kinship analysis based on the TKGWV2 method

File Name: Supplementary Data 7

Description: Results of the kinship analysis based on the READ method

File Name: Supplementary Data 8

Description: Results of the kinship analysis based on the KIN method

File Name: Supplementary Data 9

Description: Results of the Run of Homozygosity (ROH) analysis

File Name: Supplementary Data 10

Description: List of the Western Eurasian individuals used for PCA and ADMIXTURE analyses (refer to Methods, Datasets).

File Name: Supplementary Data 11

Description: List of Paleolithic and Mesolithic individuals used for MDS analysis (based on the publication from Posth et al. 2023: doi.org/10.1038/s41586-023-05726-0).

File Name: Supplementary Data 12

Description: Results of f3 statistics: f3(pop1, pop2; Mbuti)

File Name: Supplementary Data 13

Description: Results of Outgroup f3 statistics: f3(PopA, PopB; Mbuti)

File Name: Supplementary Data 14

Description: Results of f4-statistics analysis of the form f4(Pop1, Pop2; Ind PrehAlps, Mbuti) for each prehistoric alpine individual (test)

File Name: Supplementary Data 15

Description: Results of f4-statistics analysis of the form f4(Pop1, Pop2; Group PrehAlps, Mbuti) for alpine groups (test) defined based on their ancestry

Description: Results for f4-statistics analysis of the form f4(Pop1, Pop2; MN, Mbuti) with pop1 = Villabruna and Mesolithic groups from Italy and South-east Europe.

File Name: Supplementary Data 16

Description: Results of qpAdm analysis for the Mesolithic alpine individual (MAD01).

File Name: Supplementary Data 17

Description: Results of qpAdm analysis for 2-way and 3-way models for each prehistoric alpine individual

File Name: Supplementary Data 18

Description: Results of qpAdm analysis for 2-way models for prehistoric alpine individuals with steppe-related ancestry or Iran Neolithic-related ancestry. Alpine groups used as one of the source are defined based on their ancestry and chronology (Figure S24)

File Name: Supplementary Data 19

Description: Results of qpAdm analysis for 2-way and 3-way models for alpine individuals/groups from this study and on other ancient individuals/groups from Italy. Alpine groups are defined based on their ancestry and chronology (Figure S24)

File Name: Supplementary Data 20

Description: Results on estimated admixture times obtained by DATES (One generation= 28 years)

File Name: Supplementary Data 21

Description: Results of phenotypic analysis based on the Hirisplex list of SNPs. On the bottom is indicated the phenotype estimated for the eye, hair and skin color based on the p-value and the AUC loss (area under the curve). NA = the phenotypic trait could not be determined

File Name: Supplementary Data 22

Description: List of phenotypic SNPs analysed in this study base on the list from Wang et al. 2023 (doi.org/10.1016/j.xgen.2023.100377)

File Name: Supplementary Data 23

Description: Reagents used in this study with company names and catalogue numbers
