# Supplementary material for: Genomic diversity and structure of prehistoric alpine individuals from the Tyrolean Iceman’s territory
Source: Nat Commun. 2025 Jul 11;16:6431. doi: 10.1038/s41467-025-61601-8 (PMC12254411; doi:10.1038/s41467-025-61601-8)
Supplement: Supplementary file 4 — Reporting Summary [file 41467_2025_61601_MOESM4_ESM.pdf]

nature portfolio

Corresponding author(s): NCOMMS-24-36149B

Last updated by author(s): May 15, 2025

Reporting Summary

Nature Portfolio wishes to improve the reproducibility of the work that we publish. This form provides structure for consistency and transparency in reporting. For further information on Nature Portfolio policies, see our Editorial Policies and the Editorial Policy Checklist.

Statistics

For all statistical analyses, confirm that the following items are present in the figure legend, table legend, main text, or Methods section.

n/a	Confirmed	
☐	☒	The exact sample size (*n*) for each experimental group/condition, given as a discrete number and unit of measurement
☐	☒	A statement on whether measurements were taken from distinct samples or whether the same sample was measured repeatedly
☐	☒	The statistical test(s) used AND whether they are one- or two-sided<br>*Only common tests should be described solely by name; describe more complex techniques in the Methods section.*
☒	☐	A description of all covariates tested
☐	☒	A description of any assumptions or corrections, such as tests of normality and adjustment for multiple comparisons
☐	☒	A full description of the statistical parameters including central tendency (e.g. means) or other basic estimates (e.g. regression coefficient) AND variation (e.g. standard deviation) or associated estimates of uncertainty (e.g. confidence intervals)
☐	☒	For null hypothesis testing, the test statistic (e.g. *F*, *t*, *r*) with confidence intervals, effect sizes, degrees of freedom and *P* value noted<br>*Give P values as exact values whenever suitable.*
☒	☐	For Bayesian analysis, information on the choice of priors and Markov chain Monte Carlo settings
☒	☐	For hierarchical and complex designs, identification of the appropriate level for tests and full reporting of outcomes
☒	☐	Estimates of effect sizes (e.g. Cohen's *d*, Pearson's *r*), indicating how they were calculated

Our web collection on statistics for biologists contains articles on many of the points above.

Software and code

Policy information about availability of computer code

Data collection

Data produced in this study:

-Shotgun data for 52 alpine prehistoric individuals. Paired-end genomic libraries of DNA extracts from the 52 individuals sequenced in an external company (Macrogen) by Illumina HiSeq-X system.
-Capture data from 47 selected individuals after shotgun sequencing. The samples (with more than 1% of HR) were enriched (kit myBaits ® Expert Human Affinities – Prime Plus, Arbor Bioscience) and sequenced in an external company (Macrogen) by Illumina HiSeq-X system.
-Radiocarbon dating (14C) of initial 36 alpine individuals (final N=34) performed at the Curt-Engelhorn-Center Archaeometry gGmbH (CEZA, Mannheim, Germany, https://ceza.de/en).

Public genomic data used for the analyses:

-AADR v54.1 (Mallick, S. et al. The Allen Ancient DNA Resource (AADR) a curated compendium of ancient human genomes. Sci Data 11, (2024). Web source: https://reich.hms.harvard.edu/datasets
- Ancient genomes from Posth, C. et al. Palaeogenomics of Upper Palaeolithic to Neolithic European hunter-gatherers. Nature 615, 117–126 (2023). Available at European Nucleotide Archive (ENA) under study accession number PRJEB51862.

Data analysis

OxCal (v.4.4)
BWA (v.0.7.17)
Dedup (v.0.12.1)
mapDamage2 (v.2.2.1)
SAMtools (v.1.16.1)

PMDTools (v.0.60)
PileupCaller (https://github.com/stschiff/sequenceTools)
ANGSD (v.0.941)
bcftools call (v.1.16)
PEAR (v.0.9.10)
Schmutzi (v.1.5.6)
HaploGrep 2 (v.2.4.0)
Yleaf (v.2.2)
hapROH (v0.63)
READ v.1
TKGWV2 (v.2)
KIN (v.3.1.3)
smartpca (v16000)
ADMIXTURE (v.1.3.0)
PLINK (v.1.9)
pong (v.1.4.9)
ADMIXTOOLS (v.7.0.2)
R-package gplots
R package admixr (v.1.0.0)
DATES (v.4010)
Hirisplex panel (https://hirisplex.erasmusmc.nl/)

For manuscripts utilizing custom algorithms or software that are central to the research but not yet described in published literature, software must be made available to editors and reviewers. We strongly encourage code deposition in a community repository (e.g. GitHub). See the Nature Portfolio guidelines for submitting code & software for further information.

Data

Policy information about availability of data

All manuscripts must include a data availability statement. This statement should provide the following information, where applicable:

- Accession codes, unique identifiers, or web links for publicly available datasets
- A description of any restrictions on data availability
- For clinical datasets or third party data, please ensure that the statement adheres to our policy

The raw sequences data (FASTQ files) generated in this study have been submitted at the European Nucleotide Archive (ENA: https://www.ebi.ac.uk/ena/browser/home) with the accession number PRJEB70242 and are public at the time of submission of this document. Access codes to previously published data: AADR v54.1 database [https://reich.hms.harvard.edu/datasets] and dataset from [https://doi.org/10.1038/s41586-023-05726-0; ENA accession number PRJEB51862].

Research involving human participants, their data, or biological material

Policy information about studies with human participants or human data. See also policy information about sex, gender (identity/presentation), and sexual orientation and race, ethnicity and racism.

Reporting on sex and gender	We assign the biological sex (XX or XY) of the ancient individuals analysed (see Methods). Then, our genetic findings do not provide any information on the gender identity of these individuals.
Reporting on race, ethnicity, or other socially relevant groupings	These information are not available for ancient individuals.
Population characteristics	These information are not available for ancient individuals.
Recruitment	After the authorisations by the Provincial authorities that protect the archaeological heritage in this area such as the Provincia Autonoma di Trento (Prot. n. 156 15.03.2021) and Provincia Autonoma di Bolzano/Bozen (Prot. Nr. 636137-25.09.2019), the human remains were collected from various Institutions scattered throughout the territory (after further authorizations by the specific Institution) such as: MUSE of Trento, Castello del Buonconsiglio of Trento, the Museo di Scienze e Archaeologia- Fondazione Museo Civico di Rovereto, the Soprintendenza per i Beni Archeologici della Provincia Autonoma di Trento and the repository of the Soprintendenza Archeologica della Provincia Autonoma di Bolzano/Bozen.<br>After photographic documentation, (before and after sampling), the bones were sampled using a minimally invasive method. For the Pars Petrosa (PP), a thin-tipped drill was used to create a small hole in the cortical bone that forms the structure of the cochlea (part of the osseous labyrinth of the inner ear). This approach minimized the invasiveness of the sampling avoiding the cutting and the destruction of the PP and making it accessible for further (e.g. macroscopic) studies. Regarding the tooth sampling, the root was separated from the crown using a Dremel and then milled. Using the root, the pulp chamber was also included, which preserves microbial DNA, making DNA samples available for further investigations.
Ethics oversight	The study was approved by the local Provincial authorities: Provincia Autonoma di Trento (Prot. n. 156 15.03.2021) and Provincia Autonoma di Bolzano/Bozen (Prot. Nr. 636137- 25.09.2019). See also "Recruitment" section above.

Note that full information on the approval of the study protocol must also be provided in the manuscript.

Field-specific reporting

Please select the one below that is the best fit for your research. If you are not sure, read the appropriate sections before making your selection.

☒ Life sciences ☐ Behavioural & social sciences ☐ Ecological, evolutionary & environmental sciences

For a reference copy of the document with all sections, see nature.com/documents/nr-reporting-summary-flat.pdf

Life sciences study design

All studies must disclose on these points even when the disclosure is negative.

Sample size	No a priori calculation of sample size was made in this study. The number of prehistoric individuals analysed in our study was determined by the actual available number of rare ancient human specimens that also had to be suitable for genetic analysis (with well-preserved teeth or Pars Petrosa).
Data exclusions	After a first molecular screening of samples from 52 ancient individuals, 4 individuals were excluded due to the low preservation of their human endogenous DNA (< than 1%). The other individuals (N=47), were further analyzed by capture method, except one which was not anymore available for the analysis. After capture, one individual which showed a very low deamination value was excluded from the study. Moreover, 3 individuals were found to be contaminated. However, after filtering the contaminated reads with PMDTools (see Methods) , only one sample was discarded due to the small number of remaining SNPs (< 20,000 SNPs sites on the "1240K panel" SNPs).<br>Please refer to the Supplementary Information Text S3 and to the Supplementary Data 1-4 for more details.
Replication	Genetic results were reproduced for many individuals, producing multiple DNA libraries for a single individual and multiple analyses that yielded consistent results (e.g. genetic relatedness, mtDNA haplogroup and Y-Chromosome assignment, kinships analyses).
Randomization	Randomization is not relevant to this study. Individuals are grouped based on sampling locations, dates and genetic affinities.
Blinding	Blinding is not applicable for ancient specimens (see also sample size section above).

Reporting for specific materials, systems and methods

We require information from authors about some types of materials, experimental systems and methods used in many studies. Here, indicate whether each material, system or method listed is relevant to your study. If you are not sure if a list item applies to your research, read the appropriate section before selecting a response.

Materials & experimental systems

n/a	Involved in the study
☒	☐ Antibodies
☒	☐ Eukaryotic cell lines
☐	☒ Palaeontology and archaeology
☒	☐ Animals and other organisms
☒	☐ Clinical data
☒	☐ Dual use research of concern
☒	☐ Plants

Methods

n/a	Involved in the study
☒	☐ ChIP-seq
☒	☐ Flow cytometry
☒	☐ MRI-based neuroimaging

Palaeontology and Archaeology

Specimen provenance	The exact geographical provenance (i.e. site location, valley and province) of the sampled human remains for this project has been detailed in various parts of the main text (i.e. Figure 1) as well as in the Supplementary Data 1-2 and in Supplementary Information Text S1. With regard to authorisations by issuing authorities, these are described in Supplementary Information Text S1, in the "Recruitment" section of this document and in the manuscript (In Methods and in the Data Availability and Acknowledgments sections). The Soprintendenza of the Autonomous Province of Trento (Prot. n. 156 15.03.2021) and the Autonomous Province of Bolzano /Bozen (Prot. Nr. 636137-25.09.2019) have authorized the study and scientific analyses of the human remains sampled for this project.
Specimen deposition	The human specimens are deposited in the following museums: MUSE of Trento (https://www.muse.it/en/), Castello del Buonconsiglio in Trento (https://www.buonconsiglio.it/en/), Museo di Scienze e Archaeologia, Fondazione Museo Civico di Rovereto (https://www.fondazionemcr.it/context.jsp?ID_LINK=114891&area=279) and the Univerista´di Trento, Dipartimento di Lettere e Filosofia (https://www.lettere.unitn.it/) and are accessible with prior authorization from the relevant authorities. Moreover, part of the osteological remains of the individuals sampled in this study are housed at the repositories of the Soprintendenza per i Beni Archeologici della Provincia Autonoma di Trento (https://www.provincia.tn.it/Amministrazione/Strutture-organizzative/Umst-soprintendenza-per-i-beni-e-le-attivita-culturali) and Ufficio Archaeologico, Provincia Autonoma di Bolzano/Bozen (https://home.provincia.bz.it/it/contatti/580).

Dating methods

Radiocarbon dating (14C) of 36 alpine individuals were conducted at the Curt-Engelhorn-Center Archaeometry gGmbH (CEZA, Mannheim, Germany, https://ceza.de/en). Details on the sample selection and protocol can be found in the Supplementary Information Text S2.

☒ Tick this box to confirm that the raw and calibrated dates are available in the paper or in Supplementary Information.

Ethics oversight

Authorisations with prescriptions were obtained for the study, sampling, and temporary transfer of osteological finds owned by the Provincia Autonoma di Trento and the Provincia Autonoma di Bolzano/Bozen, as well as for genetic analyses within the project ‘Genomic diversity of prehistoric individuals from the Iceman's territory in the Eastern Italian Alps’ (see also the sections ‘Ethics oversight’ and ‘Recruitment’ for details on the protocols).

Note that full information on the approval of the study protocol must also be provided in the manuscript.

Plants

Seed stocks

Plants are not considered in this study.

Novel plant genotypes

Plants are not considered in this study.

Authentication

Plants are not considered in this study.
